# Tröger’s Base Network Polymers of Intrinsic Microporosity (TB-PIMs) with Tunable Pore Size for Heterogeneous Catalysis

**DOI:** 10.1021/jacs.2c04739

**Published:** 2022-08-16

**Authors:** Ariana R. Antonangelo, Natasha Hawkins, Elena Tocci, Chiara Muzzi, Alessio Fuoco, Mariolino Carta

**Affiliations:** † Department of Chemistry, Faculty of Science and Engineering, Swansea University, Grove Building, Singleton Park, Swansea SA2 8PP, U.K.; ‡ Institute on Membrane Technology, National Research Council of Italy (CNR-ITM), via P. Bucci 17/C, Rende (CS) 87036, Italy

## Abstract

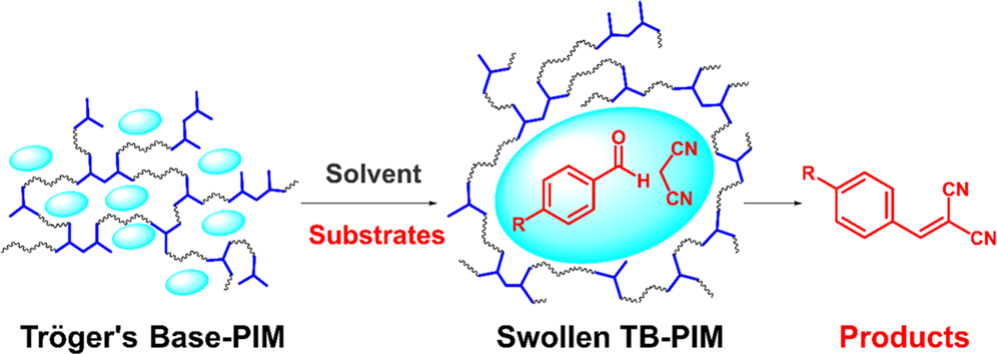

Heterogeneous catalysis plays a pivotal role in the preparation of value-added chemicals, and it works more efficiently when combined with porous materials and supports. Because of that, a detailed assessment of porosity and pore size is essential when evaluating the performance of new heterogeneous catalysts. Herein, we report the synthesis and characterization of a series of novel microporous Tröger’s base polymers and copolymers (TB-PIMs) with tunable pore size. The basicity of TB sites is exploited to catalyze the Knoevenagel condensation of benzaldehydes and malononitrile, and the dimension of the pores can be systematically adjusted with an appropriate selection of monomers and comonomers. The tunability of the pore size provides the enhanced accessibility of the catalytic sites for substrates, which leads to a great improvement in conversions, with the best results achieving completion in only 20 min. In addition, it enables the use of large benzaldehydes, which is prevented when using polymers with very small pores, typical of conventional PIMs. The catalytic reaction is more efficient than the corresponding homogeneous counterpart and is ultimately optimized with the addition of a small amount of a solvent, which facilitates the swelling of the pores and leads to a further improvement in the performance and to a better carbon economy. Molecular dynamic modeling of the copolymers’ structures is employed to describe the swellability of flexible chains, helping the understanding of the improved performance and demonstrating the great potential of these novel materials.

## Introduction

The amazing scientific progress of the last few years has shown how important the design of polymers is, especially when producing new, more efficient, and cheaper materials that can be used for catalytic applications. In fact, catalysis is regularly employed by both academia and industry to improve the rate of reactions,^1−3^ helping to meet the increasing demand for selected fine products and facilitating their scalability.^4,5^ A good example is provided by the need for technological advancements that can improve the production of biodiesel, which is crucial if we want to reduce our dependence on fossil fuels. We cannot currently meet its high demand,^6−8^ and yet it is rapidly increasing and expected to account for 10% of the total world fuel supply by 2035.^3^ Several groups tackled this problem by synthesizing new and more efficient catalysts, such as Bhaumik,^9^ Varma,^10^ and Das,^11^ who reported porous polymers able to synthesize biodiesel at room temperature. Many other environmentally important applications also require the use of efficient catalysts, such as the production of biodegradable plastic,^12^ or the reutilization of CO_2_ to form added valuable chemicals.^13^ The latter is becoming more and more critical, as finding an effective way to reuse the CO_2_ captured during the so-called carbon capture storage and utilization (CCSU) is as imperative as its removal from the environment.^14,15^ In this context, excellent materials are reported by Iglesias,^16^ Coskun^13^ and Yang,^17^ who found successful ways to convert CO_2_ into cyclic carbonates that can be used as solvents, industrial lubricants, or electrolytes for lithium-ion batteries.^18^

The design and synthesis of new catalysts are also essential for general organic synthesis, as shown by the work on the formation of new C–C bonds developed by Yavuz^19^ and Bhaumik,^20^ by the insertion of carbene into N–H bonds reported by Huang^21^ and Sánchez^22^ or the asymmetric synthesis from Kunz.^23^ Most of these reactions can be performed in either the homogeneous or heterogeneous phase^24^ and, while homogeneous catalysts are often associated with faster conversions, a well-known advantage of heterogeneous catalysts is their easier recovery and reutilization.^25,26^ This not only lowers the energy required for recycling but also makes the reactions more environmentally friendly and sustainable.^27^ The best heterogeneous catalysts typically benefit from high porosity that forces the reagents into small cavities and maximizes their contact with active sites (i.e., within the walls of a pore).^28,29^ The simplest way to prepare them relies on anchoring the catalytic sites onto a pre-existing polymeric support,^20,30^ which bears the disadvantage of the possible leaching of the catalytic material into the reaction environment, making their recycling more problematic and costly.^31,32^ To prevent this, the catalytic site can be covalently bonded to the polymer or, even better, be an integral part of the polymer itself. The design and synthesis of such novel porous materials represent a very challenging task, and not many polymers with these important characteristics are reported to date.^33^

Polymers of intrinsic microporosity (PIMs) represent a relatively new class of amorphous materials. Their porosity originates from the inefficient packing of the molecular chains in the solid state, creating voids of nanodimensions that can be exploited for a wide range of applications, including gas separation,^34−37^ gas storage,^38,39^ water treatment,^40,41^ and catalysis.^42−44^ A new class of such polymers, prepared via the formation of the interesting and reactive core known as Tröger’s base (TB-PIMs), was recently introduced.^45,46^ They combine the typical high porosity of PIMs with the presence of two bridged nitrogens, which can be exploited for either neutral^47^ or alkaline^48^ catalysis. In 2014, a series of TB-PIMs was successfully used to perform the Knoevenagel condensation of benzaldehyde and malononitrile, which is a typical protocol to test base-catalyzed reactions.^49^ These materials provided several advantages over other catalysts. They are highly microporous (with Brunauer–Emmett–Teller (BET) surface areas up to ∼1000 m^2^ g^–1^) and completely insoluble, making them ideal for heterogeneous catalysis, and, most importantly, their active catalytic site (the TB core) is an integral part of the polymer backbone. The latter feature massively reduces the leaching problem and guarantees quick recyclability.^50−52^ Furthermore, these TB-PIMs demonstrated faster conversions than their homogeneous counterparts.^49^

**Figure 1:**
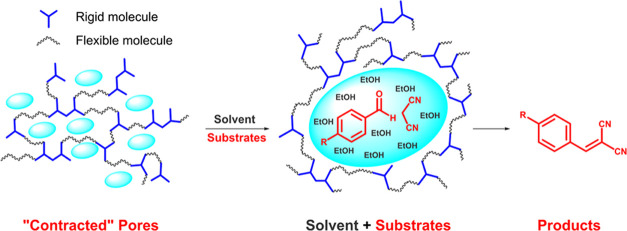
Cartoon illustrating the expansion of “swellable” pores.

A potential problem that arises from the use of these amorphous polymers and therefore limits their broad use as heterogeneous catalysts is that their very high surface areas often lead to the formation of very small pores. This means that they cannot host large substrates, which is a problem also exhibited by other microporous materials such as COFs.^53,54^ To work around this issue, we herein report the synthesis and characterization of a series of novel TB polymers and copolymers composed of very “rigid monomers”, which are typical of highly porous PIMs, combined with slightly more flexible “side arms”. The introduction of the latter leads to a reduction of the overall porosity, as the polymer chains can pack more efficiently, but the enhanced mobility is meant to produce a high degree of “swelling” (especially in the presence of a solvent), in the same fashion as seen in some “breathing MOFs”.^55,56^ This change in morphology grants the accessibility to the pores for larger substrates, which is prevented with common PIMs, and makes the catalytic sites available for a broader variety of compounds (Figure 1). The catalytic performance of the new polymers was tested via the established Knoevenagel condensation, and to prove the superior accessibility of the pores and the role of the solvent, we tested benzaldehydes of different sizes and performed the catalytic reaction in the presence of a solvent. The choice of the different “rigid monomers” and “side arms” was based on the concept of the “polymer genome”.^57,58^ The experimental results were supported by a series of molecular dynamic simulations, which confirmed the crucial effect that the change in pore size and the choice of the solvent have in this kind of catalytic reaction.

## Results and Discussion

### Synthesis and Characterization of TB-PIMs

**Figure 2:**
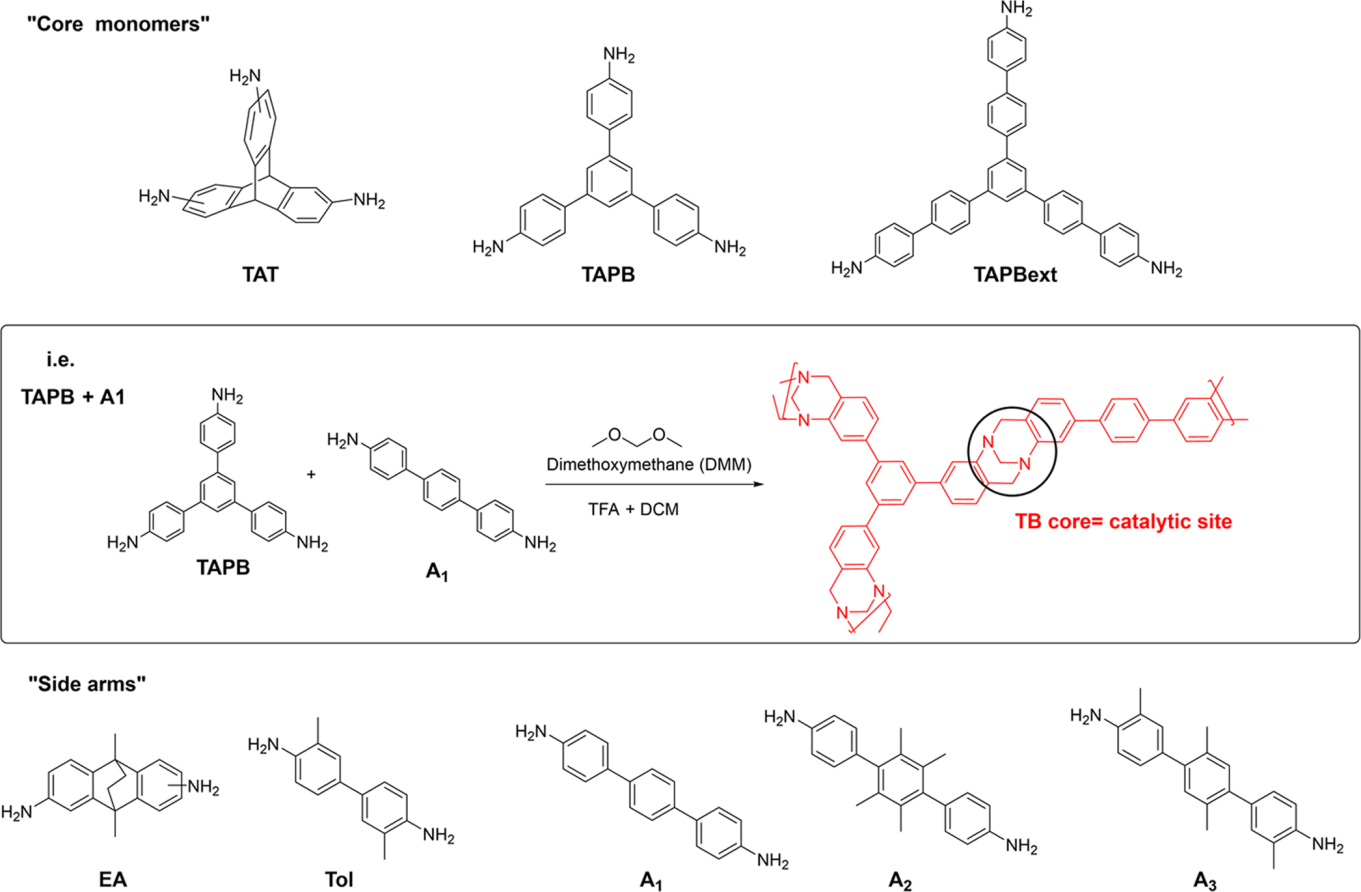
“Cores” and “side arms” monomers used in the synthesis of the novel TB-PIMs.

Fourteen new TB-PIMs, two homopolymers and 12 copolymers (Table S1), were synthesized as shown in Figure 2. The materials were made starting from monomers with different geometries and aromatic moieties of different lengths, so that their structural diversity would enable the tunability of the pore size. To guarantee the necessary rigidity, which typically generates microporosity,^59−62^ we chose tri(amino)triptycene (**TAT**), di(amino)ethanoanthracene (**EA**), tri(amino)phenylbenzene (**TAPB**), and its extended version, herein called **TAPBext**, as all provide “sites of contortion” characteristic of porous PIMs. To increase the flexibility of the polymer chains, and to tune the pore size, we selected a series of dianilines with aromatic moieties that extend the “side arms” around the TB sites. This change aims to produce larger and more swellable pores, and it can be achieved with monomers such as *o*-tolidine (**Tol**), which is commercially available, along with three custom-made extended dianilines that, for simplicity, we herein called **A1**, **A2**, and **A3**. From the structural point of view, **TAT**, **EA**, and **TAPB** were previously employed to perform TB polymerizations,^49,63−65^ whereas **TAPBext**, along with side arms **A1**, **A2**, and **A3** shown in Figure 2, are herein reported for this use for the first time.

**Figure 3:**
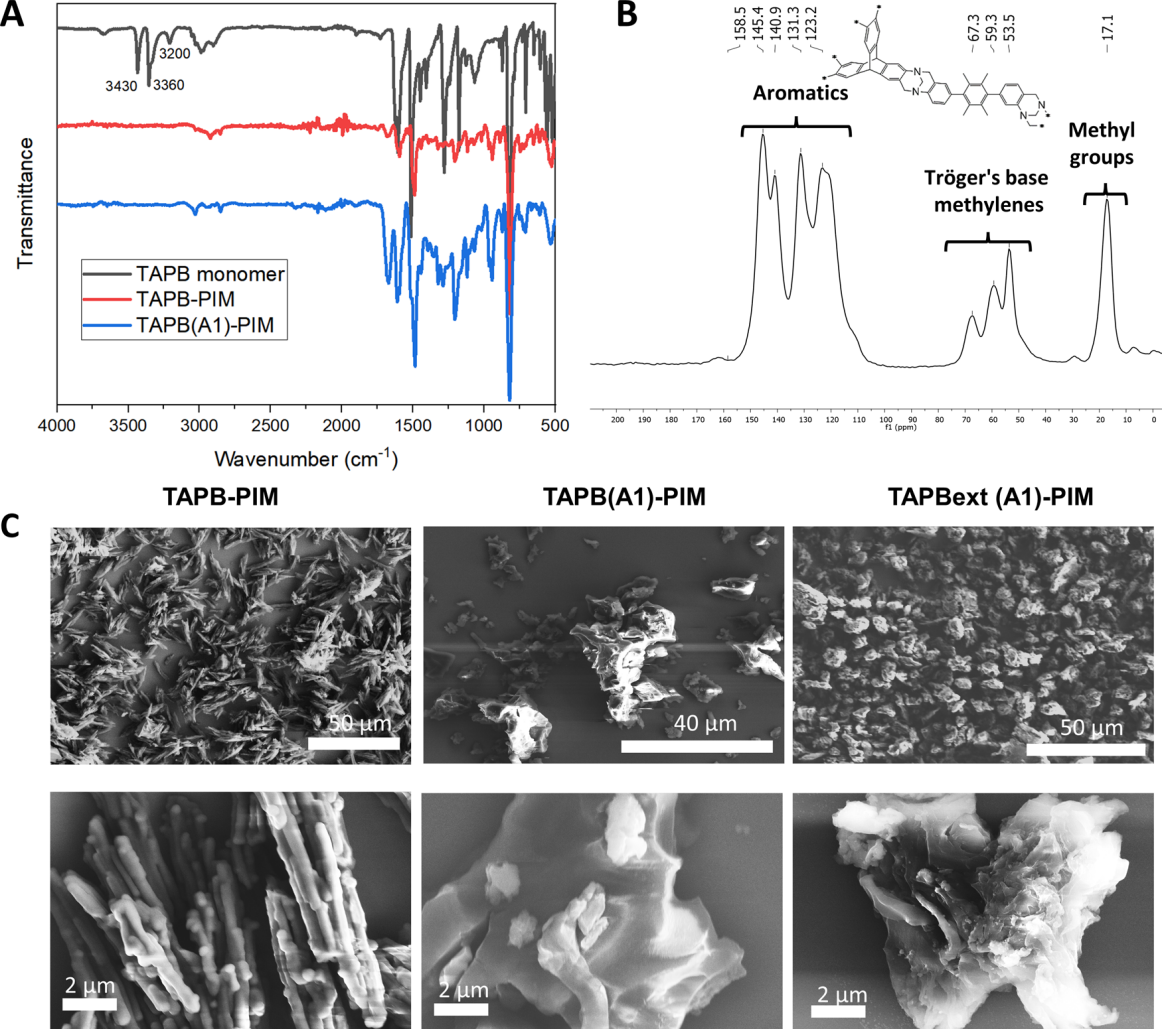
(A) Overlay of the FT-IR spectra of the **TAPB** monomer and the **TAPB-PIM** homopolymer and **TAPB(A1)-PIM** polymers; (B) ^13^C solid-state NMR of **TAT(A2)-PIM**; and (C) scanning electron microscopy (SEM) images of **TAPB** polymers.

All polymers and A–B random copolymers were synthesized according to the known TB polymerization protocol,^49^ which involves using dimethoxymethane, as the methylene precursor needed for the formation of TB bridges, trifluoracetic acid (TFA) as the solvent, and acid media, and small amounts of dichloromethane (DCM), needed to help the initial solubility of the monomers in the reaction environment. To ensure that all of the polymerization sites were engaged, and not many ending groups were left unreacted, we employed stoichiometric amounts of each polymerization site. Therefore, each monomer with three amino moieties was reacted with 1.5 equiv of the monomer that contains only two amine sites.^45,46,49^ The proposed materials were characterized by Fourier transform infrared (FT-IR) and solid-state ^13^C NMR to prove their correct structures and isothermal gas adsorption to assess their porosity. The efficient formation of the TB chains was confirmed by FT-IR (Figure 3A), which shows the disappearance of the characteristic amino peaks at 3430, 3360, and 3200 cm^–1^ from the **TAPB** monomer (Figure 3A). Although it is always possible that not all of the amine sites were converted into TB cores and some ending groups may still be present, high polymerization yields (70–95%) confirm that the reactions produced high-molecular-mass polymers. The same behavior was observed for all of the other synthesized polymers (see Supporting Information (SI)),

Further evidence of the formation of the TB core was supported by solid-state ^13^C crosspolarization magic angle spinning (CP/MAS) NMR (Figure 3B and SI). The relative position of the peaks is perfectly aligned with previously reported TB-PIMs.^46,49^ Signature peaks around 70–50 ppm are attributed to the methylenes of the Tröger’s base bridge, while the area around 120–160 ppm corresponds to aromatic carbons. Aliphatic carbons present in some monomers, such as **EA**, **Tol**, **A2**, and **A3**, appear around 17 ppm. As an inclusive example, Figure 3B shows the spectrum of **TAT(A2)-PIM**. SEM images of the TAPB series show that the more rigid **TAPB-PIM** homopolymer exhibits a regular morphology, forming rodlike aggregates, whereas more flexible **TAPB(A1)-PIM** and **TAPBext-(A1)-PIM** both produce globular and swollen particles, suggesting higher flexibility of their molecular chains (Figure 3C).

Thermogravimetric analysis (TGA) showed the excellent stability of these polymers, which is typical of PIMs, with thermal degradation occurring over 400 °C (Table S1). It also proved very useful to demonstrate the relative reactivity of the different monomers, which is crucial to confirm the correct composition of the copolymers. In fact, since the material is completely insoluble, any solution-based structural characterization (e.g., ^1^H NMR or gel permeation chromatography (GPC)) is not achievable. Elemental analysis was performed in the attempt to verify the composition of the repeated units, but the experimental values proved too distant from the calculated ones. This is common in highly porous materials, as they can easily uptake atmospheric compounds sure as water vapor and other gases, which is likely the cause of the variation of elemental ratios.^66−68^

The successful determination of the composition was proved by TGA, quantifying the mass lost from a thermally labile part of a selected monomer and subtracting it from the weight of the calculated repeated unit. This experiment is feasible if the mass loss from a monomer occurs before the thermal decomposition of the rest of the backbone, and the related percent of this loss can be perfectly isolated from the rest of the curve. We applied this methodology by copolymerizing **TAPB** with ethanoanthracene (**EA**) and monitoring the loss of the ethylene bridge of **EA**. The latter is due to thermal retro Diels–Alder that typically occurs between 250 and 400 °C, whereas the degradation of the rest of the backbone starts at ∼440 °C, as seen with other **EA** containing TB-PIMs.^46,63^ The experiment showed a ∼6% loss from **EA**, which fits perfectly with the proposed A–B structure (Figure S3). Since all of the reported TB polymerizations proved very similar in terms of reactivity and yields, we can safely assume that all copolymers roughly follow this trend.

### Porosity Characterization

**Figure 4:**
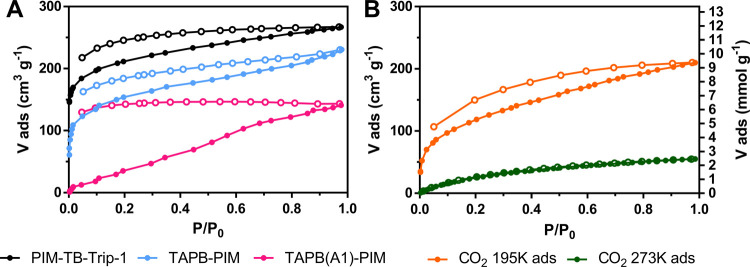
(A) N_2_ adsorption and desorption isotherms measured at 77 K for **PIM-TB-Trip-1**, **TAPB-PIM**, and **TAPB(A1)-PIM**. (B) CO_2_ adsorption and desorption isotherms for **TAPB(A1)-PIM** at 195 and 273 K. Full points represent adsorption, whereas empty ones represent desorption.

Both the previously published **PIM-TB-Trip-1** and the homopolymer **TAPB-PIM** adsorbed a significant amount of N_2_ at low partial pressure (*P*/*P*_0_). Despite the large difference in the BET surface area (SA_BET_ ∼ 1000 and ∼500 m^2^ g^–1^, respectively), both gas adsorption isotherms are consistent with microporous structures (Figure 4A). The more flexible copolymers **TAPB(A1)-PIM** showed much lower adsorption of N_2_ at 77 K, leading to a difficult evaluation of its SA_BET_ with this method. This is attributable to the increased flexibility of the polymer chains that, especially at this extremely low temperature, permits a denser packing and poor interconnectivity of the pores that become less accessible for nitrogen. The result is neither unexpected nor unwanted, since we purposely designed the polymer so that the flexible chains could swell during the catalysis reaction at room temperature, expanding the pores and facilitating access to larger substrates. The swelling of the pores of copolymers is substantiated by the pronounced hysteresis of **TAPB(A1)-PIM**, as shown in Figure 4A. Recent works demonstrate that a marked hysteresis can be, indeed, associated with the swelling of the pores during the adsorption/desorption of condensable probe gases.^69^ The morphology of the chains changes with the increase of the partial pressure and is maintained during desorption.^69,70^ This effect is clearly less pronounced when the material is rigid and most of the pores are very small (∼3.5 Å), such as in archetypal **PIM-TB-Trip-1**.

For a more in-depth understanding of porosity, we performed CO_2_ adsorption at different temperatures (195 and 273 K). In fact, both the higher adsorption temperature and the smaller kinetic diameter of CO_2_ (3.3 vs 3.64 Å of N_2_)^71−73^ seemed more appropriate for assessing the porosity of the more flexible copolymers. A significant amount of CO_2_ was adsorbed by **TAPB(A1)-PIM** at both 195 and 273 K, with a type-I isotherm that is characteristic of microporous materials (Figure 4B).^74^ The BET surface area calculated from CO_2_ adsorption at 195 K gave an estimated surface area of 560 m^2^ g^–1^ that, within the margin of error typical of this calculation, is comparable to the one obtained from the adsorption at 273 K (470 m^2^ g^–1^). Similar measurements were performed on polymers known to produce high SA_BET_ when measured via N_2_ adsorption at 77 K (i.e., **PIM-TB-Trip-1**),^49^ and they showed very similar and consistent results. Considering that the measurement with CO_2_ at 273 K is faster and very useful for the determination of the pore size via nonlocal density functional theory (NLDFT) calculations (Figure S2),^75−77^ we chose this protocol to assess the porosity of all of the polymers and copolymers herein reported (Table S1).

### Catalytic Tests: The Knoevenagel Condensation

**Figure 5:**
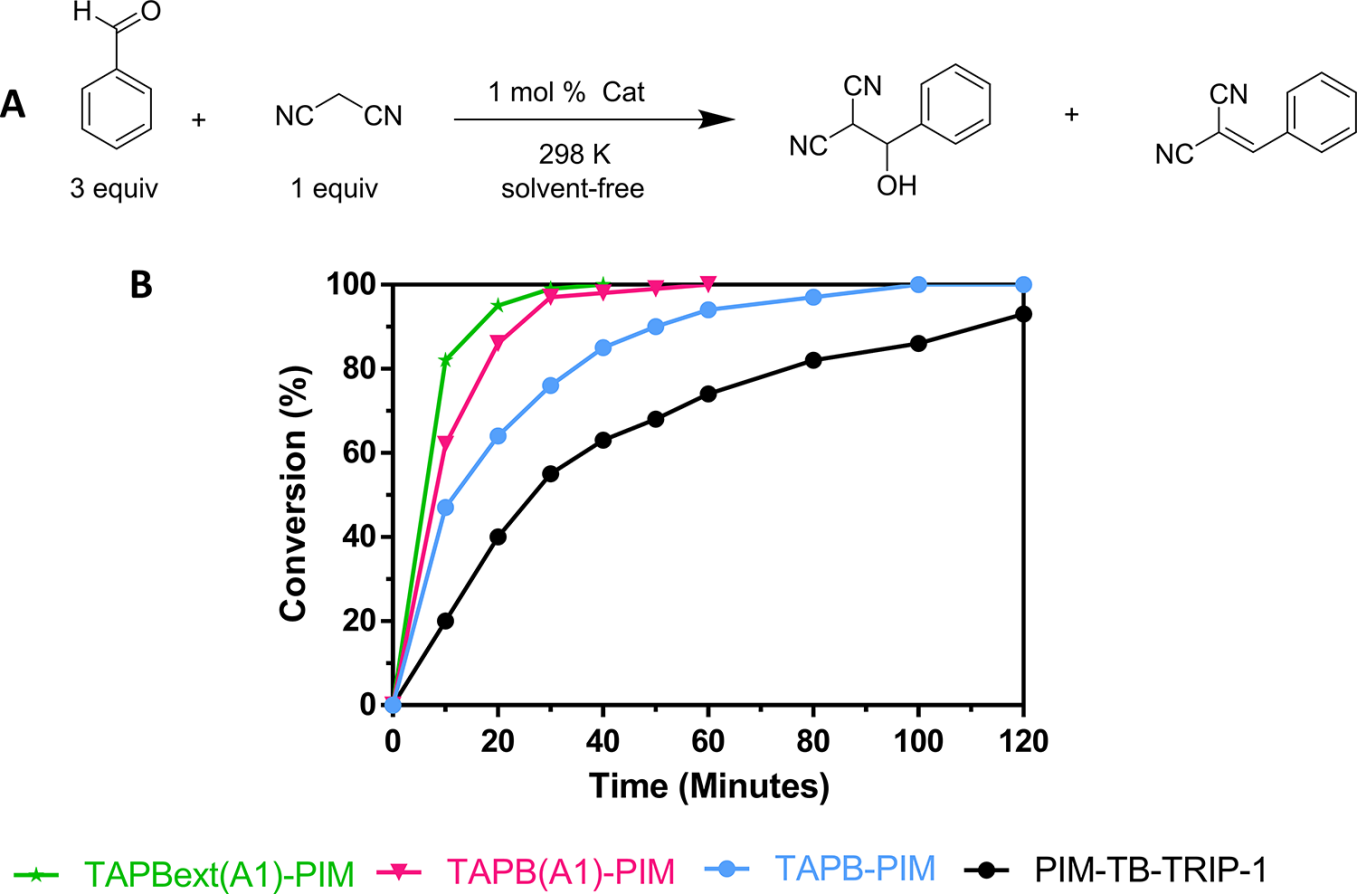
Benzaldehyde versus malononitrile (3:1) under solvent-free conditions. (A) General reaction. (B) Catalytic performance.

The catalytic efficiency of novel TB polymers was tested by monitoring the Knoevenagel condensation of benzaldehyde and malononitrile (Figure 5A). This reaction represents a common procedure used to test the performance of base catalysts, but it is also of broad industrial interest, as it is often utilized to produce pharmaceuticals and fine chemicals.^78−80^ From the analytical point of view, a great advantage is provided by the easy monitoring of its conversion rates, which can be simply assessed by ^1^H NMR by sampling with defined frequency, and plotting the results against time (Figures 5B and S1). This is in stark contrast with other techniques, such as gas chromatography–mass spectrometry (GC–MS), which require internal standards and, often, poorly reproducible calibration curves.

To guarantee a fair term of comparison between the performance of novel **TB-PIMs** and the already published **PIM-TB-Trip-1**, the original procedure was initially followed by reacting 3 equiv of benzaldehyde and 1 equiv of malononitrile in “solvent-free” conditions at 298 K (the two extra equivalents of benzaldehyde act as a solvent).^49^ All polymers performed extremely well with only 1 mol % catalyst loading (also considering the number of TB sites per repeated unit) and achieving complete conversion in less than 2 h (over 90% during the first hour, Table 1).

**Table 1:** Novel Polymer Catalytic Conversion of Benzaldehyde and Malononitrile Knoevenagel Condensation and Comparison with Reported Works^a^

		conversion (%) at *x* time (min)^b^						
entry	catalyst	20	30	60	80	120	TON^c^	TOF^d^
1	**PIM-TB-Trip-1**(49)	45	55	74	80	93	45	2.3
2	**TAPB-PIM**	64	76	94	97		64	3.2
3	**TAPBext-PIM**	78	87	98			78	3.9
4	**TAPB(Tol)-PIM**	63	75	92	96		63	3.2
5	**TAPB(A1)-PIM**	86	97				86	4.3
6	**TAPB(A2)-PIM**	64	74	92	98		64	3.2
7	**TAPB(A3)-PIM**	76	87	98			76	3.8
8	**TAPB(TAT)-PIM**	69	82	94	97		69	3.5
9	**TAT(Tol)-PIM**	54	70	90	94	98	54	2.7
10	**TAT(A1)-PIM**	63	78	95	97		63	3.2
11	**TAT(A2)-PIM**	75	85	98			75	3.8
12	**TAT(A3)-PIM**	79	85	96	98		79	4
13	**TAPBext(Tol)-PIM**	83	89	96			83	4.2
14	**TAPBext(A1)-PIM**	95	100				95	4.8
15^e^	no catalyst				30			
16^f^	TB homogeneous	25	35	54	65	85	25	1.3

comparison with other works^g^						
catalyst	metal	mol (%)	solvent	temperature^h^ (°C)	conversion (%)	time (min)
PCC-2a^81^	no	0.17	ethanol	25^a^	88	240
MPU^82^	no	5	THF	50	98	840
IPP-1^83^	no	1	water	25	98	180
TBP^52^	no	10	solvent free	25	98	1440
HMP-TAPA^84^	no	1	H_2_O + 1,4-dioxane	25	99	180
S-HPDVB + PBM-1^85^	no	2.5	toluene + water	80	99	60
PCPs^86^	yes	3	ethanol	25	99	180
1-Zn^87^	yes	0.7	solvent free	60	99	36
HKUST-1^88^	yes	1	EtOAc	70	100	1500
compound 1^89^	yes	2	THF	50	100	90
[Cu(L1)_4_]^+^BF_4_^–1 ^^90^	yes	5	methanol	70	99	240
ZIF-8^91^	yes	5	toluene	25	100	280

a Reaction conditions: a mixture of benzaldehyde (15 mmol) and malononitrile (5 mmol) and a 1 mol % catalyst were stirred at 25 °C for 2 h.

b Conversion of malononitrile was determined by ^1^H NMR.

c Turnover number at 20 min calculated from the number of moles of malononitrile consumed per mole equivalents of the TB catalyst.

d Turnover frequency calculated from turnover number per minute.

e Control reaction without a catalyst gave 30% conversion after 2 h.

f Homogeneous reaction.

g All of the literature reactions are reported using benzaldehyde and malononitrile.

h 25 °C was assumed when the paper reported room temperature.

Despite the lower BET surface area, all of the new polymers outperformed the original **PIM-TB-Trip-1** (Figure 5B). This improvement can be ascribed to the higher flexibility of the **TAPB** chains (Table 1, entry 2). This creates the desired more accessible environment for the two reagents compared to the very small pores of **PIM-TB-Trip-1** (entry 1) but still guarantees close contact between reagents and active sites within the pores. An inverse correlation seems to exist between the conversion rates and the pore size distribution of the polymers, and it can be connected with the easier accessibility of the pores for the substrates. In fact, a lower concentration of ultra-micropores centered at ∼3.5 Å, combined with a higher proportion of larger pores, results in a dramatic improvement of the catalytic performance. Direct evidence is provided by the analysis of Figure S2, as more flexible **TAPBext(A1)-PIM** and **TAPB-PIM** show a lower concentration of ultra-micropores, and yet they produce much better conversion rates compared to more microporous **PIM-TB-Trip-1**. Although the pore size analysis calculated via NLDFT from CO_2_ adsorption is known to deliver mainly qualitative results,^75^ its use for quantitative analysis is feasible when comparing polymers of the same families and morphology.

It is plausible then to associate the reduction of the ultra-microporosity with an increase in the accessibility of the internal TB cores, which maximizes the activity of the polymer while maintaining a confinement effect that is essential in heterogeneous catalysis. The most flexible polymer of the series **TAPBext(A1)-PIM** (Table 1, entry 14) achieved completion in 30 min, with an impressive conversion of 95% in only 20 min. The excellent performance in solvent-free conditions proved that these novel polymers are, indeed, very competitive with state-of-the-art basic catalysts. Table 1 compares the rates obtained with our materials with other very efficient catalysts utilized for Knoevenagel condensations. The conversions shown by our polymers and copolymers are indeed remarkable, especially considering that some of the best results reported in similar works were achieved either at higher temperatures or higher catalyst loading (or both, in some cases), whereas our system was always kept at room temperature, with only a 1% molar loading and, of course, without the need of a metal.

### Effect of a Solvent on the Catalytic Performance

Six representative polymers with improved efficiency were selected to assess the potential effect that a solvent has on the catalytic performance (Table S2). The choice was made according to a combination of BET surface areas, pore size distribution, and initial catalytic activity in “solvent-free” conditions. It was expected that the presence of the solvent, in addition to enhancing the solubility of starting materials and products of the reaction, would also induce the swelling of the polymer chains, further facilitating the access of the reagents to the active sites. This is extremely helpful considering that the products are solid, and their quick formation creates a sludge that hampers the stirring of the mixture and reduces the kinetics of the conversion. The choice of the most suitable solvents fell on DCM and ethanol, which were used in the same v/v ratio as benzaldehyde. The first was selected because it enhances the solubility of both starting materials and products. The second is very attractive for two reasons: the better solvation effect that a protic solvent typically has on charged intermediates, and because it is environmentally friendly, especially compared to halogenated solvents.^92^ The positive effect of ethanol in Knoevenagel condensation has been observed in previous works,^92−94^ and it can be explained by the assumption that a polar solvent helps in stabilizing the charged transition-state complex of the reaction. This contribution overcomes the partly detrimental dilution effect shown in some instances using DCM.

**Figure 6:**
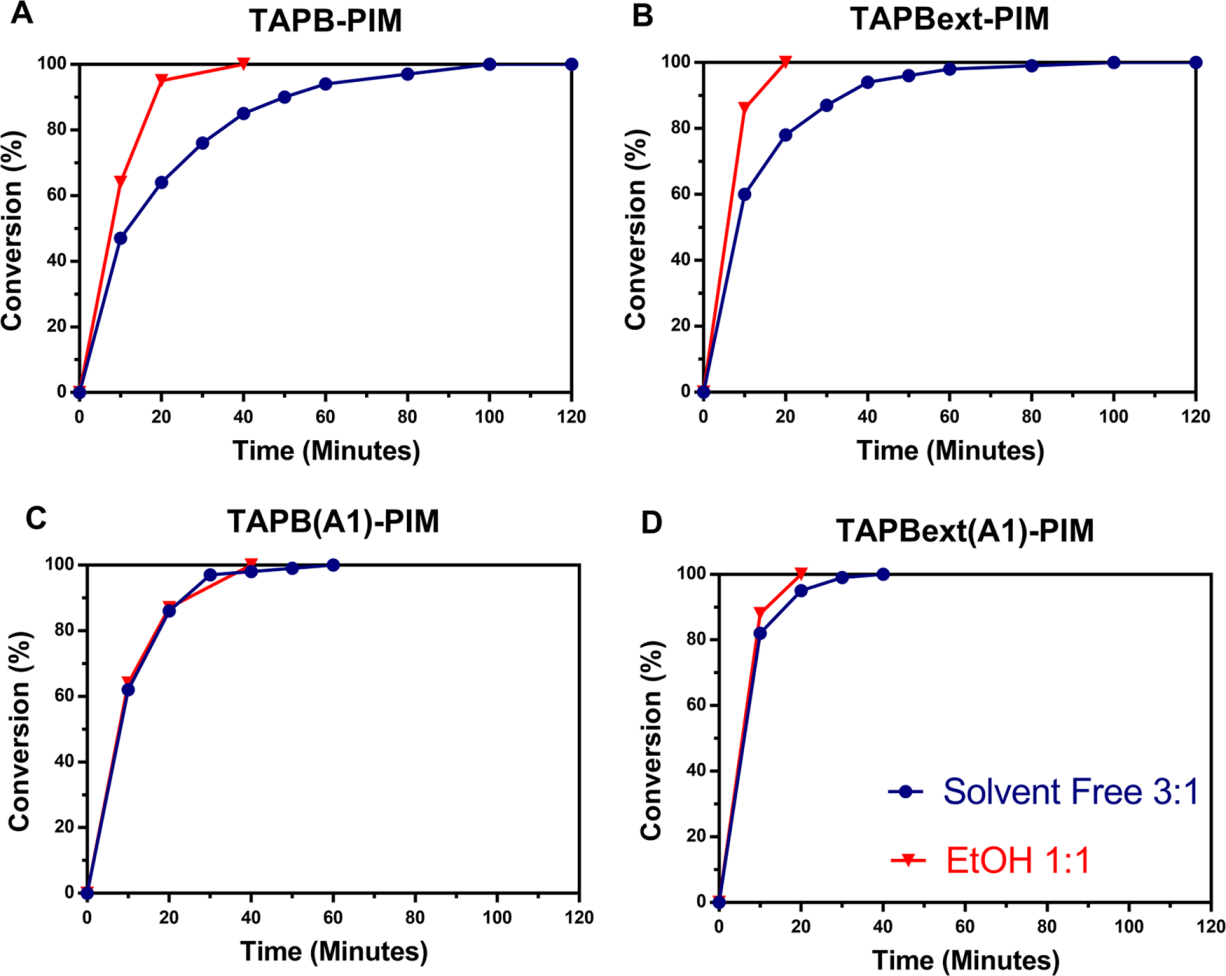
Benzaldehyde versus malononitrile (3:1): the difference under solvent-free and ethanol conditions for (A) **TAPB-PIM**, (B) **TAPBext-PIM**, (C) **TAPB(A1)-PIM**, and (D) **TAPBext(A1)-PIM**.

Initial experiments revealed that DCM does not often have a significant effect on the conversion rates (Table S2), suggesting that it mainly dilutes the concentration of the catalytic sites and their contact with the catalytic sites. Ethanol proved to be an excellent choice, as it improved the rates with all of the tested polymers. Considering the largely superior conversions and that, in solvent-free conditions, we are “wasting” two equivalents of benzaldehyde, we also decided to change the ratio of the two reactants from 3:1 to 1:1. This massively improves the carbon economy and allows for the easier purification of the products and faster recycling of the catalysts. It is worth noting that when we use less benzaldehyde, the presence of ethanol leads to an improvement in the conversion rates for more rigid **TAPB-PIM** and **TAPBext-PIM** (Figure 6A–D), but it does not affect the rates of more flexible **TAPB(A1)-PIM** and **TAPBext(A1)-PIM**. This is likely due to a synergistic effect of the swelling of the chains and the solvation properties of ethanol. This produces a more evident effect on the more rigid polymers, whereas the pores of the more flexible ones are already too large to be affected. On the other hand, the latter show great potential that these polymers have in boosting the carbon economy of the reaction. In fact, when small substrates are employed in combination with a solvent, their catalytic performance with a 1:1 ratio is remarkably similar to the original ratio of 3:1. This means that with our system, we can obtain excellent yields in a very short time (up to 100% in 20 min), without producing waste and without the need of difficult separations and purifications of the products.

### Catalysis with Larger Benzaldehydes

**Figure 7:**
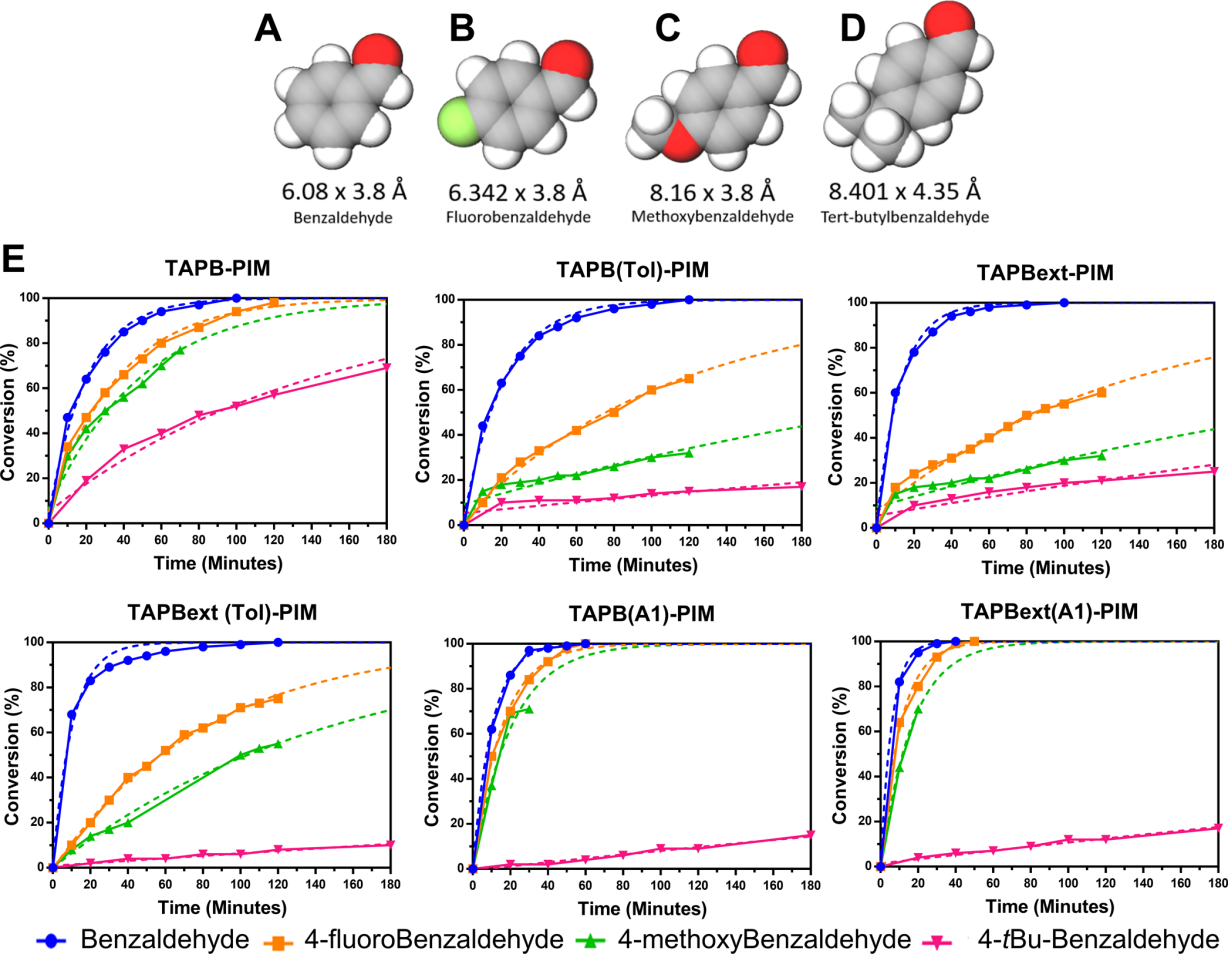
Model structures of (A) benzaldehyde, (B) *para*-fluoro-benzaldehyde, (C) *para*-methoxy-benzaldehyde, and (D) *tert*-butylbenzaldehyde. (E) Conversions of the Knoevenagel condensation using benzaldehyde and malononitrile (3:1) under solvent-free conditions.

Motivated by the performance improvement of the reaction rates with simple benzaldehyde, we decided to extend our study by testing the accessibility of the larger pores with bulkier reagents (Figure 7A–D). The initial choice fell on 4-fluoro, 4-methoxy, and 4-*tert-*butylbenzaldehyde, especially as they are liquid at room temperature, so their solubility is not a concern when we tested them in solvent-free conditions. To have a fair comparison with the previous experiments, all of the polymers were initially reacted in the typical 3:1 ratio. We anticipated that all these new reagents must have a slightly different reactivity compared to the original benzaldehyde. In fact, the electron-withdrawing effect of fluoride would enhance the electrophilicity of carbonyl, improving the rate of reaction compared to the methoxy and *tert*-butyl groups, which are electron-donating instead. This trend was confirmed for all our selected polymers, as shown in Figure 7E and Table S3. All reagents were also tested in homogeneous conditions, using the simplest Tröger’s base molecule (2,8-dimethyl-6*H*,12*H*-5,11-methanodibenzo[*b*,*f*][1,5]diazocine), which was also employed to test the polymers published in 2014.^49^ All of the homogeneous results proved to be less efficient than our best copolymers (Table S7). The catalytic activity with the larger substrates decreased with the increase of the size of benzaldehyde (Figure 7E). This does not come as a surprise, especially in solvent-free conditions, as the bulkier reagents are likely to have more difficulty entering the pores and therefore have relatively limited access to the TB sites. It is worth noting, though, that the more flexible polymers performed slightly better than the more rigid ones, which begins to confirm the hypothesis that the swelling effect plays a crucial role.

The rates obtained using 4-fluorobenzaldehyde and 4-methoxybenzaldehyde proved somewhat similar, although the products solidified very quickly, hampering the stirring of the mixture and slowing down the rate of reaction. Because of that, ^1^H NMRs were often run before the reaction could complete. This does not imply that the size and accessibility of the pores are not adequate, but simply that the reaction needs extra energy to keep reactants and products in solution (i.e., higher temperature, longer time, or different solvents). Since we aimed to keep the same room temperature conditions, we decided to extrapolate the data for these results, following the slope of the curve rather than increasing the temperature or adding more solvent (hence the dotted lines in Figure 7E). An almost perfect overlay between the extrapolated and the actual results was achieved with 4-*tert*-butylbenzaldehyde (dotted and full pink lines in Figure 7E), showing that our projections for the more insoluble products are reasonable. For the more flexible polymers, **TAPB(A1)-PIM** and **TAPBext(A1)-PIM**, the differences in reactivity among benzaldehyde, 4-fluorobenzaldehyde, and 4-methoxybenzaldehyde are almost negligible, showing that the flexibility of these polymers makes the pores accessible for all these substrates. However, all of the tested polymers performed poorly using 4-*tert*-butylbenzaldehyde under solvent-free conditions, with the exception of slightly more rigid **TAPB-PIM**, which shows 69% conversion after 180 min (Figure 7E and Table S3). We believe that this is due to its higher ratio of nitrogen/carbon per repeated unit compared to the other two polymers, which allows some catalysis to happen on the surface of the polymer without the need to penetrate the inner pores.

### Effect of the Solvent with Larger Benzaldehydes

To confirm the improved accessibility of the pores, the reactions with larger benzaldehydes were also performed in the optimized conditions, using a 1:1 ratio and ethanol or DCM as a solvent. All of the results are displayed in Table S4. We found that the reactivity trend is similar to the one displayed in solvent-free conditions, as larger benzaldehydes produced lower conversions, confirming the selectivity of these polymers based on the pore size. All of the final conversions, however, proved to be much better in the presence of a solvent. This demonstrates that the combination of better solubility/solvation of the substrates and the higher swellability of the polymer chains is essential to increase the catalysis performance.

The most interesting results were obtained with the reaction with bulky 4-*tert*-butylbenzaldehyde, which proved to be very challenging in solvent-free conditions, but that worked extremely well in the presence of either ethanol or DCM.

**Figure 8:**
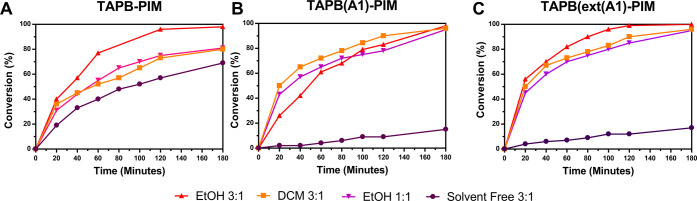
4-*tert*-Butylbenzaldehyde versus malononitrile: the difference under solvent-free, DCM, and ethanol conditions for (A) **TAPB-PIM**, (B) **TAPB(A1)-PIM**, and (C) **TAPBext(A1)-PIM**.

**Figure 9:**
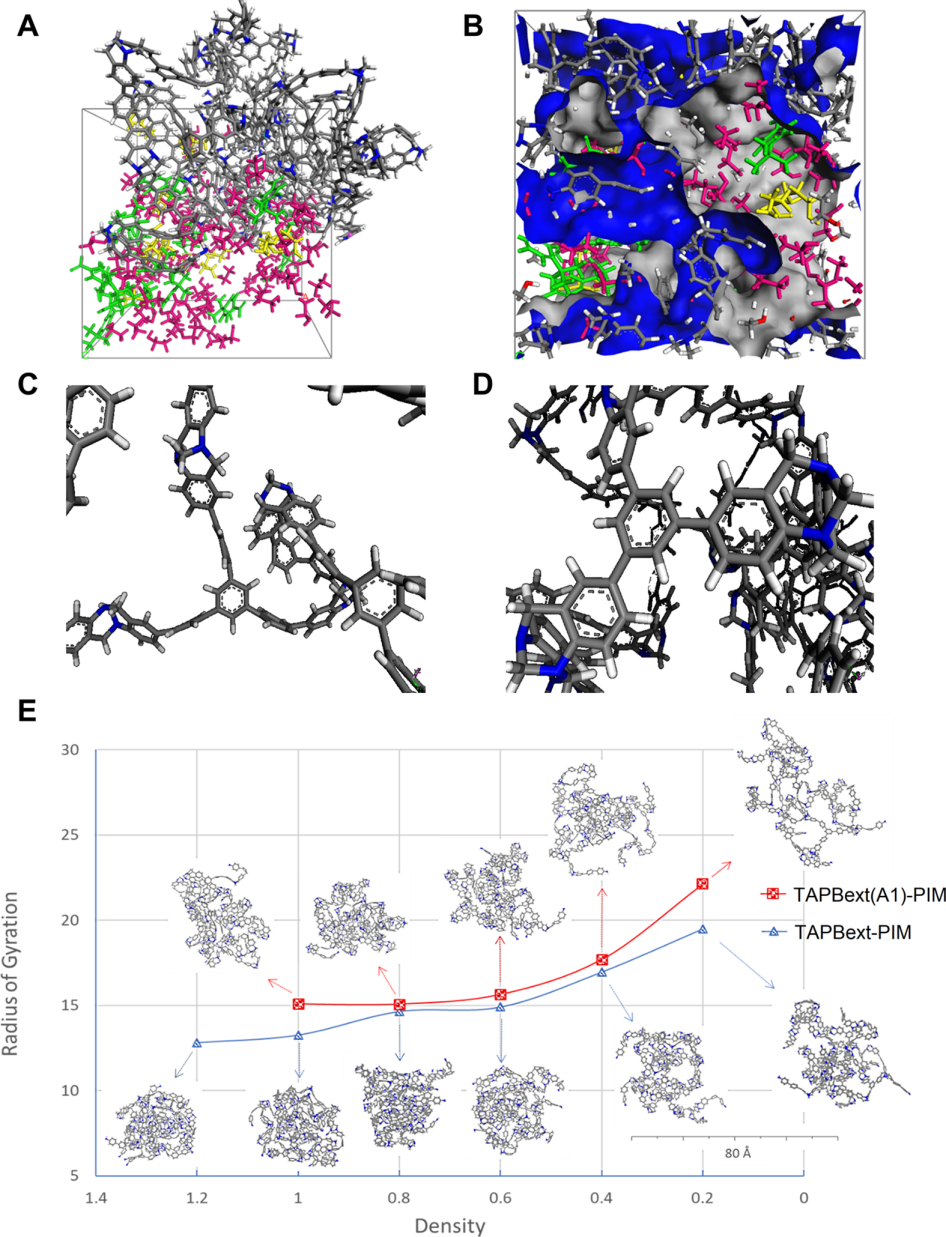
(A) Simulation image of the **TAPBext(A1)-PIM** model at *d* = 1 g cm^–3^, with solvent molecules in the mole ratio: ethanol = 6.8 (pink color), *t*-*Bu-*benzaldehyde = 1 (green color), and malononitrile = 1 (yellow color). (B) Void space ascribed by the van der Waals isosurface. (C) **TAPBext(A1)-PIM** and (D) **TAPB(A1)-PIM** models (blue for nitrogen, white for hydrogen, and gray for carbon atoms). (E) The radius of gyration as a function of the percentage of swelling for the two polymers **TAPBext(A1)-PIM** and **TAPB(A1)-PIM**.

Figure 8A–C and Table S5 show that the rates are greatly improved when a solvent was used with the more flexible polymers, matching or surpassing more rigid **TAPB-PIM**. Although the catalysis rates can be improved by the enhanced solvation effect that ethanol may have on the intermediates, or the better solubilization of the starting materials in DCM, we observed that this is valid for all of the tested polymers, so we consider this effect as normalized. Our conclusion, then, is that the most significant improvement is attributable to the increased swelling and the larger pores. A supplementary assessment of the swelling of **TAPB(A1)-PIM** and **TAPBext(A1)-PIM** copolymers was observed at a molecular level starting from the calculated densities of the anhydrous materials (ρ_TAPBext(A1)-PIM_ ∼ 1 g cm^–3^, ρ_TAPB(A1)-PIM_ ∼ 1.2 g cm^–3^). The copolymers were cross-linked to simulate intricate networks that occur in real structures, all starting from a semi-reacted state of the monomers (i.e., small seed structures). These seeds were formed by a central trifunctional monomer, surrounded by the first “shell” of bifunctional monomers and a second “shell” of trifunctional monomers, to provide the structure of an ideal random A–B copolymer. The subsequent hydrated models were built by adding solvent molecules and the reagents to generate various degrees of swelling (Figure 9A–E). We chose 4-*tert*-butylbenzaldehyde and malononitrile (1:1) as the most representative example. The trends in the radii of gyration (*R*_g_), which measures the compactness of the polymeric chains, indicated that the swelling increases with each addition of the solvent (Tables S9 and S10). The same polymers tend to have a wider distribution of the radius of gyration in the mixture when ethanol is added compared with the mixture where we include only the reactants. In addition, we found that in the same solvent, more flexible **TAPBext(A1)-PIM** swells more than slightly less flexible **TAPB(A1)-PIM**. Figure 9E confirms that the *R*_g_ of **TAPBext(A1)-PIM** is always larger than that of **TAPB(A1)-PIM**, and at a higher degree of swelling, this effect is even more evident. This study suggests that more flexible **TAPBext(A1)-PIM** tends to be relatively unfolded and more swollen, as indicated by the three-dimensional structures. This is most likely due to the higher number of aromatic moieties present in the molecular structure, and their increased possibility of free rotation. More details on the procedure of the polymeric box creation and cross-linking are given in Section S7 of the SI.

### Catalysis with Even Larger Benzaldehydes

**Figure 10:**
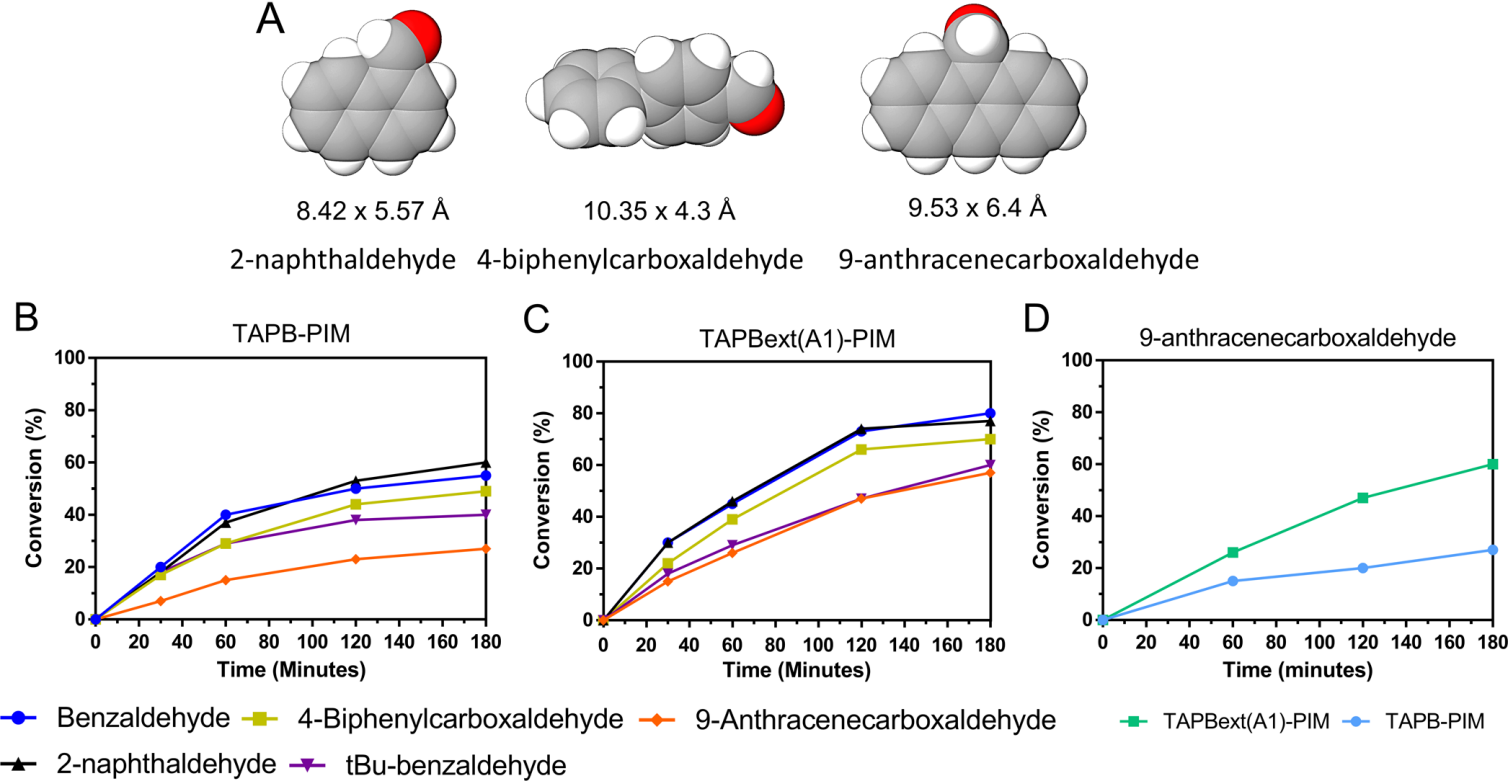
(A) MolView representation and sizes of the larger benzaldehydes. (B) **TAPB-PIM** and (C) **TAPBext(A1)-PIM** with different aldehydes (aldehyde × malononitrile 1:1 in DCM). (D) 9-Anthracenecarboxaldehyde versus malononitrile (1:1 in DCM): the difference using **TAPB(A1)-PIM** and **TAPBext(A1)-PIM**.

Because of the encouraging results with bulky 4-*tert*-butylbenzaldehyde, we extended the study to the even bulkier 2-naphthaldehyde, biphenyl-4-carboxaldehyde, and 9-anthracenecarboxaldehyde (Figure 10A and Table S6), so that we could further elaborate on the size selectivity of these new materials. We focused this test on the two extremes in terms of polymer pore size: the most rigid homopolymer of the new set, **TAPB-PIM**, and the most flexible copolymer, **TAPBext(A1)-PIM**. In this case, the experiments could only be run using DCM as a solvent, as these bulkier reagents are solid at room temperature and completely insoluble in ethanol. While this reduces the “greenness” of the reaction, it still helps us to evaluate the swelling effect with these larger substrates.

Figure 10B shows that the catalytic activity of the most flexible polymer, **TAPBext(A1)-PIM** (Figure 10C), again decreases with the increase of the size of aldehyde, with the best results following the order: benzaldehyde (6.08 × 3.80 Å^2^) > 2-naphthaldehyde (8.42 × 5.57 Å^2^) > 4-biphenylcarboxaldehyde (10.35 × 4.3 Å^2^) > 9-anthracenecarboxaldehyde (9.53 × 6.4 Å^2^) and 4-*tert*-butylbenzaldehyde (8.40 × 4.35 Å^2^). However, when focusing our analysis only on the largest substrate, 9-anthracenecarboxaldehyde, we can see that flexible **TAPBext(A1)-PIM** achieved 60% of conversion in 3 h (Figure 10D), while rigid **TAPB-PIM** reached only ∼30%, in the same exact conditions. We believe that the considerable difference in the performance of the extended PIMs, either with or without the presence of a solvent, proves that the increased pore size and the enhanced swellability of the molecular chains facilitate the accessibility of the substrates into the cavities. This is certainly crucial to improving the conversion rates and establishing the great catalytic potential of these novel PIMs.

### Recyclability Studies

**TAPBext(A1)-PIM** and 4-*tert*-butylbenzaldehyde were selected to evaluate the recyclability and reusability of these new PIMs. The tests were run using a 1:1 ratio of the aldehyde and malononitrile and using ethanol as a solvent, as we concluded that they represented the optimized conditions. The catalyst could be readily recovered from the reaction suspension by simple filtration, washed with aqueous ammonia to remove protons that the TB sites may take (even from CO_2_ and moisture from the environment, as a testament of the basicity of these polymers), refluxed in different solvents, dried, and reused without any apparent loss in the product yield. Figure S4 shows that the high activity remains unaltered during at least seven subsequent cycles (around 92% in the last run), which confirms that the goal to produce highly active, fully recyclable, and thus more sustainable catalysts is reached. This consistency in reactivity after a simple washing also demonstrates the robustness of these materials, paving the way to their exploitation on a larger scale.

## Conclusions

We successfully synthesized a series of novel Tröger’s base polymers of intrinsic microporosity (TB-PIMs) that were used as efficient heterogeneous catalysts for the Knoevenagel condensation. All of the new materials contained more swellable and accessible pores so that they could host larger substrates compared to polymers with very small pore sizes. The enhanced swellability of the reported polymers and copolymers was confirmed by the excellent conversions obtained by these catalysts in the presence of a small amount of the solvent (ethanol or DCM) compared to the same reactions conducted in solvent-free conditions. The catalytic conditions were thoroughly optimized, starting from the typical excess of one of the reagents (usually benzaldehyde/malononitrile (3:1)), which is needed to have a fair comparison with the literature results, to stoichiometric amounts (1:1). The latter showed an improvement of both the rate of reaction and the carbon economy, as all starting materials are consumed to form products. The effect of the solvent was also studied, finding that it induces the swelling of the molecular chains, which can then host larger substrates. Molecular dynamic studies helped us explain that the swelling effect is due to a combination of the larger pore size and the effect of the solvent, elucidating them from the study of the structure–relationship point of view of two copolymers. We can embody the enhancement of the catalytic performance by analyzing the excellent results obtained with the Knoevenagel condensation of bulky 4-*tert*-butylbenzaldehyde and malononitrile, which was helped by a small amount of ethanol. We concluded that while the solvent may have a general positive effect on increasing the conversion rate by helping the solvation of the intermediates, this effect is the same for all of the employed aldehydes. This means that any further improvement is ascribed to the enhanced swelling of the pores and the polymer design.

These results were further validated using even bulkier substrates, confirming that the appropriate design of these copolymers helped in enhancing the general activity of PIMs and that we can “play” with chemistry to tailor the catalytic performance of these amorphous materials.

## Supporting Information Available

Synthesis and characterization of monomers and polymers; tables of catalysis results; various figures; solid-state ^13^C NMRs; and computational models’ description (PDF)
