# Supplementary material for: Tröger’s Base Network Polymers of Intrinsic Microporosity (TB-PIMs) with Tunable Pore Size for Heterogeneous Catalysis
Source: J Am Chem Soc. 2022 Aug 16;144(34):15581–94. doi: 10.1021/jacs.2c04739 (PMC9437925; doi:10.1021/jacs.2c04739)
Supplement: Supplementary file 1 — ja2c04739_si_001.pdf [file ja2c04739_si_001.pdf]

# Electronic supporting information (ESI) for:

# Tröger's Base Network Polymers of Intrinsic Microporosity (TB-PIMs) with Tunable Pore Size for Heterogeneous Catalysis

Ariana R. Antonangelo, [‡a] Natasha Hawkins, [‡a] Elena Tocci, [b] Chiara Muzzi, [b] Alessio Fuoco [b] and Mariolino Carta [a*]

[a] *Department of Chemistry, Faculty of Science and Engineering, Swansea University, Grove Building, Singleton Park, Swansea, SA2 8PP, UK.*

[b] *Institute on Membrane Technology, National Research Council of Italy (CNR-ITM), via P. Bucci 17/C, Rende (CS), 87036, Italy*

## Table of Contents

1. GENERAL METHODS AND EQUIPMENT ........ 2
2. SYNTHESIS OF MONOMERS ........ 2
3. SYNTHESIS OF POLYMERS AND CO-POLYMERS ........ 10
4. GENERAL CATALYSIS TEST: ........ 16
5. TABLES ........ 17
6. VARIOUS FIGURES ........ 22
7. $^{13}$C SOLID STATE NMR ........ 24
8. COMPUTATIONAL MODELS AND METHODS ........ 31
9. REFERENCES ........ 35

## 1. General methods and equipment

Commercially available reagents and gases were used without further purification. All reactions using air/moisture sensitive reagents were performed in oven-dried or flame-dried apparatus, under a nitrogen atmosphere. TLC analysis refers to analytical thin layer chromatography, using aluminium-backed plates coated with Merck Kieselgel 60 GF254. Product spots were viewed either by the quenching of UV fluorescence, or by staining with a solution of Cerium Sulfate in aqueous $H_2SO_4$. Melting points were recorded using a Cole-Parmer Stuart™ Digital Melting Point Apparatus and are uncorrected. Low-temperature $N_2$ (77 K) and $CO_2$ (273 K) adsorption/desorption measurements of PIM powders were made using a Quantachrome Nova-e. Samples were degassed for 800 min at 80 °C under high vacuum prior to analysis. The data were analysed with the software provided with the instrument. NLDFT and H-K analysis were performed to calculate the pore size distribution and volume, considering a carbon equilibrium transition kernel at 273 K based on a slit-pore model; the kernel is based on a common, one centre, Lennard-Jones model. TGAs were performed using the device Thermal Analysis SDT Q600 at a heating rate of 10 °C/min from 30 to 1000 °C. $^{1}$H NMR spectra were recorded in the solvent stated using an Avance Bruker DPX 500 (500 MHz) instruments, with $^{13}$C NMR spectra recorded at 125 MHz.Solid-state $^{13}$C NMR spectra were recorded using a Bruker Avance III spectrometer equipped with a wide-bore 9.4 T magnet (Larmor frequencies of 100.9 MHz for $^{13}$C). Samples were packed into standard zirconia rotors with 4 mm outer diameter and rotated at a magic angle spinning (MAS) rate of 12.5 kHz. Spectra were recorded with cross polarisation (CP) from 1H using a contact pulse (ramped for 1H) of 1.5 ms. High-power ($\nu_1 \approx$ 100 kHz) TPPM-15 decoupling of 1H was applied during acquisition to improve resolution. Signal averaging was carried out for 6144 transients with a recycle interval of 2 s. Chemical shifts are reported in ppm relative to $(CH_3)_4Si$ (TMS) using the $CH_3$ signal of L-alanine (δ = 20.5 ppm) as a secondary solid reference. SEM images were recorded with a Hitachi S-4800 field emission (~1 nm resolution).

## 2. Synthesis of monomers

### 1,3,5- Tris(aminophenyl)benzene[1]

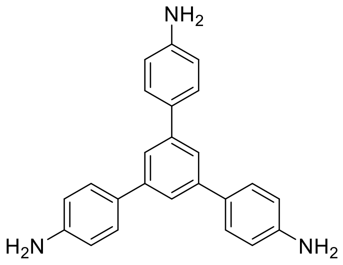


1,3.5-tribromobenzene (2.0 g, 1 equiv., 6.4 mmol) and 4-aminophenylboronic pinacolate (4.48 g, 3.2 equiv., 20.5 mmol) were dissolved in a mixture of THF: toluene (100 mL, 50:50 v/v), followed by addition of NaOH (3.84 g, 15 equiv., 318 mmol). The resulting mixture was degassed for 15 min by a flow of nitrogen, and $Pd(PPh_3)_2Cl_2$ (0.35 g, 0.08 equiv., 0.5 mmol) was added. The resulting mixture was degassed again for 10 min by a flow of nitrogen and was heated to 90 ºC for 20 h under nitrogen atmosphere. The reaction mixture was cooled to room temperature and

the solvents were removed under reduced pressure. The remaining crude product was solubilized in hot ethyl acetate and the mixture was hot filtrated over celite, which was washed with hot ethyl acetate many times. The solvent was removed under reduced pressure and the obtained solid was washed with hot methanol and filtrated to yield a pale-yellow powder (1.3 g, 58% yield). $^1$H NMR(500 MHz, DMSO-d6): δ= 7.49 (s, 3H), 7.48 – 7.47 (d, 6H), 6.68 – 6.66 (d, 6H), 5.261(s, 6H). $^{13}$C NMR (100 MHz, DMSO-d6): δ = 148.70, 141.97, 128.42, 127.57, 120.84, 114.67.

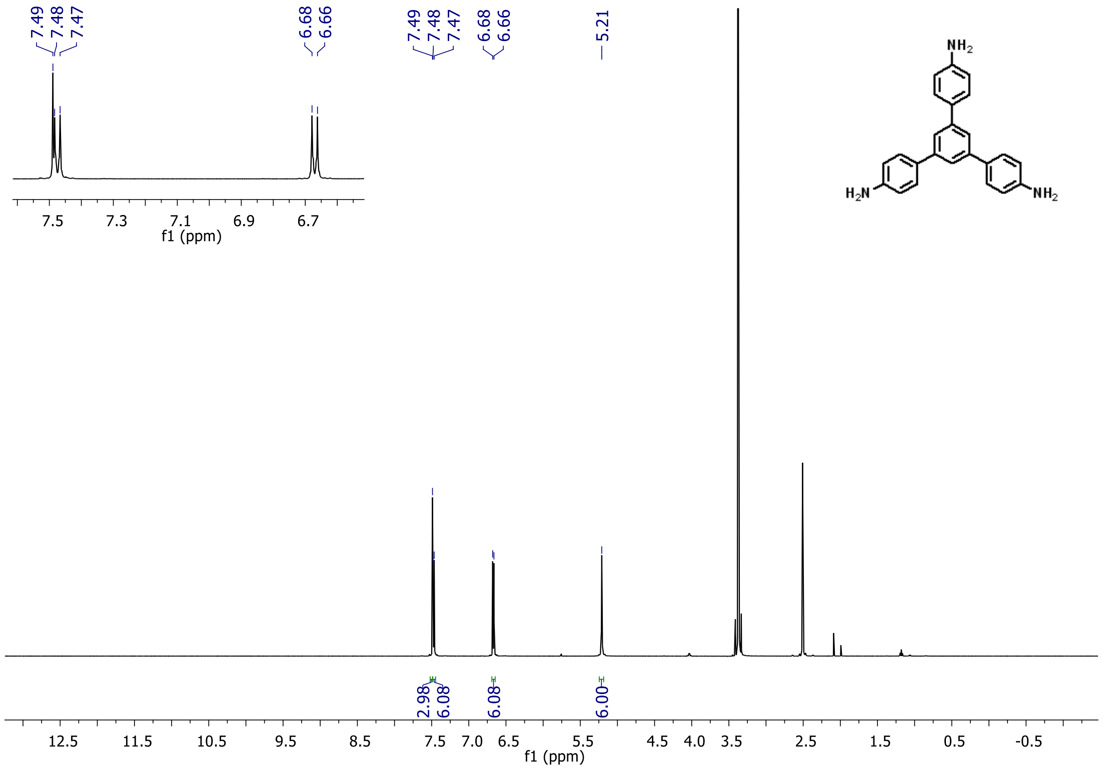


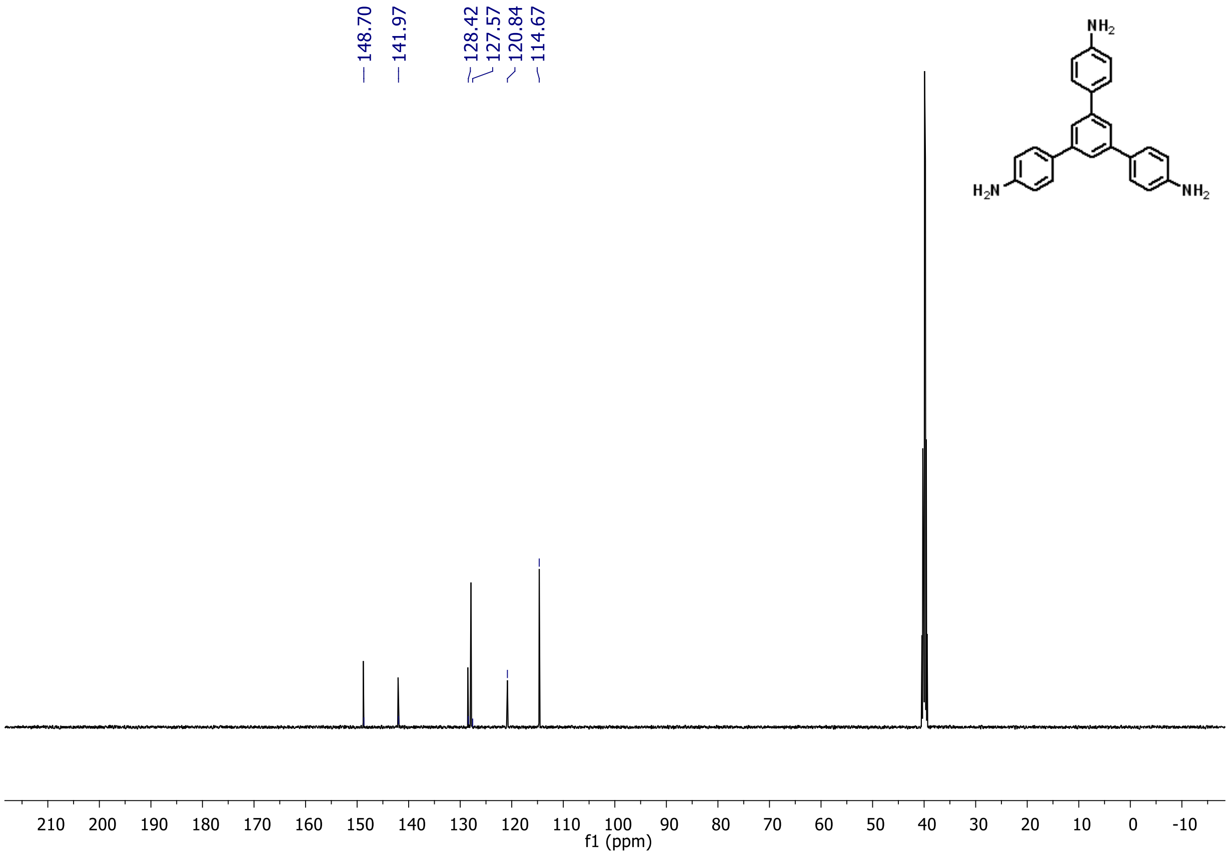


**1,3,5-Tris(4-bromo)benzene[2]**

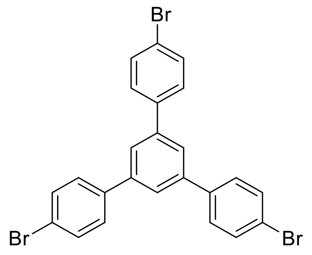


Thionyl chloride (7 mL, 96 mmol, 1.9 equiv.) was added dropwise to a solution of 4-bromoacetophenone (10 g, 50.2 mmol, 1 equiv.) in ethanol (30 mL). The solution was allowed to warm up to ambient temperature and stirred overnight at 60 °C. Afterwards, the suspension was cooled in an ice bath and a saturated solution of $NaHCO_3$ was added carefully. The precipitate was collected and washed three times in hot ethanol and dried under reduced pressure to afforded 1,3,5-tris(4-bromophenyl)benzene as pale yellow solid (5.5 g, 60%).

$^1$ H NMR (500 MHz, $CDCl_3$): δ = 7.45-7,47 (d, 4 H), 7.53-7.54 (d, 6 H), 7.62 (s, 3H). $^{13}$C NMR (100 MHz, CDCl3): δ = 156.43, 141.53, 139.59, 132.06, 128.91, 125.00, 122.12.

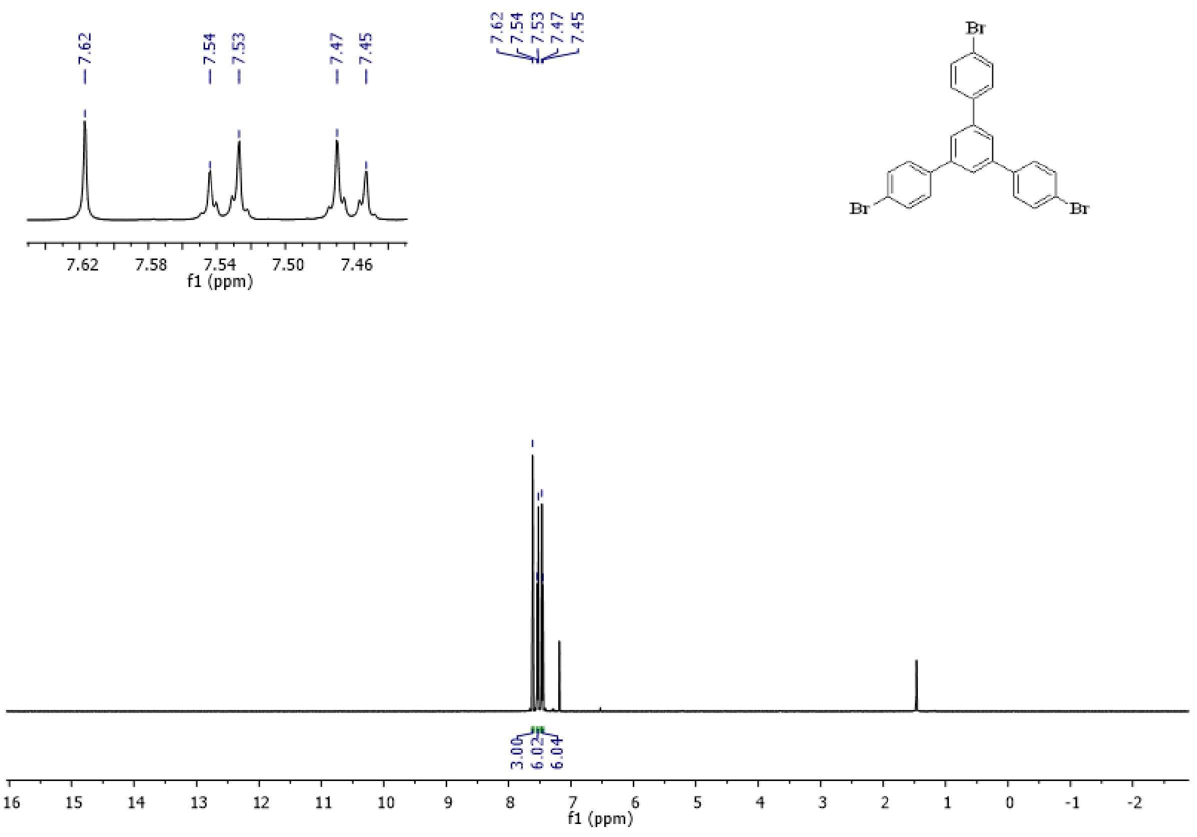


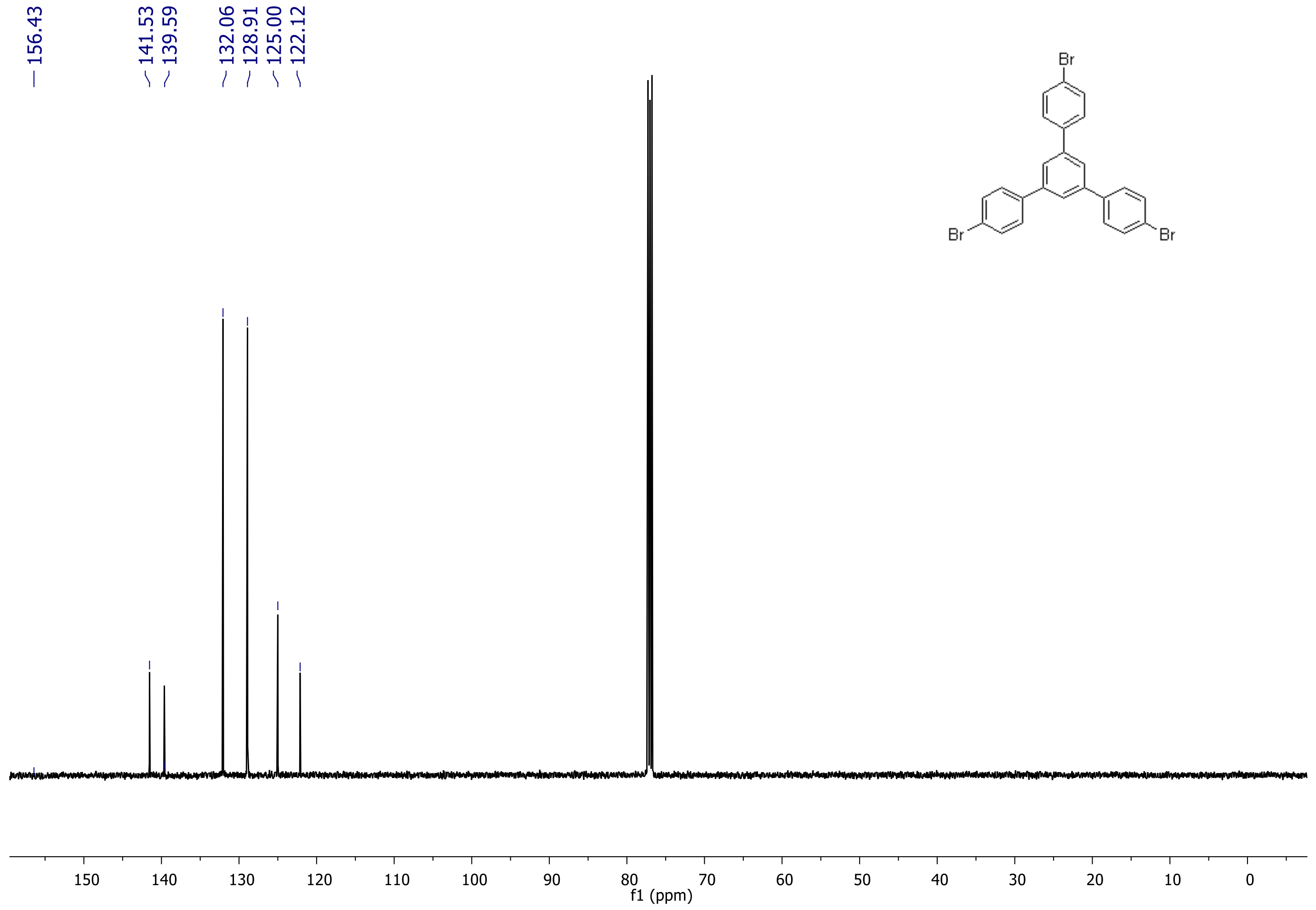


0

**Extended 1,3,5- Tris(aminophenyl)benzene (TAPBext)[1]**

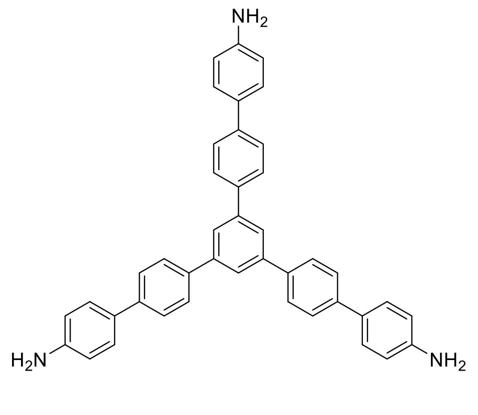


1,3,5-Tris(4-bromo)benzene (2.5 g, 1 equiv., 4.6 mmol) and 4-aminophenylboronic pinacolate (3.22 g, 3.2 equiv., 14.7 mmol) were dissolved in a mixture of THF: toluene (100 mL, 50:50 v/v), followed by addition of NaOH (2.80 g, 15 equiv., 69 mmol). The resulting mixture was degassed for 15 min by a flow of nitrogen, and $Pd(PPh_3)_2Cl_2$ (0.32 g, 0.1 equiv., 0.46 mmol) was added. The solution was degassed again for 10 min by a flow of nitrogen and was heated to 90 ºC for 20 h under nitrogen atmosphere. The reaction mixture was cooled to room temperature and the

solvents were removed under reduced pressure. The remaining crude product was solubilized in hot ethyl acetate and the mixture was hot filtrated over celite, which was washed with hot ethyl acetate many times, and the solvent was removed under reduced pressure. Finally, the obtained dark yellow solid was washed using hot methanol and filtrated. This process was repeated two times to yield a pale-yellow powder (2.0 g, 75% yield). $^{1}$H NMR (500 MHz, DMSO-d6): δ = 7.92 (s, 3H), 7.90 – 7.89 (d, 6H), 7.70 – 7.68 (d, 6H), 7.47-7.45 (d, 6H),  6.69-6.68 (d, 6H), 5.28 (s, 6H). $^{13}$C NMR (100 MHz, DMSO-d6): δ = 148.97, 141.87, 140.40, 137.82, 127.93, 127.61, 127.22, 126.22, 123.93, 114.73.

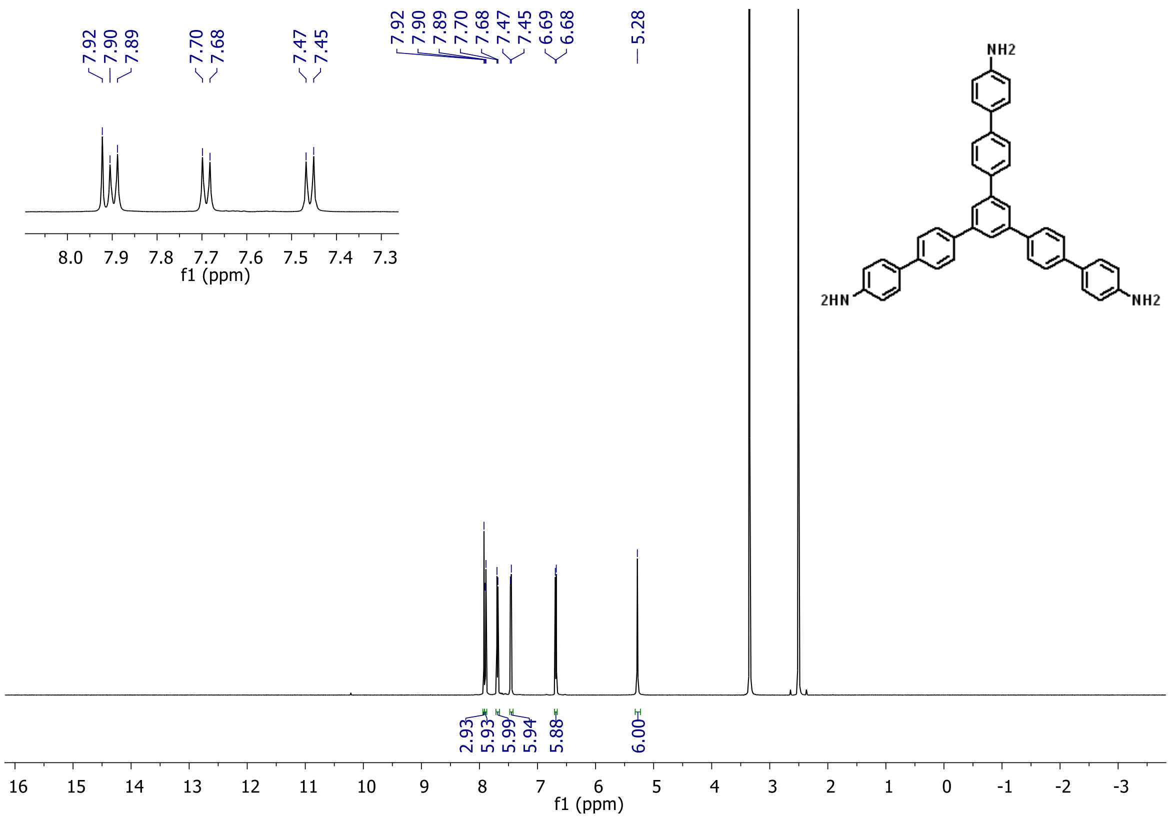


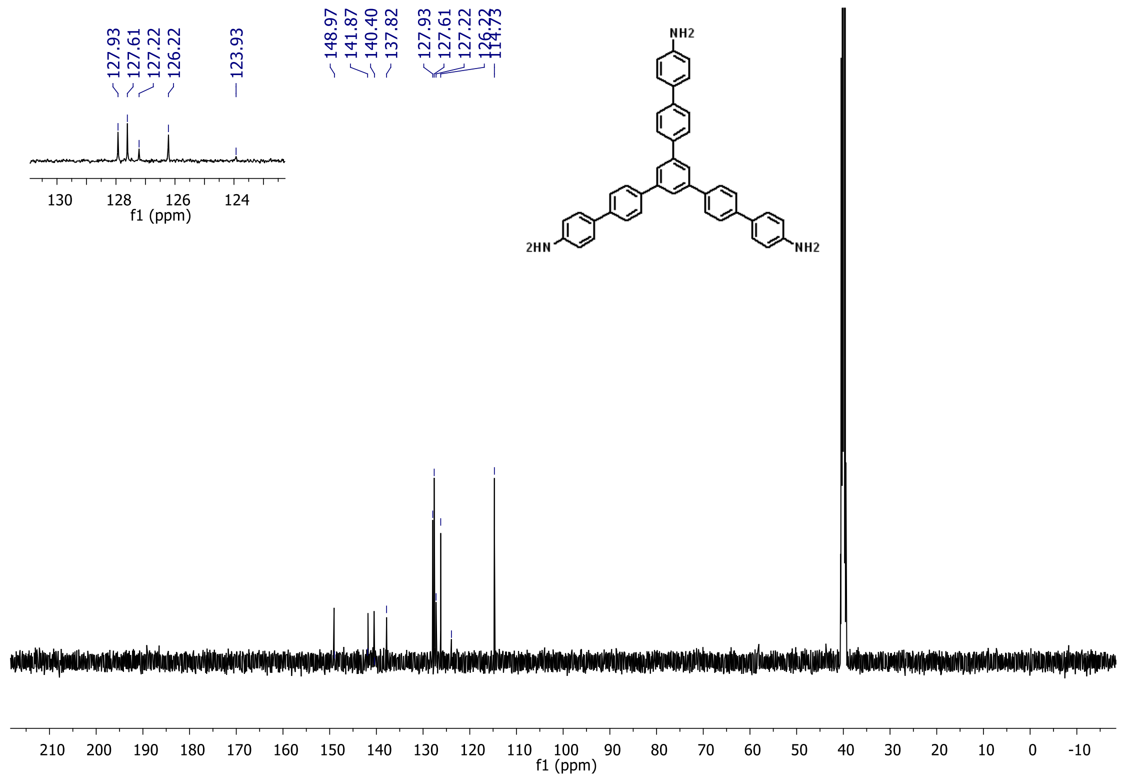


**Extended Aniline 1 (A1)[3]**

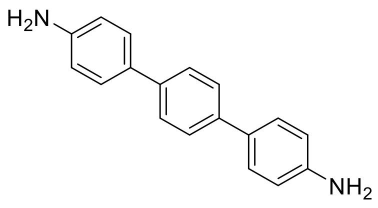


1,4-dibromobenzene (5.0 g, 1 equiv., 21.2 mmol) and 4-aminophenylboronic pinacolate (10.2 g, 2.2 equiv., 46.6 mmol) were disperse in a mixture of toluene:water (100 mL, 50/50, v/v), followed by addition of potassium carbonate (44 g, 15 equiv., 318 mmol). The resulting mixture was degassed for 15 min by a flow of nitrogen, and $Pd(PPh_3)_2Cl_2$ (0.73 g, 0.05 equiv., 1 mmol) was added. The solution was degassed again for 10 min by a flow of nitrogen and was heated to 120 ºC for 20 h under nitrogen atmosphere. The reaction mixture was cooled to room temperature, extracted with EtOAc (3 × 50 mL) and the combined organic extracts were dried over anhydrous MgSO4(s), filtered through celite and the solvent was removed under reduced pressure. The remaining crude product was purified by flash column chromatography on silica gel (40 to 60% EtOAc in

hexanes) to yield a light brown solid **A1** (2.76 g, 50 %).$^{1}$H NMR (500 MHz, DMSO-d6): δ = 7.59 (s, 4H), 7.44 – 7.43 (d, 4H), 6.71 – 6.69 (d, 4H), 5.26 (s, 4H). $^{13}$C NMR (100 MHz, DMSO-d6): δ = 148.64, 138.40, 127.61, 127.30, 126.04, 114.73.

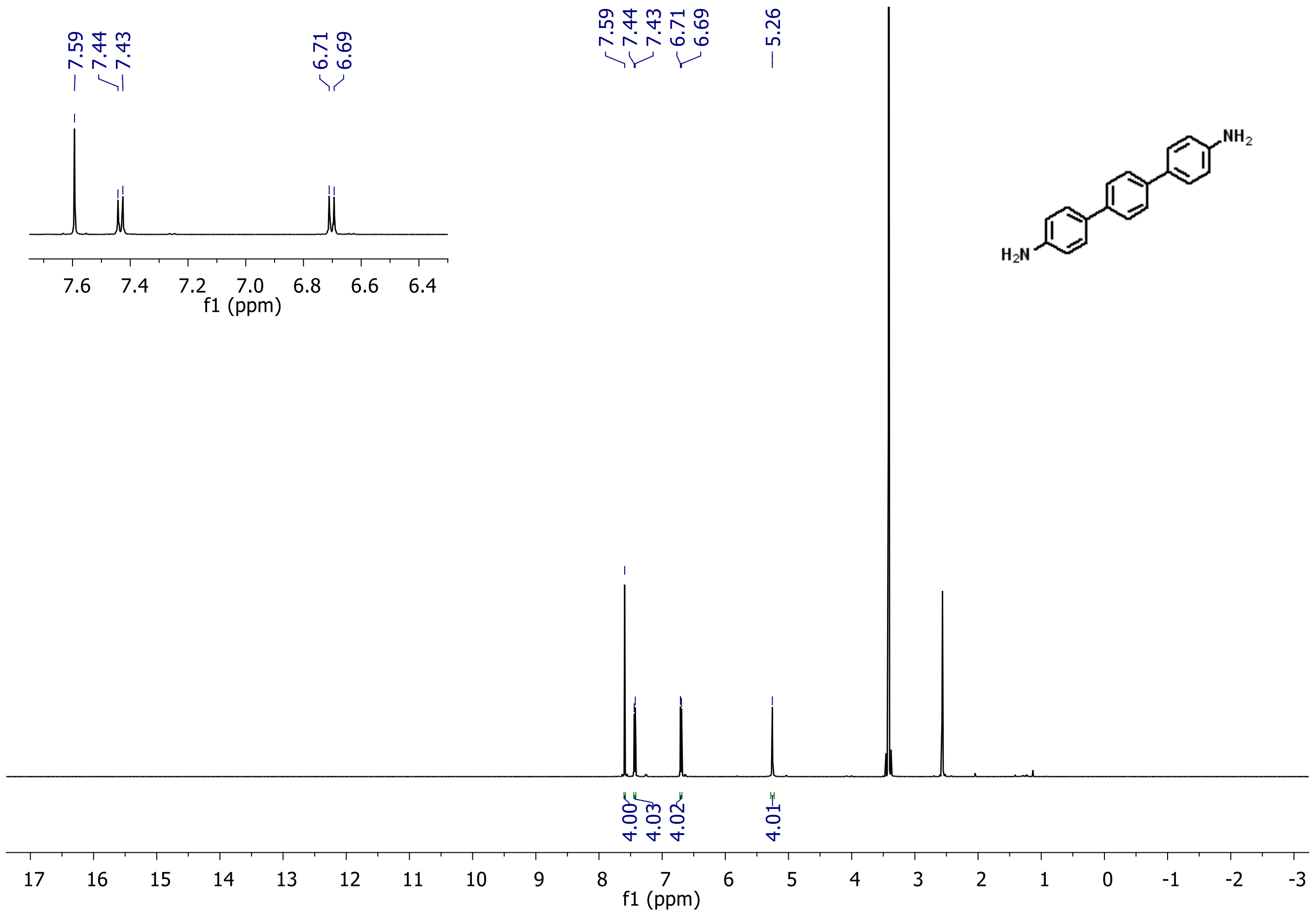


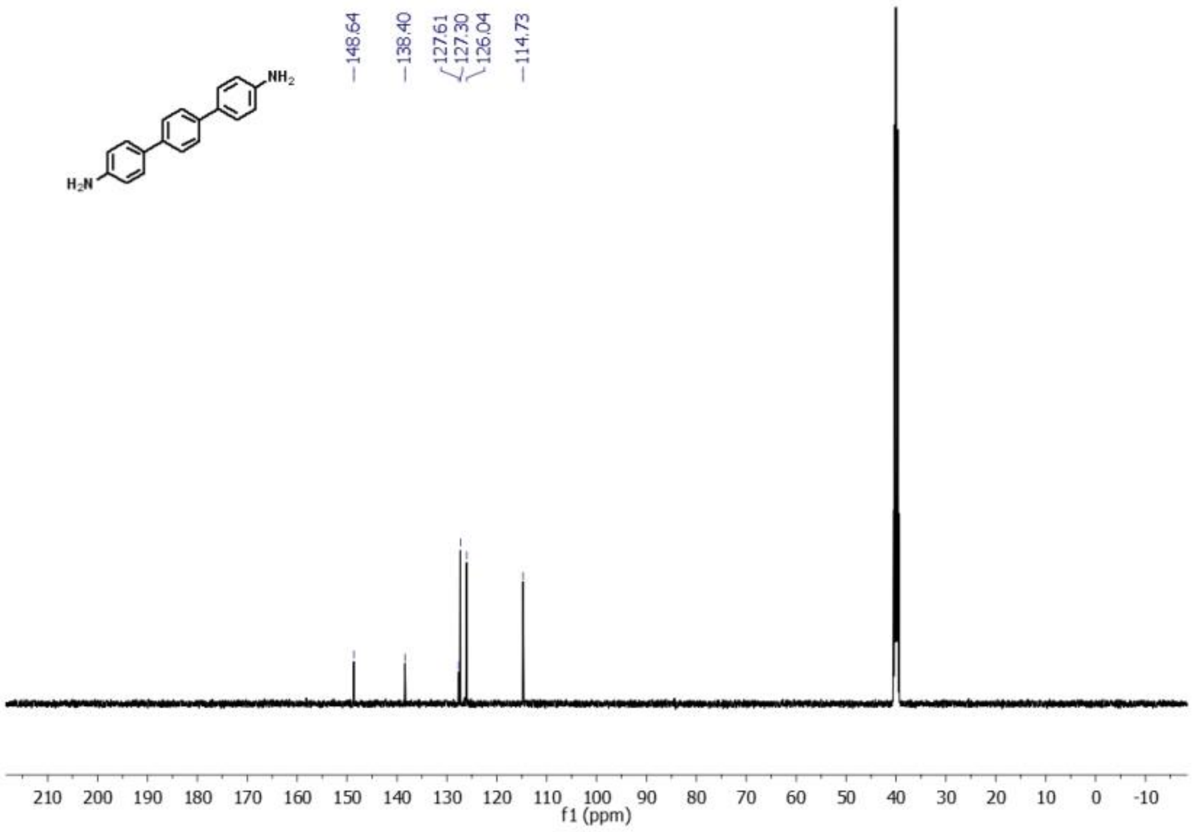


**1,4- dibromo-2,3,5,6-tetramethylbenzene**[4]

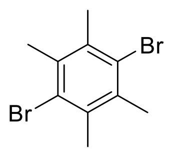


1,2,4,5-tetramethylbenzene (2.5 g, 18.6 mmol, 1 equiv.) was dissolved in DCM (25 mL), followed by addition of $I_2$ (0.1 g, 0.4 mmol). Then, a solution of $Br_2$ (2.4 mL, 47 mmol, 2.6 equiv.) in DCM (20 mL) was added dropwise. The resulting mixture was heated under reflux for 1.5 hours, and after cooling down, 10% NaOH aqueous solution (30 mL) was added. The crude product was collected by filtration and washed with cold DCM, giving the desired product as a white solid (3.5 g, 65%). $^{1}$H NMR (500 MHz, $CDCl_3$): δ = 2.51 (s, 12H). $^{13}$C NMR (100 MHz, $CDCl_3$): 135.04, 128.00, 22.29.

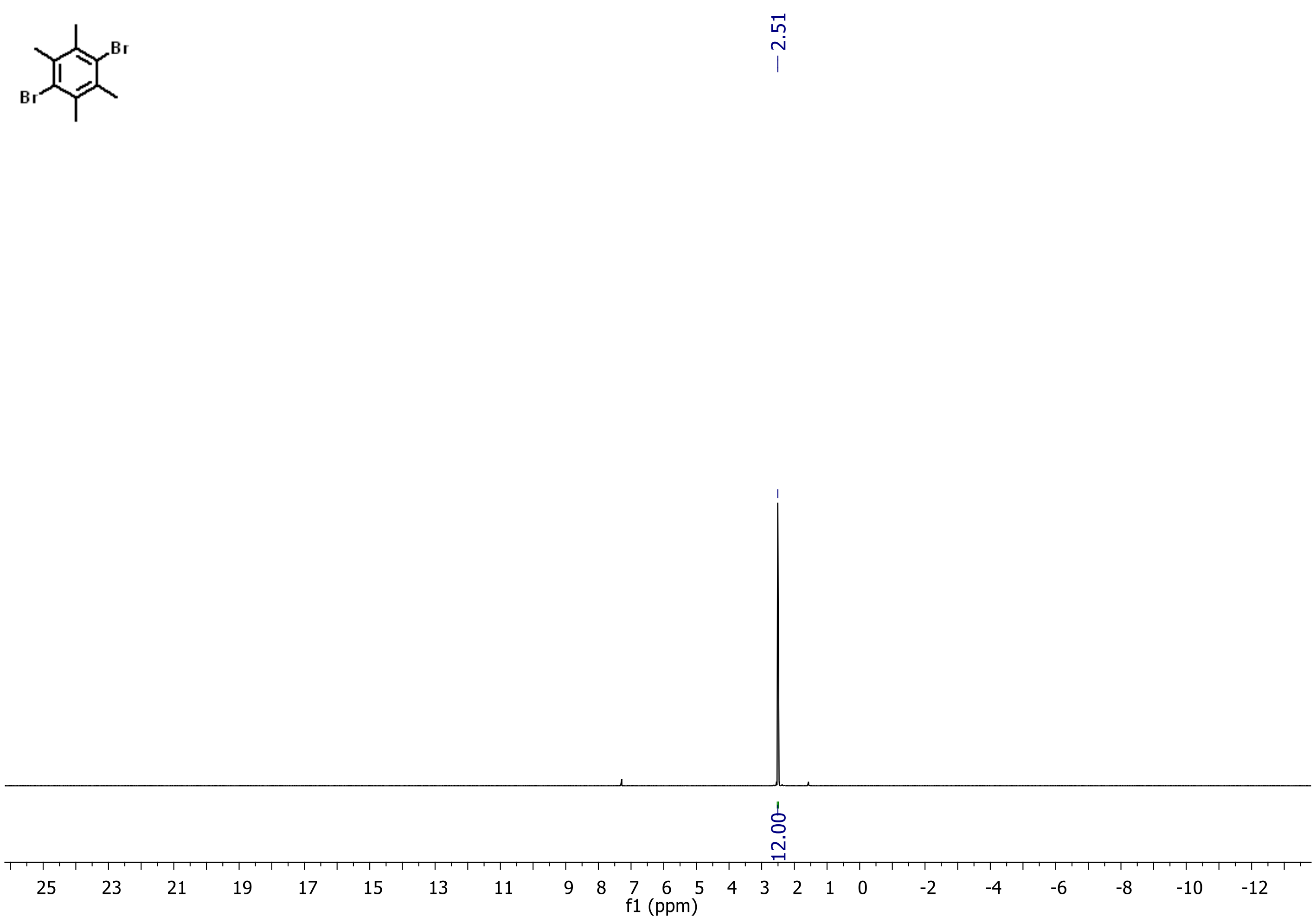


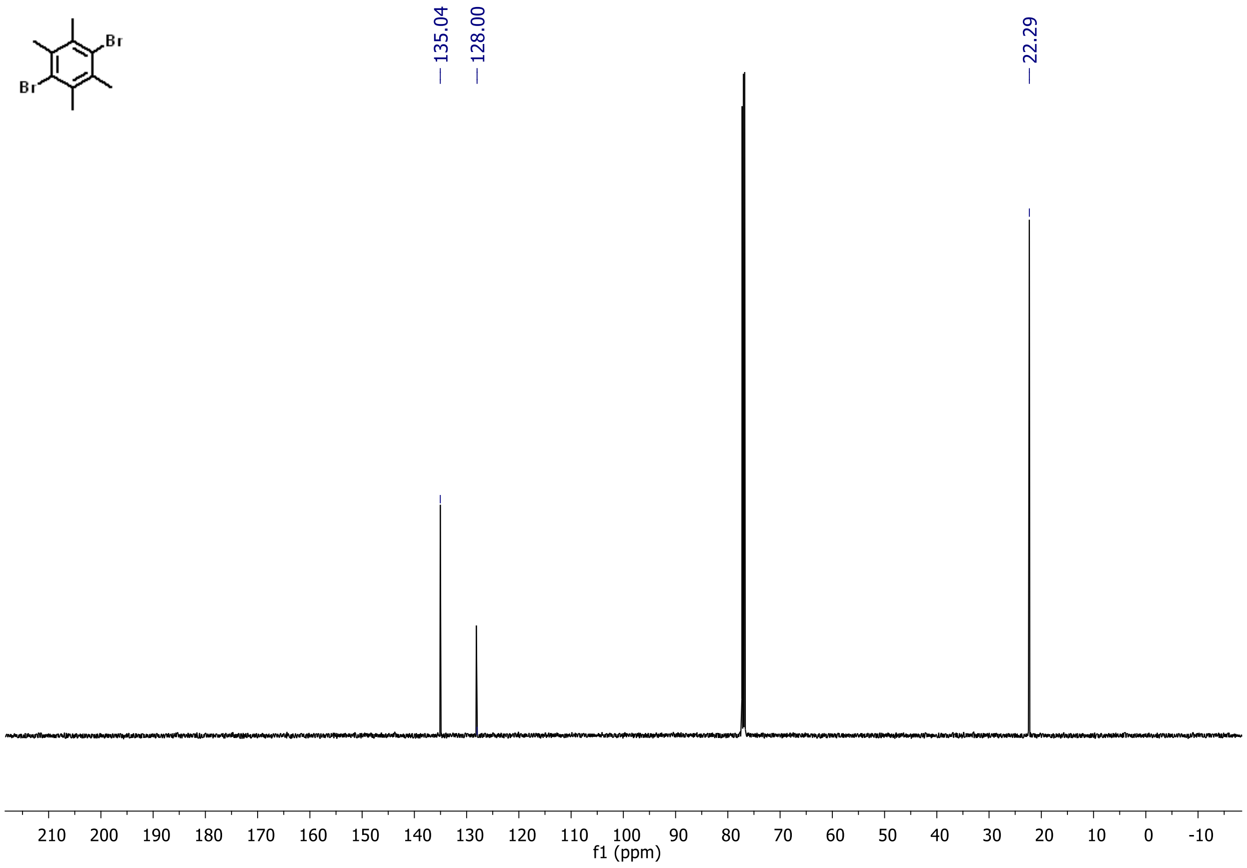


**Extended Aniline 2 (A2)**[4]

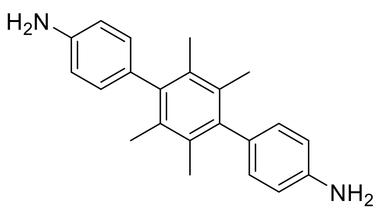


1,4- dibromo-2,3,5,6-tetramethylbenzene (2.2 g, 1 equiv., 7.5 mmol) and 4-aminophenylboronic pinacolate (3.63 g, 2.2 equiv., 16.6 mmol) were dissolved in THF: toluene (100 mL, 50/50 v/v), followed by addition of NaOH (4.52 g, 15 equiv., 113 mmol). The resulting mixture was degassed for 15 min by a flow of nitrogen, and $Pd(PPh_3)_2Cl_2$ (0.30 g, 0.06 equiv., 0.43 mmol) was added. The solution was degassed again for 10 min by a flow of nitrogen and was heated to 90 ºC for 20 h under nitrogen atmosphere. The reaction mixture was cooled to room temperature and the solvent was removed under reduced pressure. The crude product was purified by flash column chromatography on silica gel (20 to 100%

EtOAc in hexanes) and the dark brown solid obtained was recrystallized from DCM/methanol (1:4) to give a light brown solid (0.9 g, 42 % yield). $^{1}H$ NMR (500MHz, DMSO-d6): δ = 6.75 – 6.73 (d, 4H), 6.64 – 6.62 (d, 4H), 5.02 (s, 4H), 1.88 (s, 12H). $^{13}C$ NMR (100 MHz, DMSO-d6): δ = 145.80, 140.22, 132.11, 131.87, 131.04, 129.38, 127.57, 121.16, 114.01, 20.42, 17.98.

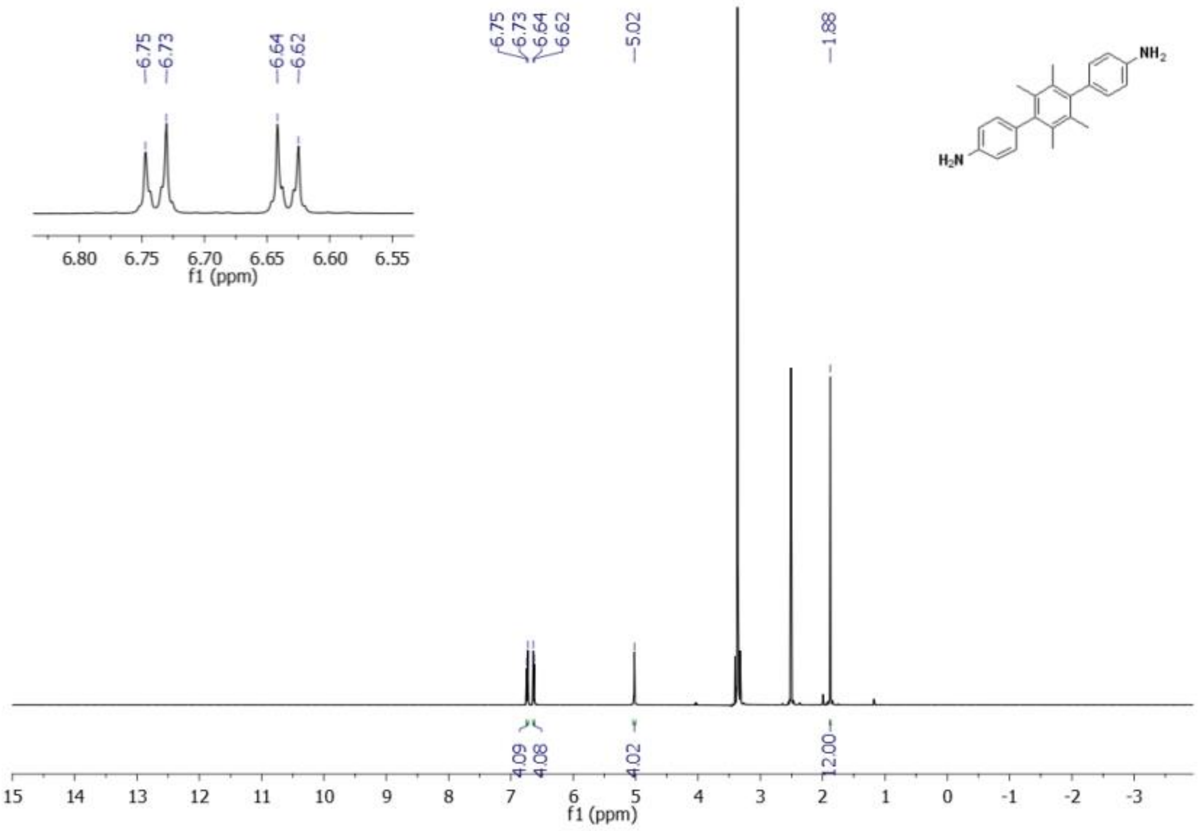


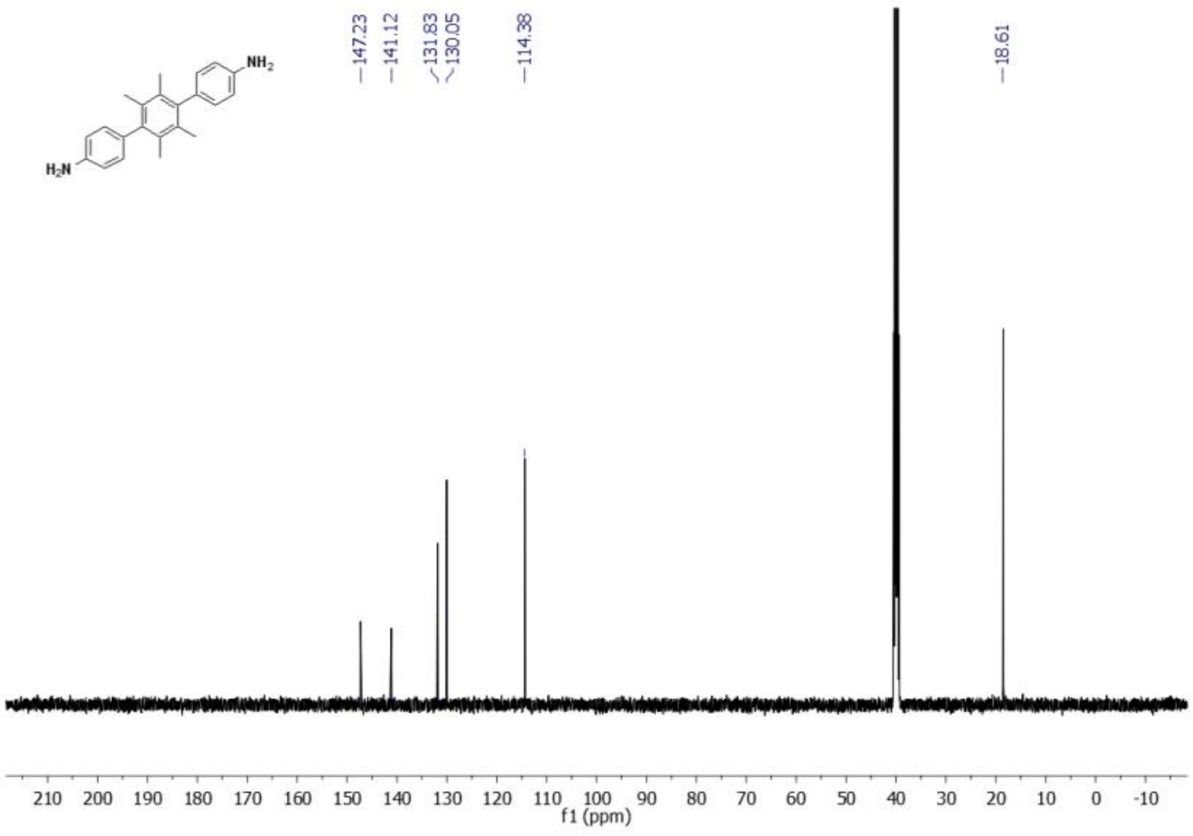


**1,4-Bis(pinacolatoboronyl)-2,5-dimethylbenzene[5]**

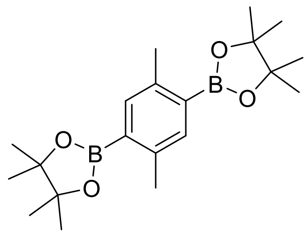

1,4-dibromo-2,5-dimethylbenzene (2.0 g, 1 equiv., 7.6 mmol) and bis(pinacolato)diboron (5.8 g, 3 equiv., 22.8 mmol), potassium acetate (4.5 g, 6 equiv., 45.6 mmol) and dimethylformamide, DMF (80 mL). The resulting mixture was degassed for 15 min by a flow of nitrogen, and $Pd(PPh_3)_4$ (0.56 g, 0.06 equiv., 0.5 mmol) was added. The resulting mixture was degassed again for 10 min by a flow of nitrogen and was heated to 85 ºC for 24 under nitrogen atmosphere. The reaction mixture was cooled down to room temperature, then added to water before being extracted with dichloromethane. Combined organic layers were washed with water and brine, then dried with magnesium sulfate and evaporated under

reduced pressure. The crude product was purified by column chromatography (50 to 70 % dichloromethane in n-hexane) to give a white solid (2.15 g, 80 %).

$^1$ H NMR (500 MHz, $CDCl_3$): δ = 7.58 (s, 2 H), 2.53 (s, 6 H), 1.38 (s, 24 H). $^3$C NMR(100 MHz, $CDCl_3$): δ= 140.56, 136.94, 88.42, 24.90, 21.51.

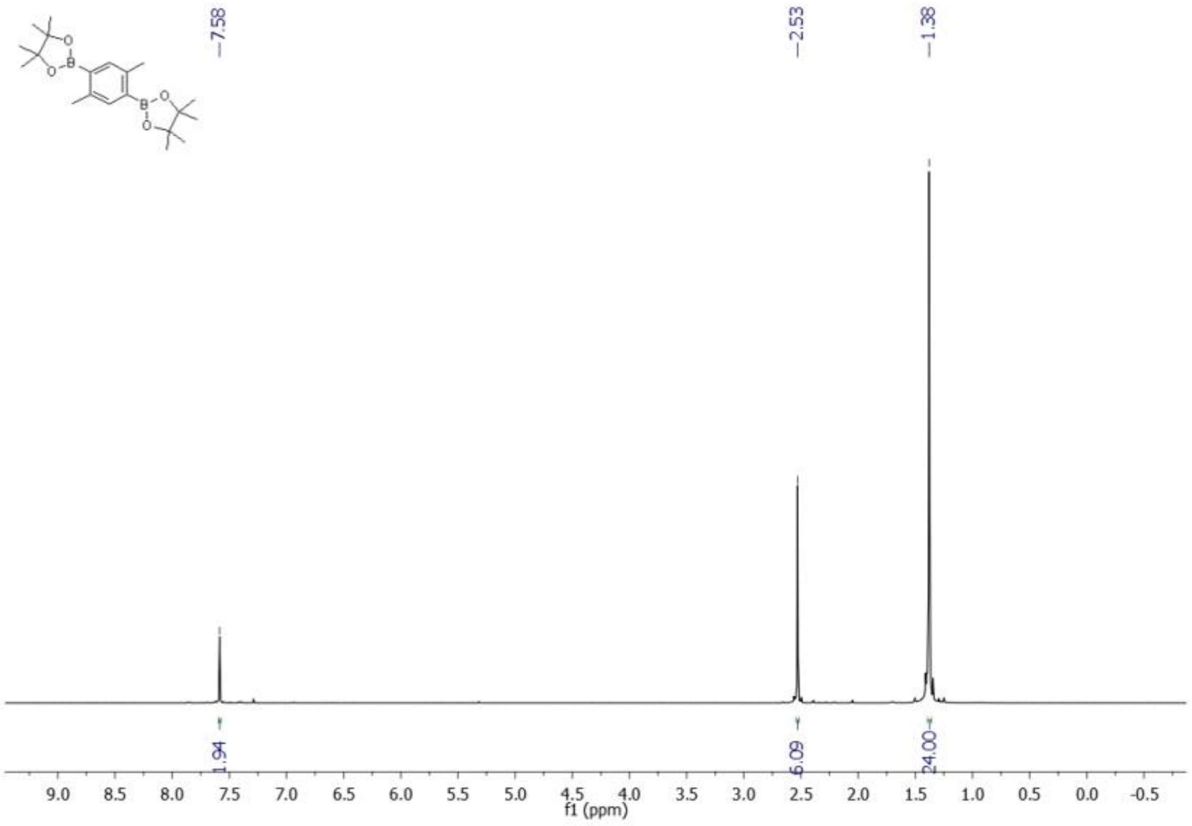


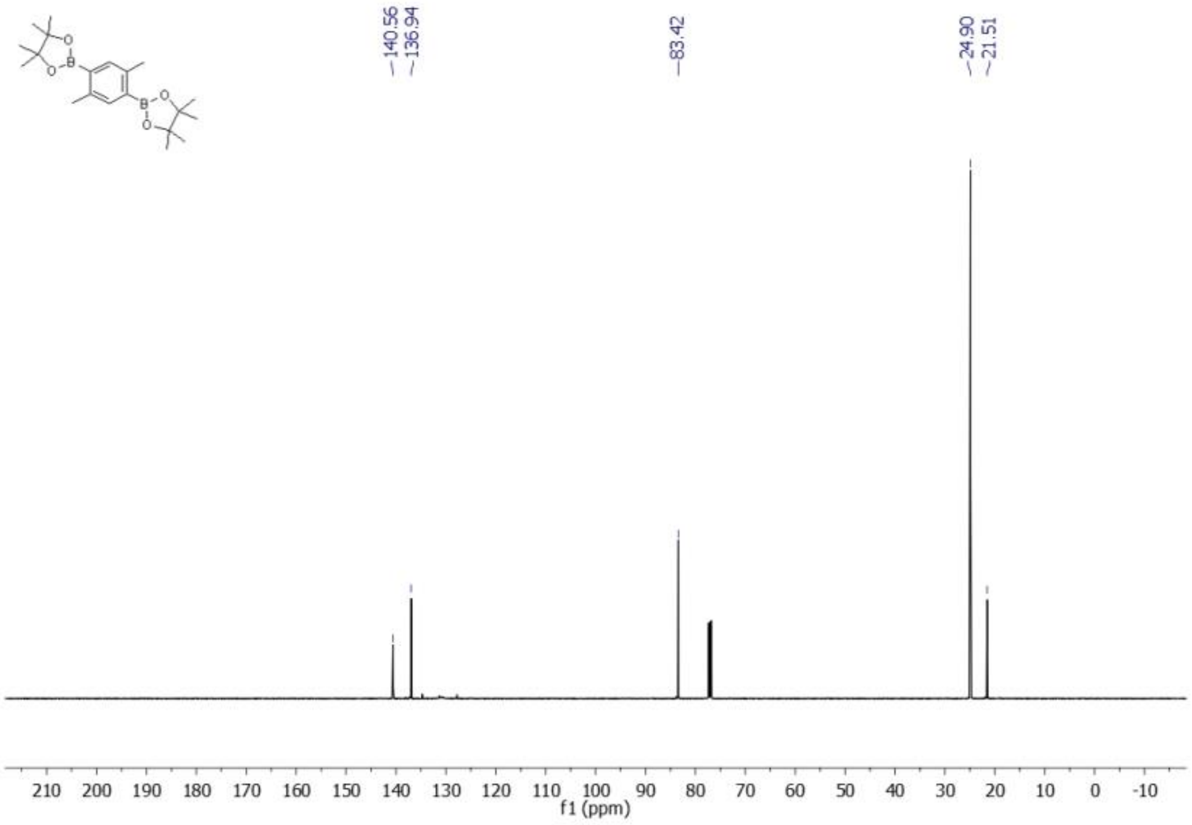


**Extended aniline 3 (A3)**

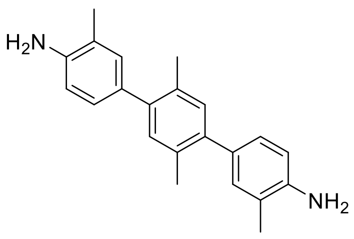


A two-necked round bottom flask (250 mL) was equipped with a reflux condenser, magnetic stir-bar, septum, and it was charged with 1,4-Bis(pinacolatoboronyl)-2,5-dimethylbenzene (2.0 g, 1 equiv., 5.6 mmol), 4-bromo-2methyl aniline (2.8 g, 2.7 equiv., 15 mmol), NaOH (3.36 g, 15 equiv., 84 mmol), toluene (50 mL) and THF (50 mL). The resulting mixture was degassed for 15 min by a flow of nitrogen, and $Pd(PPh_3)_4$ (0.55 g, 0.09 equiv., 0.48 mmol) was added. The resulting mixture was degassed again for 10 min by a flow of nitrogen and was heated to 90 ºC for 18 h under nitrogen atmosphere. The reaction mixture was cooled to room temperature and the solvent was removed under reduced pressure.

The crude product was purified by flash column chromatography on silica gel (20 to 100% EtOAc in hexanes) and the obtained dark brown solid was washed with methanol (20 mL) to give a light brown solid (1.2 g, 71 % yield).

$^{1}H$ NMR (500 MHz, DMSO-d6): δ= 6.98 (s, 2H), 6.92 (s, 2H), 6.90 – 6.89 (d, 2H), 6.66 – 6.65 (d, 2H), 4.86 (s, 4H), 2.20 (s, 6H), 2.10 (s, 6H).

$^{13}C$ NMR (100 MHz, DMSO-d6): δ= 145.80, 140.22, 132.11, 131.87, 131.04, 129.38, 127.57, 121.16, 114.01, 20.42, 17.98.

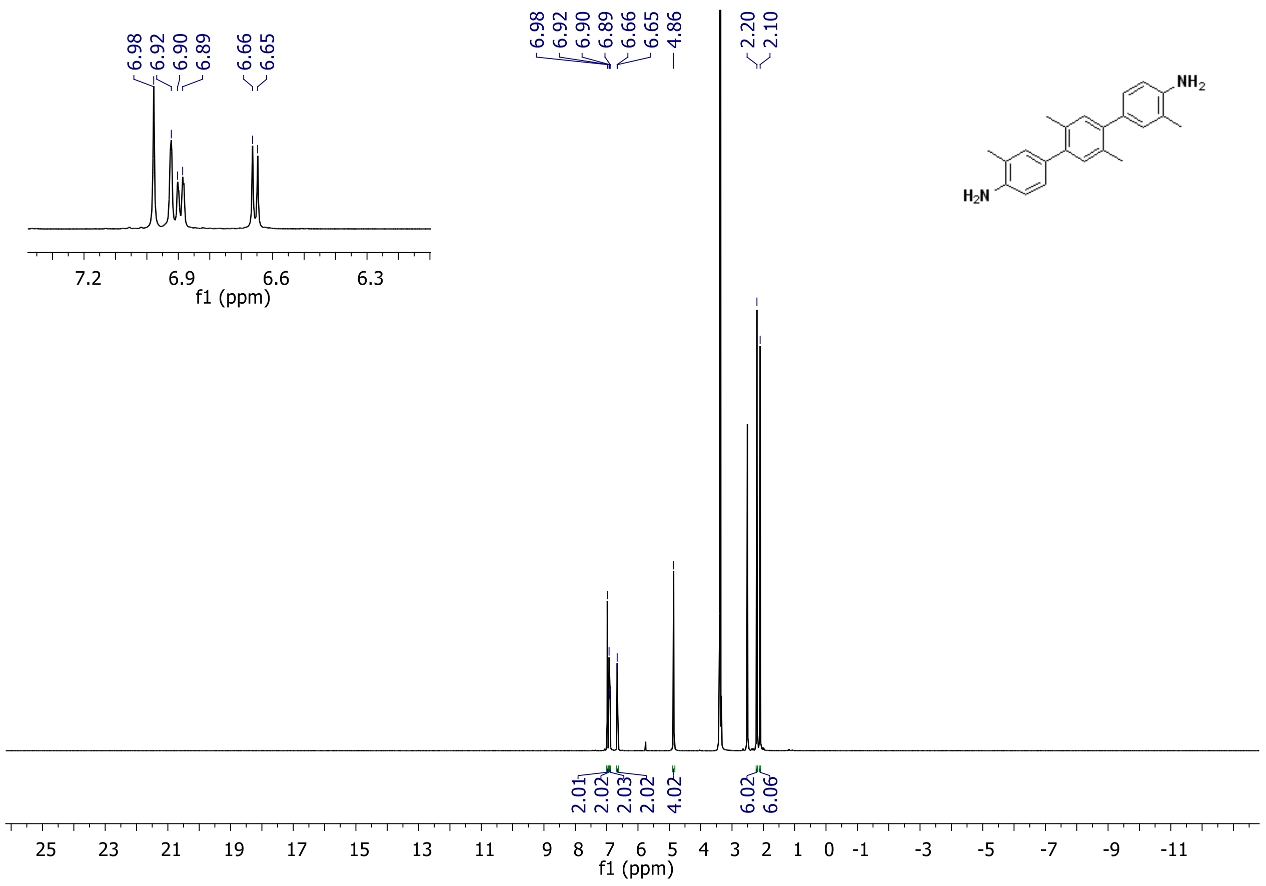


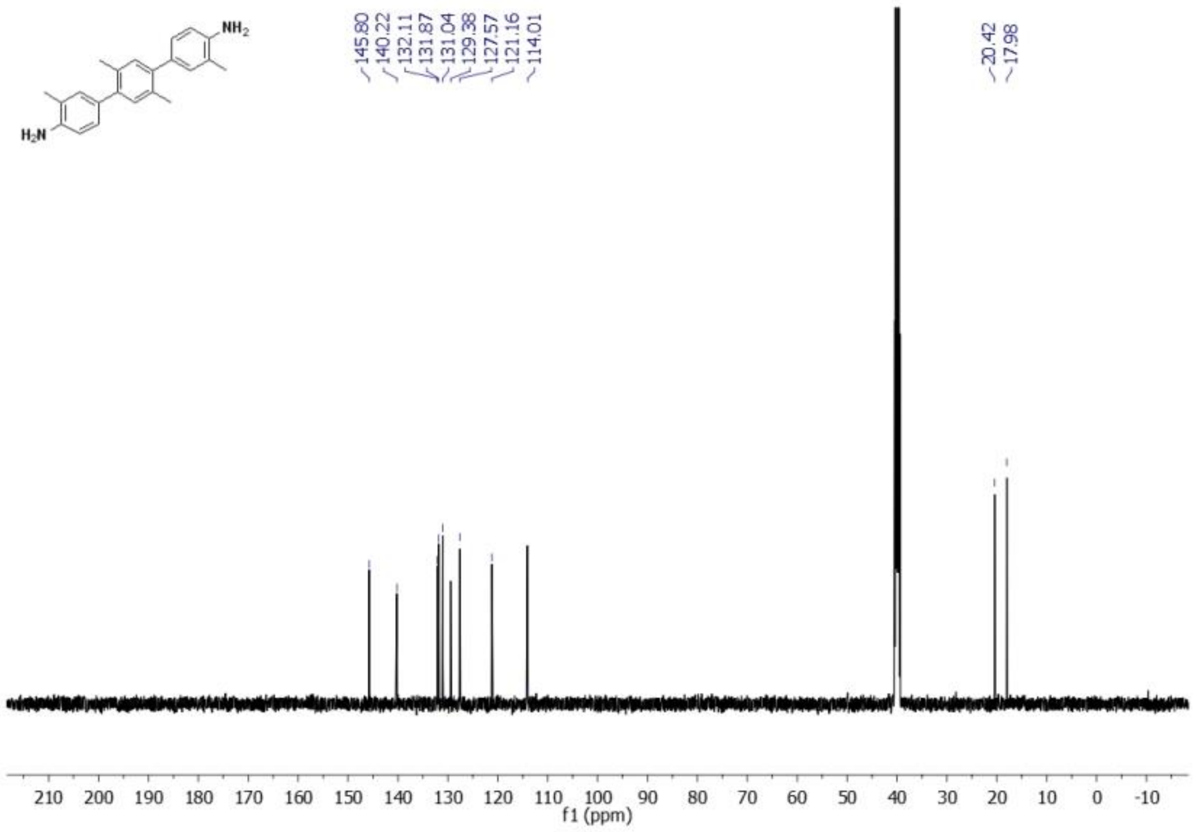


*

## 3. Synthesis of Polymers and co-polymers

### General procedure

The polymers were prepared following literature procedure with some modifications.[6] The appropriated monomer (1 equiv) was reacted with dimethoxymethane (8 equiv) in DCM (approximately 10 mL), followed by dropwise addition of trifluoroacetic acid, TFA (37 equiv). The reaction was left to stir at room temperature for approximately 16 h and was crashed out in ammonia/ice and stirred overnight. The product was then filtered, washed with plenty of water, and refluxed in acetone, THF, DCM and methanol, before being dried in a vacuum oven at 85 $^{0}$C for 20 h. The co-polymers were prepared in the same way, using the combination of two monomers: A (1 equiv) and B (1.5 equiv) (Table S1).

**TAPB-PIM**

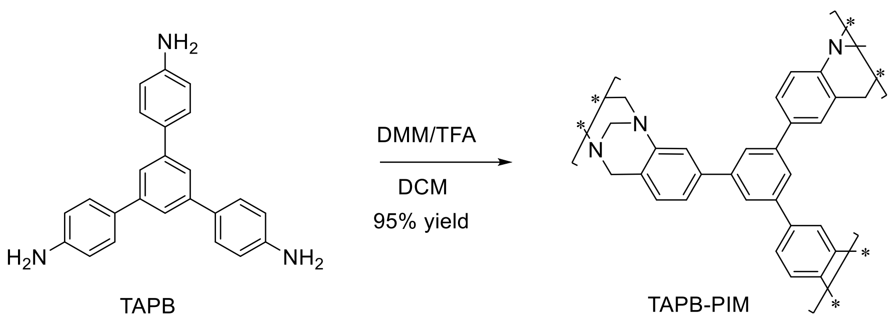


Tris(4-aminophenyl)benzene (TAPB) (0.7 g, 2 mmol) and dimethoxymethane (1.4 mL, 15.8 mmol) were stirred in DCM (14 mL), followed by dropwise addition of TFA (5.7 mL, 74.5 mmol) to yield a pale-yellow solid (0.76 g, 95% yield).
BET ($CO_2$, 273 K) = 500 $m^2$ $g^{-1}$, total pore volume =0.13 (at $P/P_0$ ~ 0.98). TGA: initial mass loss at 440 °C. FT-IR ν max ($cm^{-1}$) 2900, 1610, 1593, 1513, 1205, 950, 825. $^{13}C$ NMR SS (101 MHz) δ 161.6, 147.1, 141.9, 127.3, 66.8, 59.7, 49.4, 29.3, 16.2, 1.9.

**TAPBext-PIM**

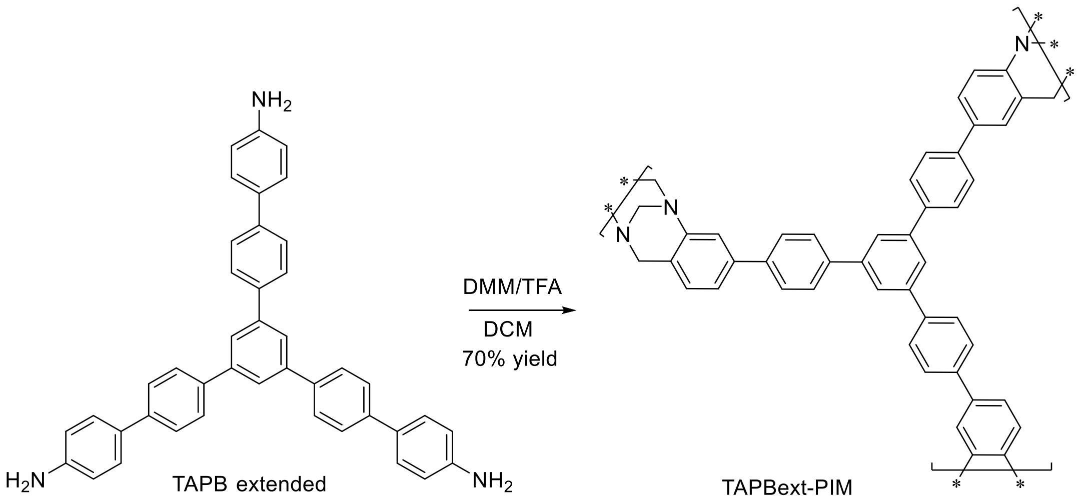


Extended Tris(4-aminophenyl)benzene (TAPBext) (0.5 g, 0.86 mmol) and dimethoxymethane (0.6 mL, 6.8 mmol) were stirred in DCM (10 mL), followed by dropwise addition of TFA (2.5 mL, 32.7 mmol) to yield a pale-yellow solid (0.38 g, 70% yield).
BET ($CO_2$, 273 K) = 350 $m^2$ $g^{-1}$, total pore volume =0.07 (at $P/P_0$ ~ 0.98). TGA: initial mass loss at 495 °C. FT-IR ν max ($cm^{-1}$) 1590, 1490, 813. $^{13}C$ NMR SS (101 MHz) δ 158.0, 147.4, 140.4, 126.9, 67.3, 59.8, 49.5, 39.0, 16.1, 2.4.

**TAPB(Tol)-PIM**

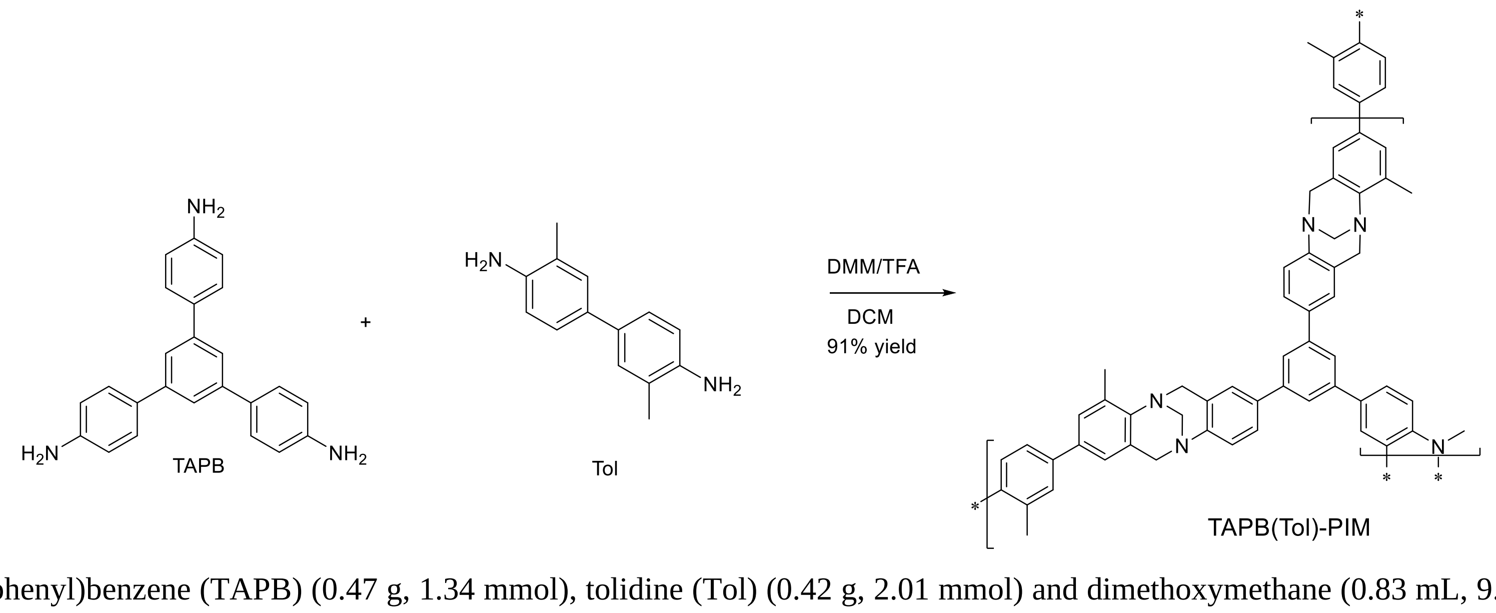


Tris(4-aminophenyl)benzene (TAPB) (0.47 g, 1.34 mmol), tolidine (Tol) (0.42 g, 2.01 mmol) and dimethoxymethane (0.83 mL, 9.4 mmol) were stirred in DCM (8 mL), followed by dropwise addition of TFA (3.5 mL, 45.8 mmol) to yield an orange-brown solid (0.75 g, 91%)
BET ($CO_2$, 273 K) = 360 $m^2$ $g^{-1}$, total pore volume = 0.078 (at $P/P_0$ ~ 0.98). TGA: initial mass loss at 440 °C. FT-IR ν max ($cm^{-1}$) 2893, 1667, 1199, 825. $^{13}C$ NMR SS (101 MHz) δ 162.0, 147.6, 144.4, 138.4, 127.4, 80.9, 67.4, 55.7, 27.3, 16.1, 3.2.

**TAPB(A1)-PIM**

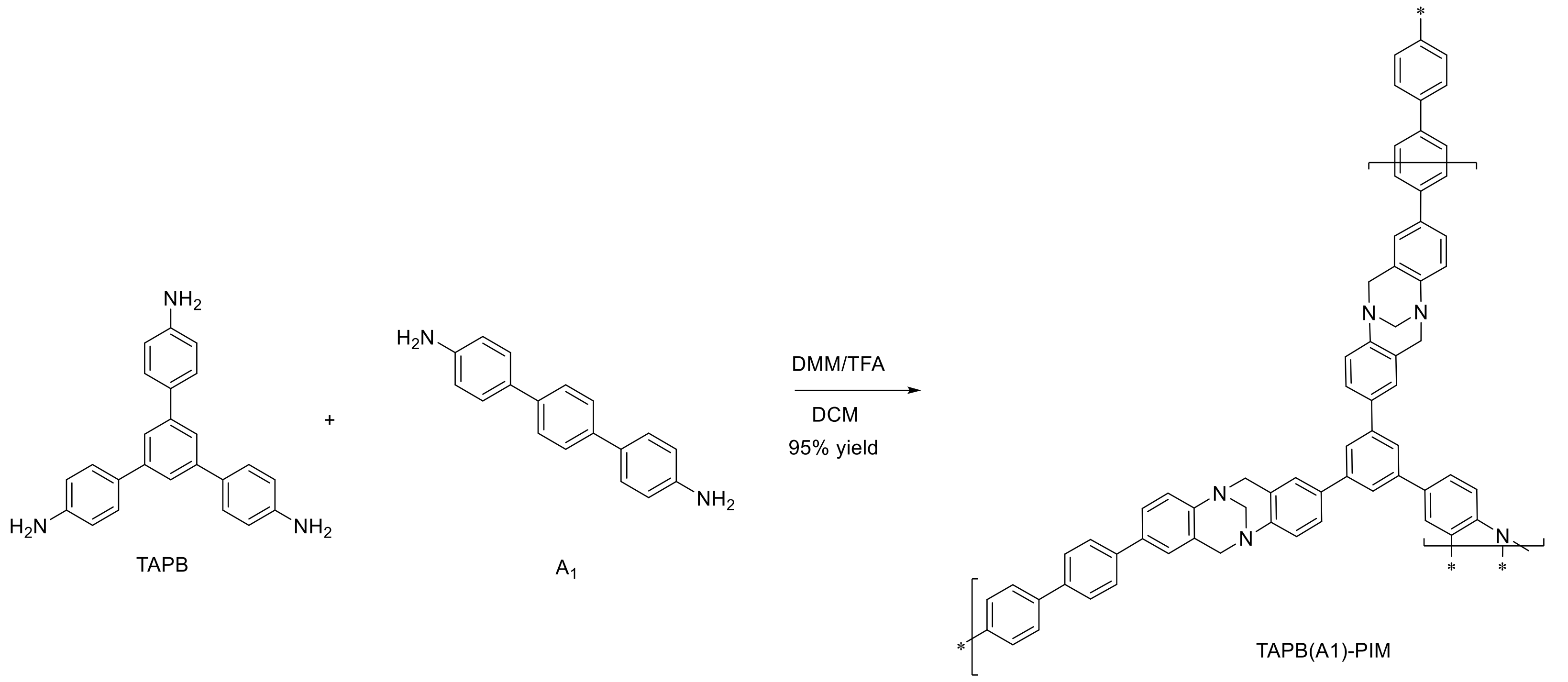


Tris(4-aminophenyl)benzene (TAPB) (0.5 g, 1.42 mmol), A1 (0.56 g, 2.13 mmol) and dimethoxymethane (1.0 mL, 11.3 mmol) were stirred in DCM (10 mL), followed by dropwise addition of TFA (4.2 mL, 55 mmol) to yield a pale-yellow solid (0.9 g, 95% yield).

BET ($CO_2$, 273 K) = 330 $m^2$ $g^{-1}$, total pore volume =0.08 (at $P/P_0$ ~ 0.98). TGA: initial mass loss at 440 °C. FT-IR ν max ($cm^{-1}$) 1670, 1606, 1480, 1200, 944, 818. $^{13}C$ NMR SS (101 MHz) δ 157.5, 147.3, 139, 126.7, 67.0, 59.6, 50.7, 42.3, 15.8, 2.7.

**TAPB(A2)-PIM**

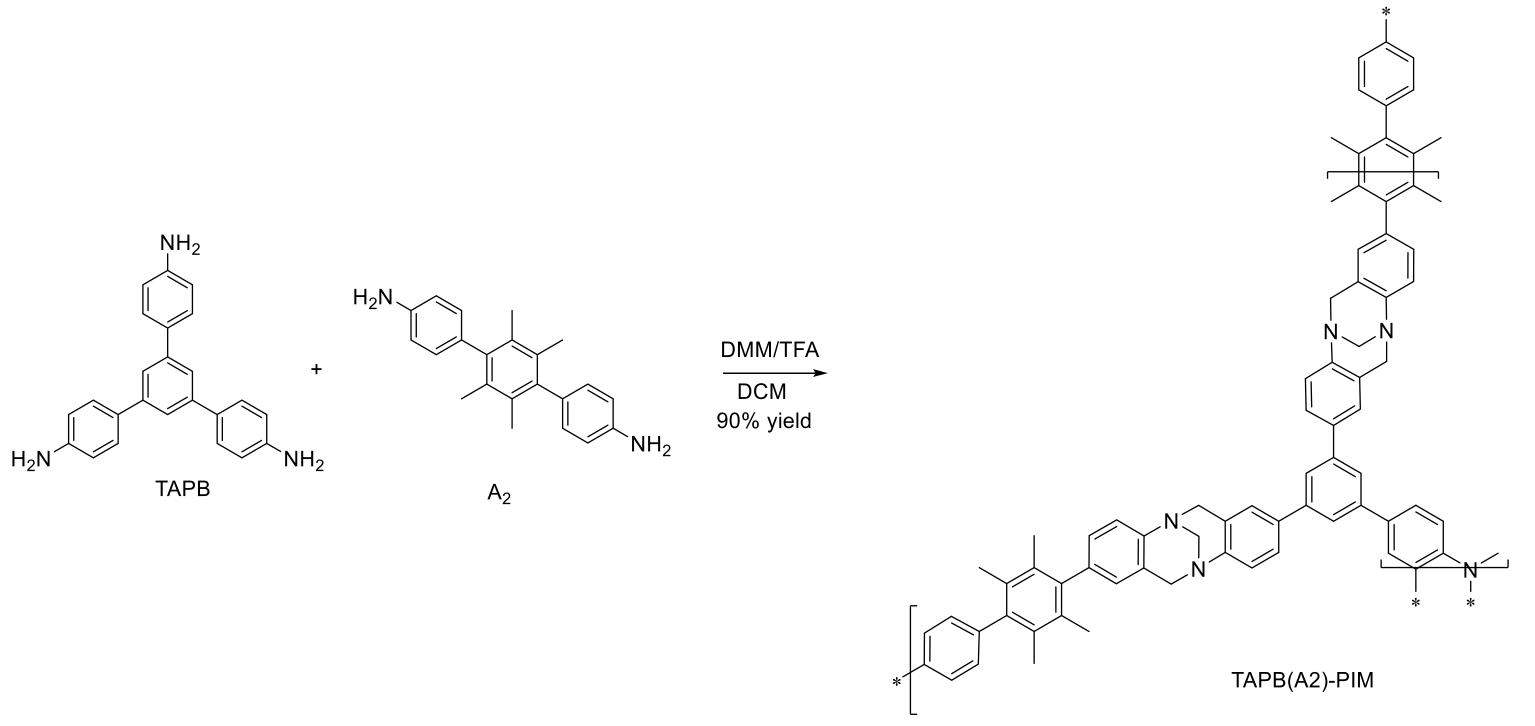


Tris(4-aminophenyl)benzene (TAPB) (0.46 g, 1.30 mmol), A2 (0.62 g, 1.95 mmol) and dimethoxymethane (1.0 mL, 11.3 mmol) were stirred in DCM (10 mL), followed by dropwise addition of TFA (4.0 mL, 52 mmol) to yield a pale-yellow solid (0.86 g, 90% yield).

BET ($CO_2$, 273 K) = 370 $m^2$ $g^{-1}$, total pore volume =0.08 (at $P/P_0$ ~ 0.98). TGA: initial mass loss at 440 °C. FT-IR ν max ($cm^{-1}$) 2900, 1660, 1600, 1515, 1207, 950, 830. $^{13}C$ NMR SS (101 MHz) δ 157.0, 146.9, 140.4, 130.4, 128.0, 67.3, 59.6, 50.2, 32.0, 17.1, 3.7.

**TAPB(A3)-PIM**

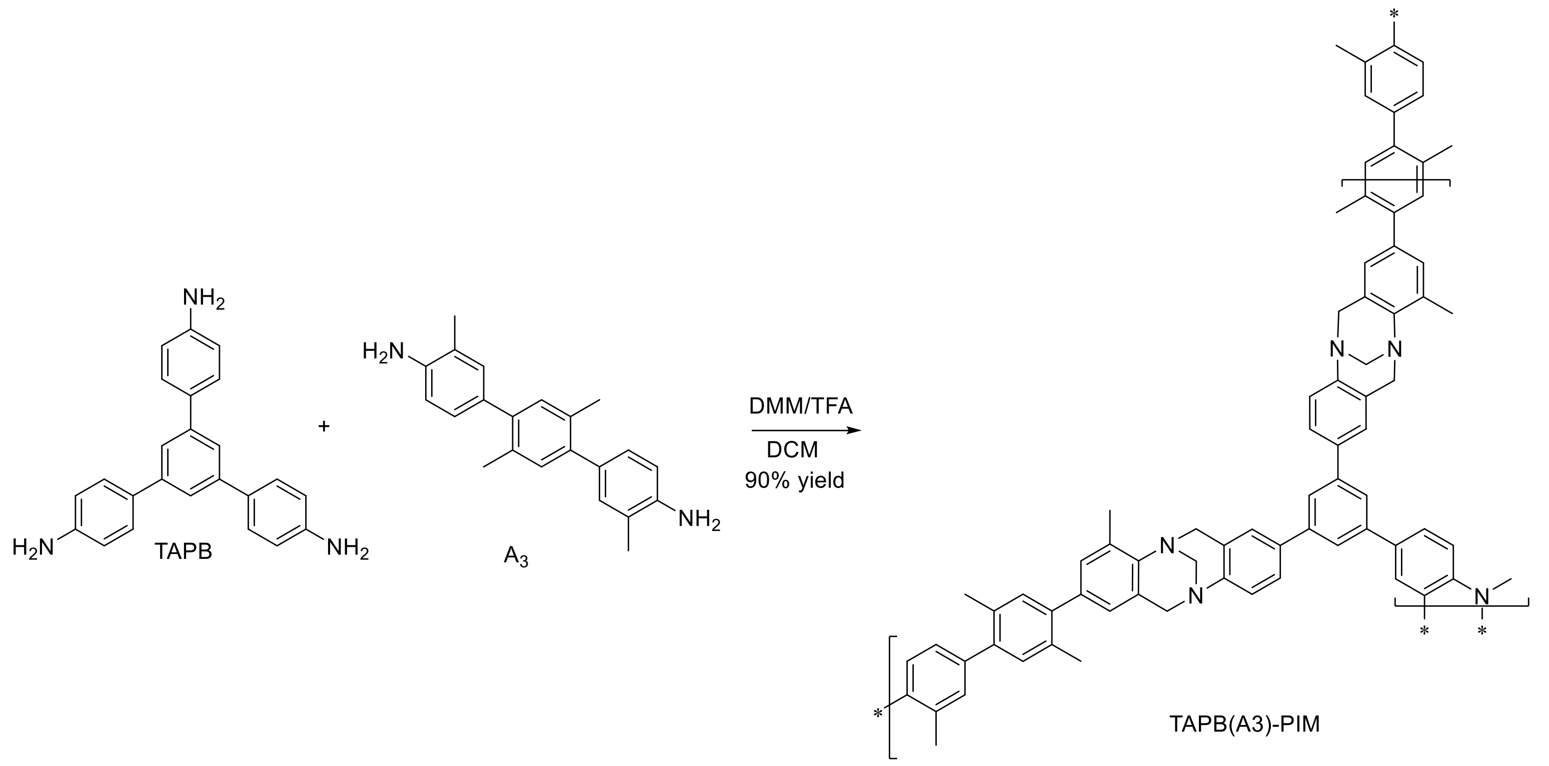


Tris(4-aminophenyl)benzene (TAPB) (0.5 g, 1.42 mmol), A2 (0.67 g, 2.13 mmol) and dimethoxymethane (1.0 mL, 11.3 mmol) were stirred in DCM (10 mL), followed by dropwise addition of TFA (4.2 mL, 52 mmol) to yield a pale-yellow solid (0.86 g, 90% yield).
BET ($CO_2$, 273 K) = 350 $m^2 g^{-1}$, total pore volume =0.07 (at $P/P_0$ ~ 0.98). TGA: initial mass loss at 440 °C. FT-IR ν max ($cm^{-1}$) 2960, 1670, 1590, 1470, 1210, 1060, 940, 865, 830. $^{13}$C NMR SS (101 MHz) δ 158.0, 147.3, 143.9, 138.1, 128.0, 67.5, 55.7, 46.2, 25.7, 17.5, 3.4.

**TAPB(EA)-PIM**

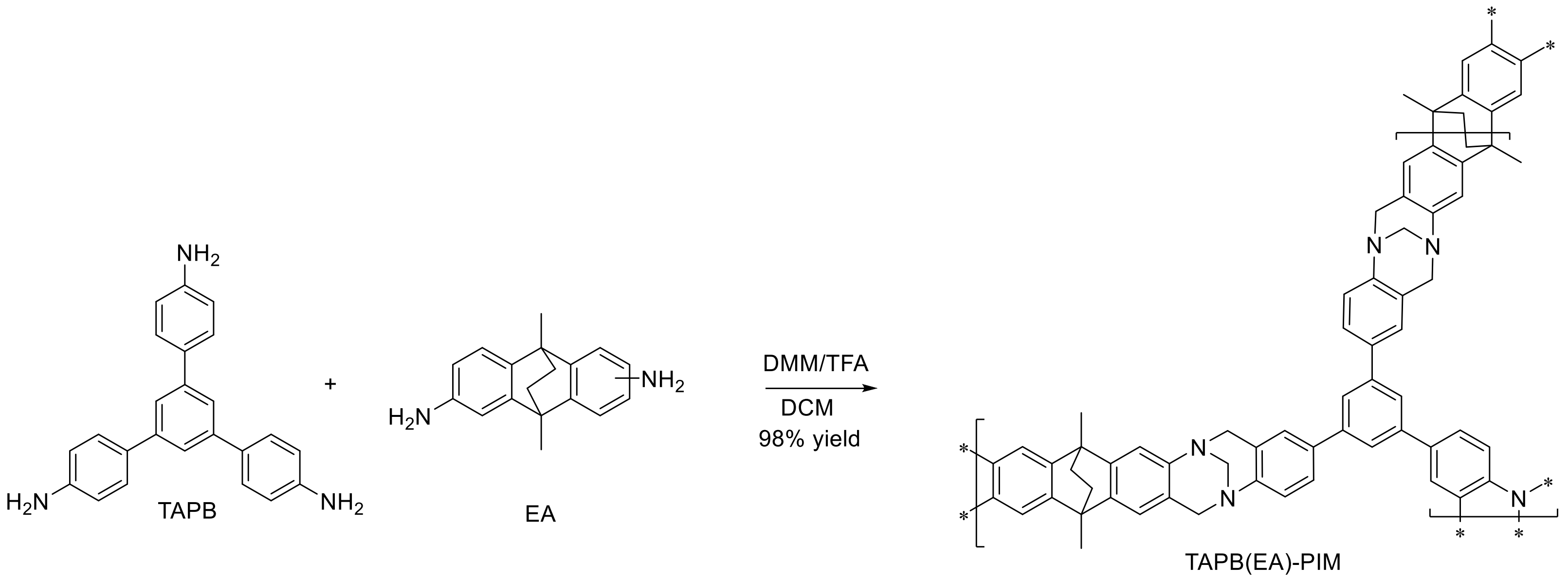


Tris(4-aminophenyl)benzene (TAPB) (0.40 g, 1.14 mmol), EA (0.45 g, 1.70 mmol) and dimethoxymethane (1.0 mL, 11.3 mmol) were stirred in DCM (10 mL), followed by dropwise addition of TFA (4.0 mL, 52 mmol) to yield a pale-yellow solid (0.75 g, 98% yield).
BET ($CO_2$, 273 K) = 530 $m^2 g^{-1}$, total pore volume =0.14 (at $P/P_0$ ~ 0.98). TGA: initial mass loss at 440 °C. FT-IR ν max ($cm^{-1}$) 2930, 1670, 1590, 1495, 1454, 1320, 1203, 1070, 990, 833. $^{13}$C NMR SS (101 MHz) δ 159.2, 145.3, 124.6, 117.4, 67.2, 59.2, 40.7, 35.9, 17.4, 2.1.

**TAPB(TAT)-PIM**

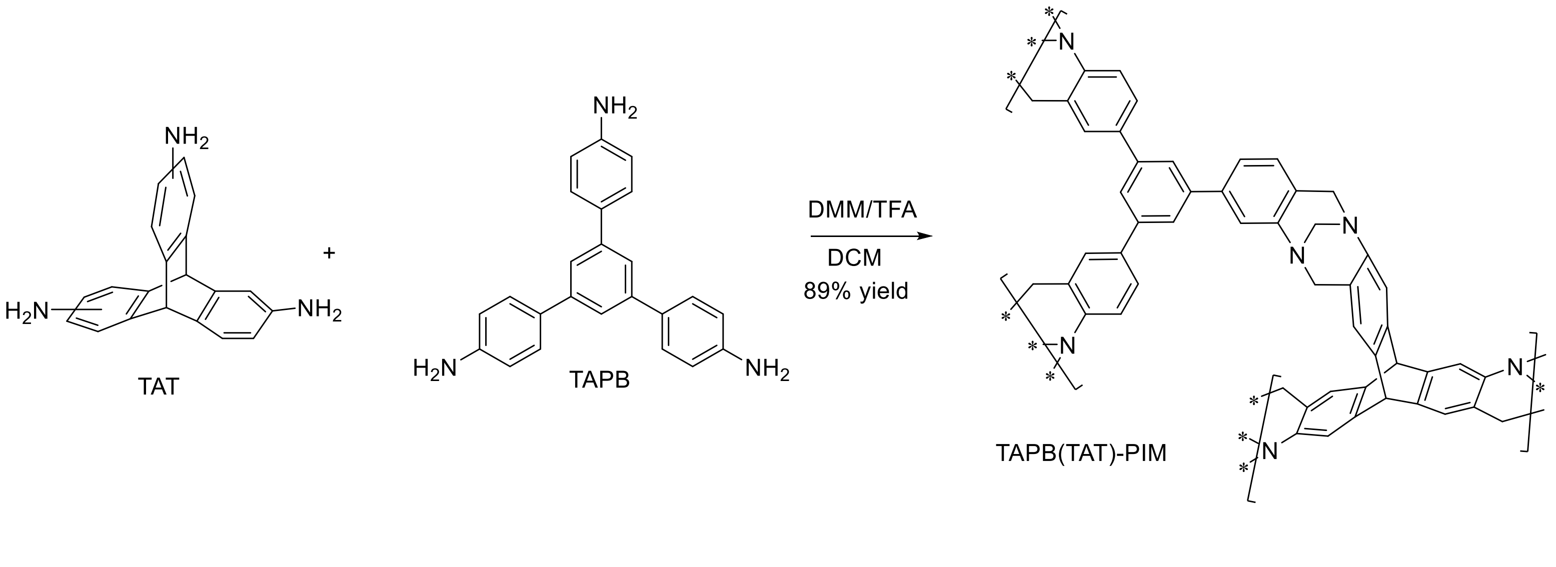

Tris(4-aminophenyl)benzene (TAPB) (0.47 g, 1.34 mmol), triaminotriptycene (TAT) (0.4 g, 1.34 mmol) and dimethoxymethane (0.83 ml, 9.38 mmol) were stirred in DCM (10 mL), followed by dropwise addition of TFA (3.5 mL, 45 mmol) to yield a brown solid (0.91 g ,89%).
BET ($CO_2$, 273 K) = 545 $m^2$ $g^{-1}$, total pore volume = 0.12 (at $P/P_0$ ~ 0.99). TGA: initial mass loss at 440 °C. FT-IR ν max ($cm^{-1}$) 3566, 2199, 1608, 1508, 822. $^{13}C$ NMR SS (101 MHz) δ 160.5, 144.3, 124.6, 66.8, 58.9, 53.0, 28.3, 20.3, 2.2.

**TAT(Tol)-PIM**

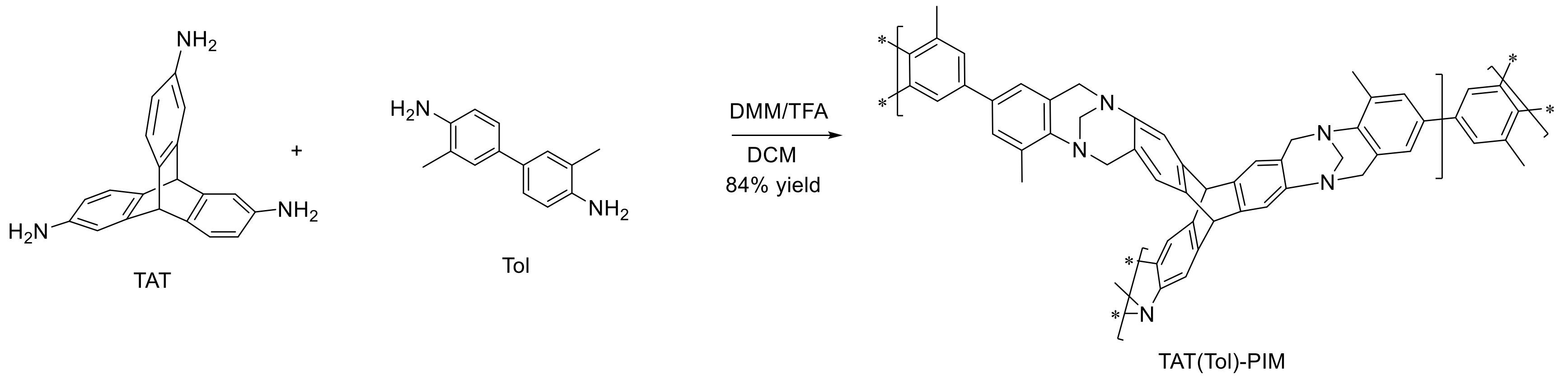


Triaminotriptycene (TAT) (0.53 g, 1.77 mmol), tolidine (Tol) (0.56 g, 2.66 mmol) and dimethoxymethane (1.09 ml, 12.3 mmol) were stirred in DCM (8 mL), followed by dropwise addition of TFA (4 mL, 52.3 mmol) to yield a brown solid (0.89 g ,84%).
BET ($CO_2$, 273 K) = 430 $m^2$ $g^{-1}$, total pore volume = 0.07 (at $P/P_0$ ~ 0.99). TGA: initial mass loss at 440 °C. FT-IR ν max ($cm^{-1}$) 3676, 2988, 2902, 1409, 1251, 1066, 892. $^{13}C$ NMR SS (101 MHz) δ 161.9, 144.8, 132.1, 127.7, 123.7, 111.6, 67.5, 53.9, 33.3, 16.6, 2.1.

**TAT(A1)-PIM**

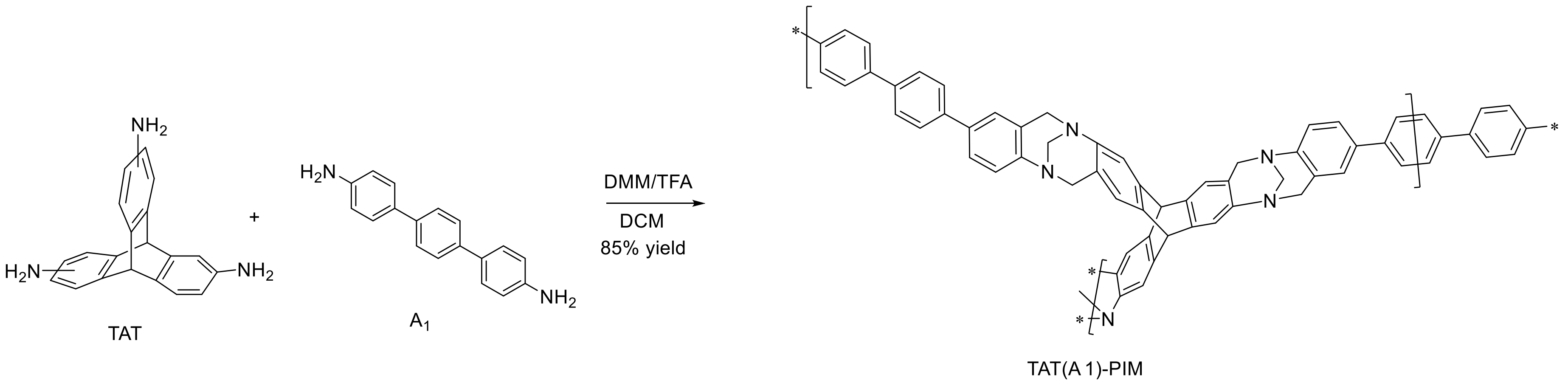


Triaminotriptycene (TAT) (0.50 g, 1.67 mmol), A1 (0.65 g, 2.50 mmol) and dimethoxymethane (1.2 mL, 13.6 mmol) were stirred in DCM (10 mL), followed by dropwise addition of TFA (4.5 mL, 58.8 mmol) to yield a red-brown solid (0.85 g, 85% yield).
BET ($CO_2$, 273 K) = 520 $m^2$ $g^{-1}$, total pore volume =0.12 (at $P/P_0$ ~ 0.98). TGA: initial mass loss at 440 °C. FT-IR ν max ($cm^{-1}$) 2925, 1664, 1610, 1480, 1205, 1070, 930, 820. $^{13}C$ NMR SS (101 MHz) δ 156.7, 144.9, 140.7, 125.6, 67.0, 58.9, 52.9, 29.8, 23.5, 1.1.

**TAT(A2)-PIM**

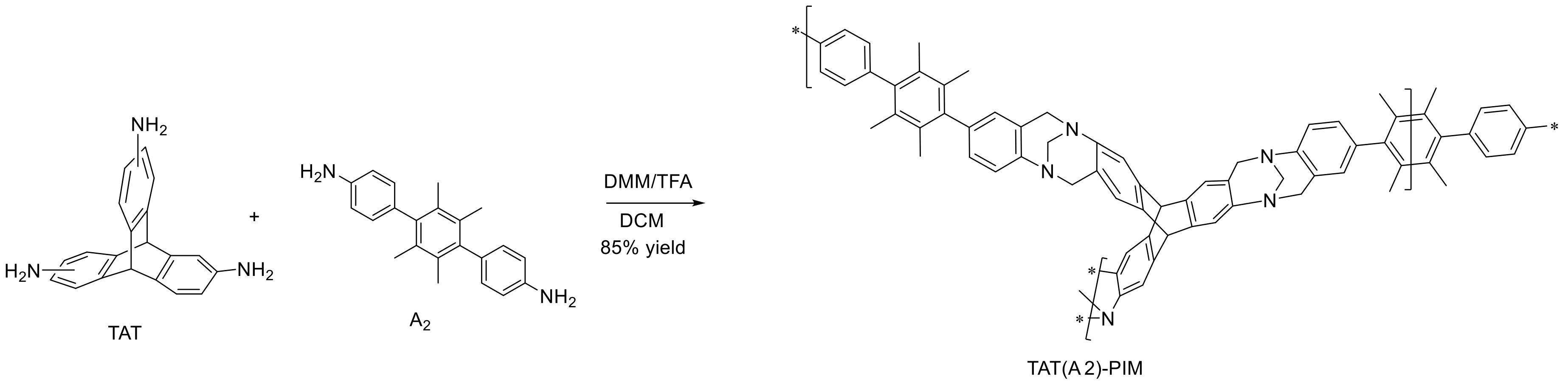


Triaminotriptycene (TAT) (0.32 g, 1.07 mmol), A2 (0.51 g, 1.6 mmol) and dimethoxymethane (0.8 mL, 9 mmol) were stirred in DCM (10 mL), followed by dropwise addition of TFA (3.0 mL, 39.2 mmol) to yield a pale orange solid (0.6 g, 86% yield).

BET ($CO_2$, 273 K) = 520 $m^2$ $g^{-1}$, total pore volume =0.15 (at $P/P_0$ ~ 0.98). TGA: initial mass loss at 440 °C. FT-IR ν max ($cm^{-1}$) 2960, 1613, 1460, 1208, 1080, 925, 825. $^{13}C$ NMR SS (101 MHz) δ 158.5, 145.4, 140.9, 131.3, 123.2, 67.3, 59.3, 53.5, 25.5, 17.1, 1.7.

**TAT(A3)-PIM**

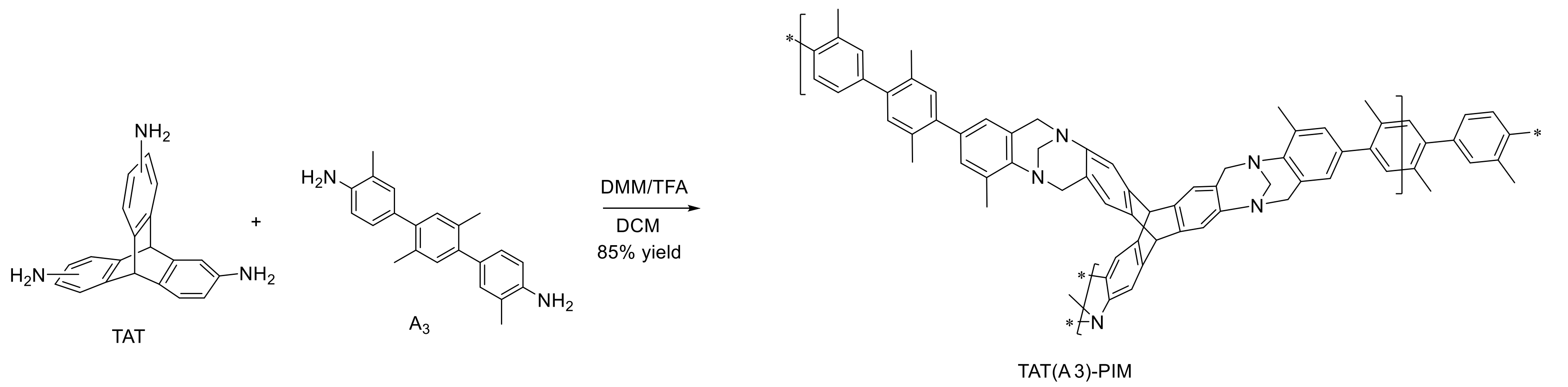


Triaminotriptycene (TAT) (0.36 g, 1.20 mmol), A3 (0.57 g, 1.8 mmol) and dimethoxymethane (0.8 mL, 9 mmol) were stirred in DCM (10 mL), followed by dropwise addition of TFA (3.5 mL, 46 mmol) to yield a brown solid (0.7 g, 87% yield).
BET ($CO_2$, 273 K) = 500 $m^2$ $g^{-1}$, total pore volume =0.12 (at $P/P_0$ ~ 0.98). TGA: initial mass loss at 440 °C. FT-IR ν max ($cm^{-1}$) 2970, 1613, 1465, 1210, 1070, 930, 870. $^{13}C$ NMR SS (101 MHz) δ 162.1, 144.7, 141.0, 131.6, 124.0, 67.5, 53.7, 39.3, 17.6, 7.4, 3.1.

**TAPBext(Tol)-PIM**

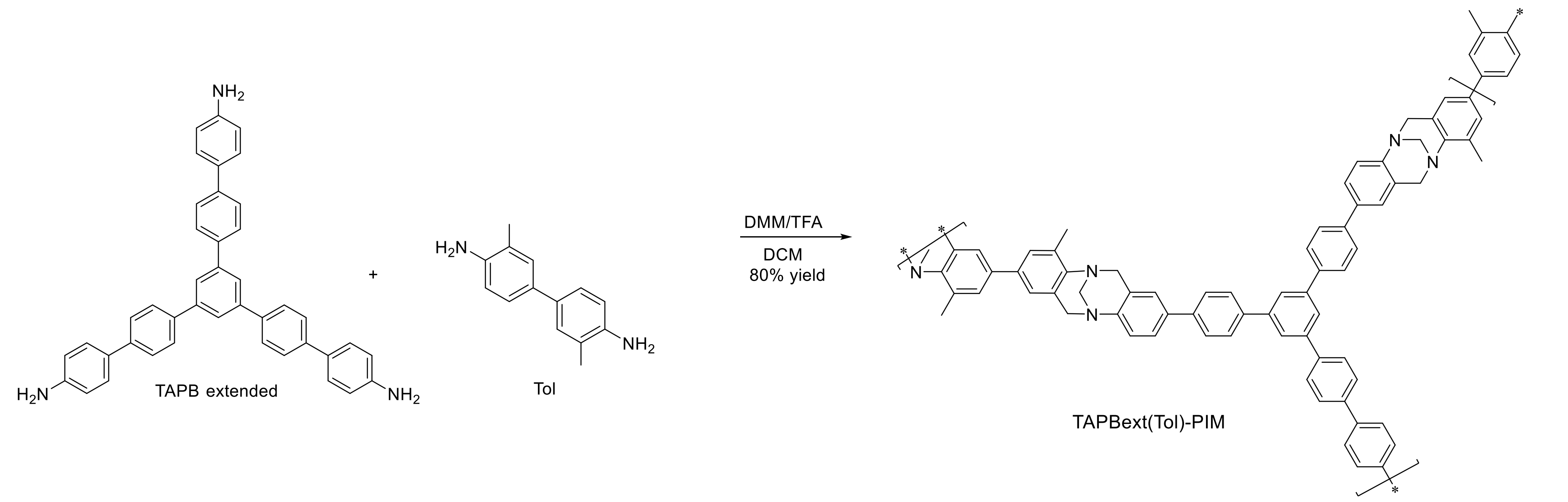


Extended Tris(4-aminophenyl)benzene (TAPBext) (0.5 g, 0.86 mmol), tolidine (Tol) (0.27 g, 1.29 mmol) and dimethoxymethane (0.72 mL, 8 mmol) were stirred in DCM (6 mL), followed by dropwise addition of TFA (3.9 mL, 51 mmol) to yield a pale-yellow solid (0.76 g, 80% yield).
BET ($CO_2$, 273 K) = 395 $m^2$ $g^{-1}$, total pore volume = 0.07 (at $P/P_0$ ~ 0.98). TGA: initial mass loss at 420 °C. FT-IR ν max ($cm^{-1}$) 3000, 1669, 1596, 1487, 1206, 937, 818, 520. $^{13}C$ NMR SS (101 MHz) δ 159.9, 147.5, 139.4, 127.2, 67.6, 55.8, 42.0, 26.0, 16.2, 3.0.

**TAPBext(A1)-PIM**

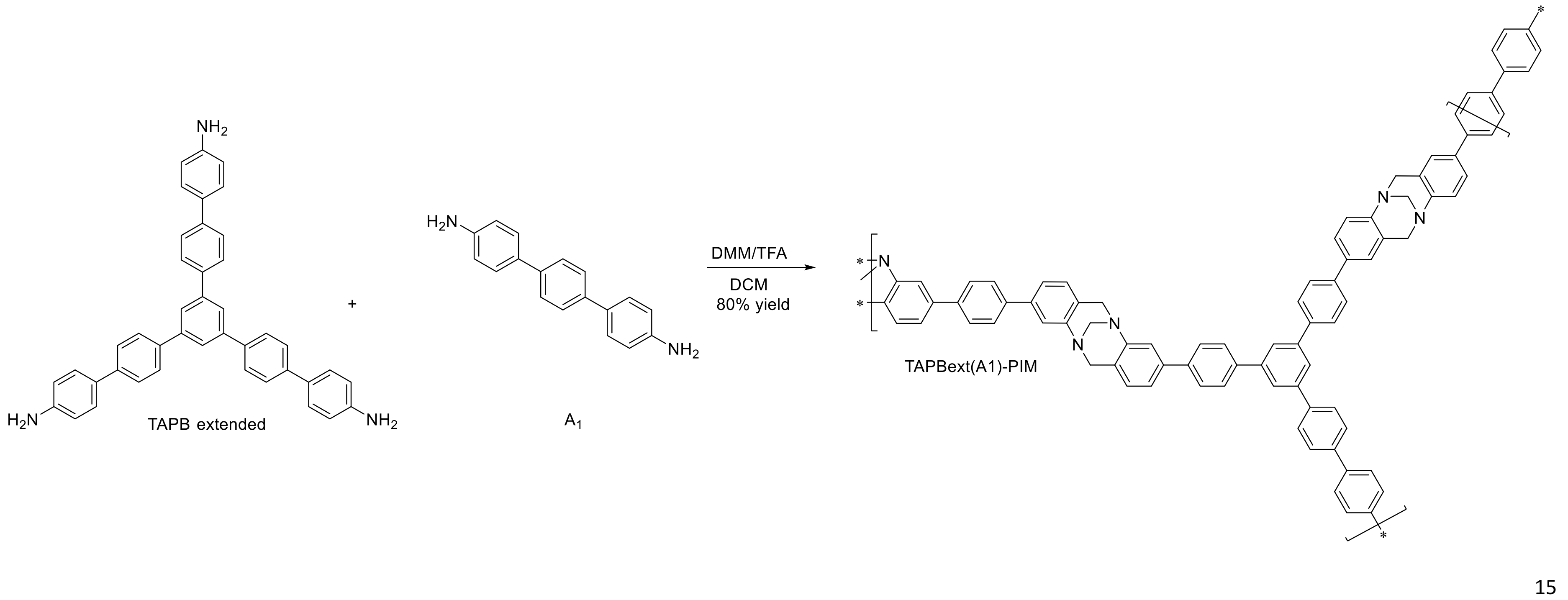

Extended Tris(4-aminophenyl)benzene (TAPBext) (0.8 g, 1.4 mmol), A1 (0.54 g, 2.07 mmol) and dimethoxymethane (1.0 mL, 11.3 mmol) were stirred in DCM (10 mL), followed by dropwise addition of TFA (4.0 mL, 52.3 mmol) to yield a pale-yellow solid (1.0 g, 80% yield).
BET ($CO_2$, 273 K) = 250 $m^2$ $g^{-1}$, total pore volume =0.06 (at $P/P_0$ ~ 0.98). TGA: initial mass loss at 430 °C. FT-IR ν max ($cm^{-1}$) 2980, 1680, 1600, 1490, 1203, 1070, 940, 812. $^{13}C$ NMR SS (101 MHz) δ 162.1, 144.7, 141.0, 131.6, 124.0, 67.5, 53.7, 39.3, 17.6, 7.4, 3.1.

## 4. General catalysis test:

Malononitrile: benzaldehyde (3:1) solvent free
A glass vial was charged with a mixture of benzaldehyde (15 mmol) and malononitrile (5 mmol), then the catalyst was added (1 mol%), and the reaction mixture was stirred at room temperature for 2 h. Fractions (10 microliters) were removed each 10 min and analysed by $^1H$ NMR.

Malononitrile: benzaldehyde (3:1) solvent
A glass vial was charged with a mixture of benzaldehyde (15 mmol), malononitrile (5 mmol) and 2 mL of solvent (ethanol or DCM). Then, the catalyst (1 mol%) was added and the reaction mixture was stirred at room temperature for 2 h. Fractions (10 microliters) were removed each 10 min and analysed by $^1H$ NMR.

Malononitrile: benzaldehyde (1:1) solvent
A glass vial was charged with a mixture of benzaldehyde (5 mmol), malononitrile (5 mmol) and 2 mL of solvent (ethanol or DCM). Then, the catalyst (1mol%) was added and the reaction mixture was stirred in at room temperature for 2 h. Fractions (10 microliters) were removed each 10 min and analysed by $^1H$ NMR.

## 5. Tables

**Table ESI 1.** Combination of the different monomers that form TB-polymers and co-polymers.

| Polymer | Monomer A | Monomer B | BET[a] ($m^2$ $g^{-1}$) | TGA ($^0C$) | Polymer | Monomer A | Monomer B | BET ($m^2$ $g^{-1}$) | TGA ($^0C$) |
|---|---|---|---|---|---|---|---|---|---|
| TAPB-PIM | 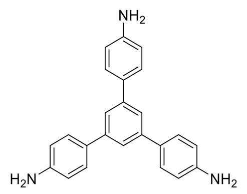 | | 500 | 440 | TAPB(TAT)-PIM | 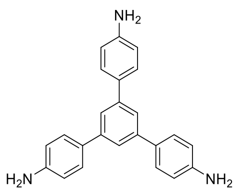 | 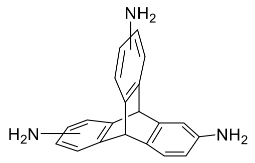 | 545 | 440 |
| TAPBext-PIM | 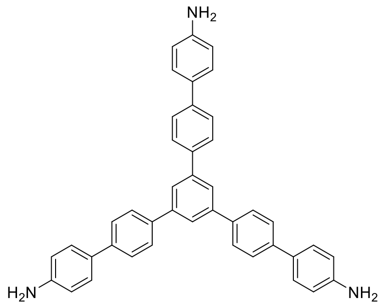 | | 350 | 495 | TAT(Tol)-PIM | 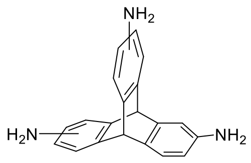 | 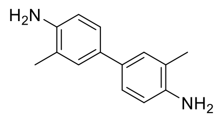 | 430 | 440 |
| TAPB(Tol)-PIM | 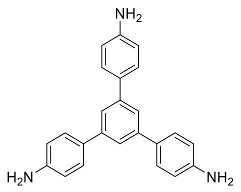 | 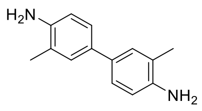 | 360 | 440 | TAT(A1)-PIM | 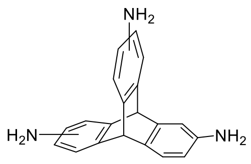 | 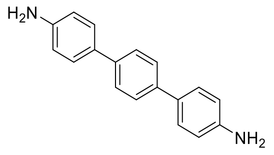 | 520 | 430 |
| TAPB(A1)-PIM | 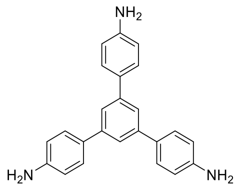 | 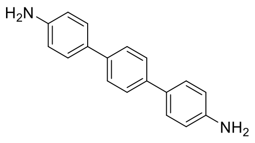 | 470 | 440 | TAT(A2)-PIM | 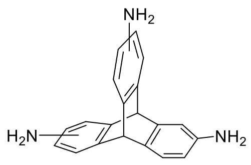 | 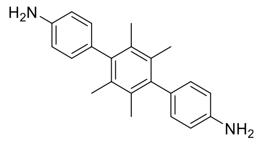 | 600 | 440 |
| TAPB(A2)-PIM | 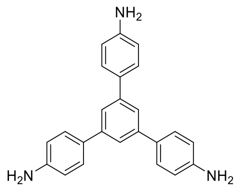 | 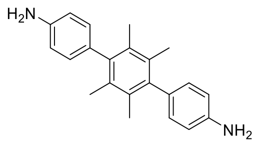 | 370 | 440 | TAT(A3)-PIM | 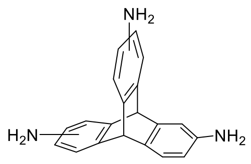 | 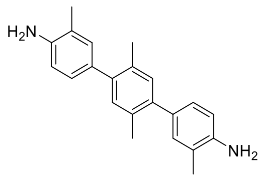 | 500 | 450 |
| TAPB(A3)-PIM | 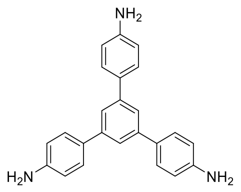 | 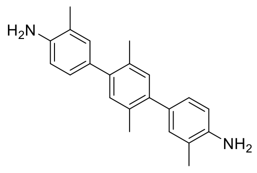 | 350 | 430 | TAPBext (Tol)-PIM | 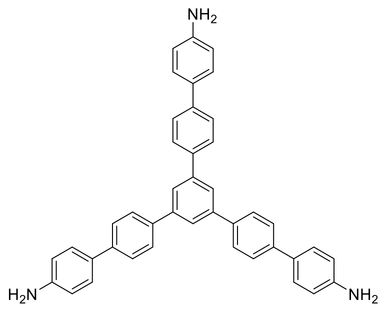 | 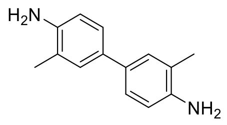 | 395 | 420 |
| TAPB(EA)-PIM | 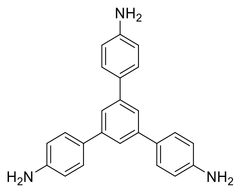 | 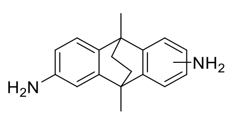 | 530 | 260[b] + 440 | TAPBext(A1)-PIM | 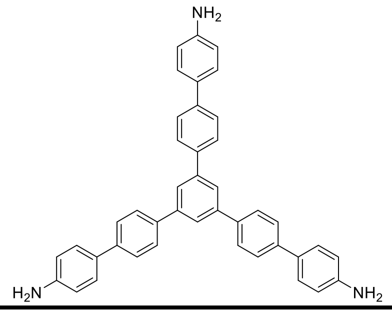 | 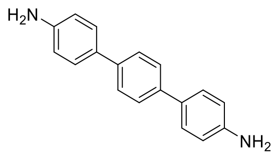 | 250 | 430 |

[a] calculated from $CO_2$ adsorption at 273K

[b] the first decomposition temperature refers to the retro Diels-Alder step that we used to assess the correct stoichiometry of the two co-monomers (see also **Figure ESI 3**).

**Table ESI 2**. Knoevenagel condensation reaction between benzaldehyde (**A**) and malononitrile (**B**) in the presence of a solvent. [a]

| Catalyst | A:B | Solvent | Conversion at *x* time [b] | | | | | | TON[c] | TOF [d] |
|---|---|---|---|---|---|---|---|---|---|---|
| | | | **10** | **20** | **40** | **60** | **80** | **120** | | |
| TAPB-PIM | 3:1 | DCM | 35 | 45 | 61 | 78 | 86 | 97 | 64 | 3.2 |
| | 3:1 | EtOH | 81 | 93 | 100 | - | - | - | 93 | 4.7 |
| | 1:1 | EtOH | 64 | 95 | 100 | - | - | - | 95 | 4.8 |
| TAPBext-PIM | 3:1 | DCM | 72 | 84 | 93 | 98 | 100 | - | 84 | 4.2 |
| | 3:1 | EtOH | 80 | **100** | - | - | - | - | **100** | **5** |
| | 1:1 | EtOH | 86 | **100** | - | - | - | - | **100** | **5** |
| TAPB(Tol)-PIM | 3:1 | DCM | 32 | 51 | 70 | 81 | 87 | 93 | 51 | 2.6 |
| | 3:1 | EtOH | 54 | 79 | 95 | 100 | - | - | 79 | 4 |
| | 1:1 | EtOH | 40 | 60 | 88 | 100 | - | - | 60 | 3 |
| TAPB(EA)-PIM | 3:1 | DCM | 38 | 66 | 79 | 92 | 95 | 100 | 66 | 3.3 |
| | 3:1 | EtOH | 84 | **100** | - | - | - | - | **100** | **5** |
| | 1:1 | EtOH | 64 | 87 | **100** | - | - | - | 87 | 4.4 |
| TAPBext (Tol)-PIM | 3:1 | DCM | 50 | 62 | 74 | 87 | 92 | 100 | 62 | 3.1 |
| | 3:1 | EtOH | 70 | 84 | 96 | 100 | - | - | 84 | 4.2 |
| | 1:1 | EtOH | 63 | 78 | 100 | - | - | - | 78 | 3.9 |
| TAPBext(A1)-PIM | 3:1 | DCM | 46 | 70 | 86 | 100 | - | - | 70 | 3.5 |
| | 3:1 | EtOH | 85 | **100** | - | - | - | - | **100** | **5** |
| | 1:1 | EtOH | 88 | **100** | - | - | - | - | **100** | **5** |

[a] Reactions conditions: A mixture of benzaldehyde (15 mmol or 5 mmol), malononitrile (5 mmol), solvent (2 mL) and 1 mol% catalyst was stirred at room temperature for 2 h at 25 $^{0}$C. [b] Conversion of malononitrile was determined by NMR. [c] Turnover number (TON) at 20 min calculated from no. of moles of malononitrile consumed per mole equivalents of TB catalyst. [d] Turnover frequency (TOF) calculated from turnover number per minute.

**Table ESI 3.** Catalysis results with substituted benzaldehydes under solvent-free conditions

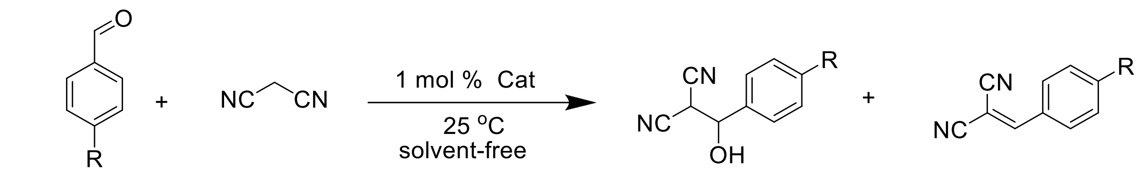


| Polymer | R group | Time (minutes) | | | | | | |
|---|---|---|---|---|---|---|---|---|
| | | **20** | **40** | **60** | **80** | **100** | **120** | **180** |
| **PIM-TB-Trip 1** | H | 40 | 63 | 74 | 82 | 86 | 93 | |
| | tBu | 10 | 23 | 24 | 29 | 35 | 36 | 48 |
| **TAPB-PIM** | H | 64 | 85 | 94 | **97** | **100** | - | |
| | F | 47 | 66 | 80 | 87 | 94 | **98** | |
| | MeO | 42 | 56 | 70 | | | | |
| | tBu | 19 | 33 | 40 | 48 | 52 | 57 | 69 |
| **TAPB(Tol)-PIM** | H | 63 | 84 | 92 | **96** | **98** | **100** | |
| | F | 21 | 33 | 42 | 50 | 60 | 65 | |
| | MeO | 18 | 20 | 22 | 26 | 30 | 32 | |
| | tBu | 10 | 11 | 11 | 12 | 14 | 15 | 17 |
| **TAPBext-PIM** | H | 78 | 94 | **98** | **99** | **100** | | |
| | F | 24 | 31 | 40 | 50 | 55 | 60 | |
| | MeO | 18 | 20 | 22 | 26 | 30 | 32 | |
| | tBu | 10 | 13 | 16 | 18 | 20 | 21 | 25 |
| **TAPB(A1)-PIM** | H | 86 | **98** | **100** | - | | | |
| | F | 70 | 92 | **100** | | | | |
| | MeO | 69 | | | | | | |
| | tBu | 2 | 2 | 4 | 6 | 9 | 9 | 15 |
| **TAPBext(Tol)-PIM** | H | 83 | 92 | **96** | **98** | **99** | **100** | |
| | F | 20 | 40 | 52 | 62 | 71 | 75 | |
| | MeO | 14 | 20 | | | 50 | 55 | |
| | tBu | 2 | 4 | 4 | 6 | 6 | 8 | 10 |
| **TAPBext(A1)-PIM** | H | 95 | **100** | | | | | |
| | F | 80 | **99** | | | | | |
| | MeO | 70 | | | | | | |
| | tBu | 4 | 6 | 7 | 9 | 12 | 12 | 17 |

**Table ESI 4.** Catalysis results with substituted benzaldehydes using EtOH as solvent.

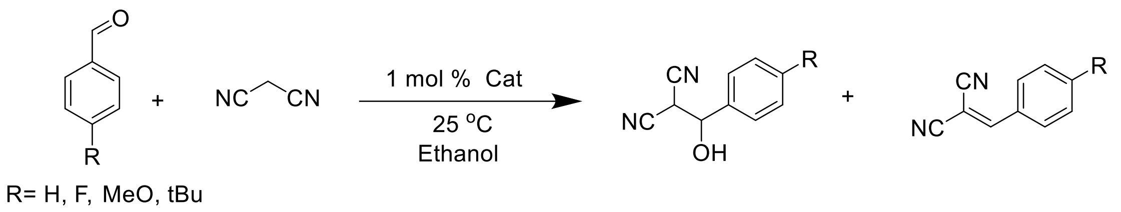


| Polymer | R group | Time (minutes) | | | | | | |
|---|---|---|---|---|---|---|---|---|
| | | **20** | **40** | **60** | **80** | **100** | **120** | **180** |
| **TAT-PIM** | H | 67 | **95** | | | | | |
| | tBu | 32 | 44 | 52 | 63 | 67 | 70 | 81 |
| **TAPB-PIM** | H | **100** | | | | | | |
| | F | 80 | **100** | | | | | |
| | MeO | 36 | | | | | | |
| | tBu | 31 | 44 | 55 | 65 | 70 | 75 | 81 |
| **TAPB(Tol)-PIM** | H | 60 | 88 | 100 | | | | |
| | F | 40 | 70 | 100 | | | | |
| | MeO | 17 | 28 | 37 | 42 | 50 | 54 | |
| | tBu | 33 | 44 | 56 | 62 | 74 | 86 | 89 |
| **TAPBext-PIM** | H | 100 | | | | | | |
| | F | 42 | 52 | 63 | 75 | 80 | 85 | |
| | MeO | 19 | 26 | 31 | 34 | 38 | 43 | |
| | tBu | 33 | 40 | 47 | 50 | 55 | 58 | 67 |
| **TAPB(A1)-PIM** | H | 87 | **100** | | | | | |
| | F | 93 | 100 | | | | | |
| | MeO | 70 | | | | | | |
| | tBu | 43 | 57 | 65 | 72 | 75 | 78 | 95 |
| **TAPBext(Tol)-PIM** | H | 78 | 100 | | | | | |
| | F | 21 | 40 | 57 | 75 | 81 | 93 | |
| | MeO | 20 | 25 | 33 | 41 | | | |
| | tBu | 27 | 37 | 49 | 52 | 55 | 60 | 70 |
| **TAPBext(A1)-PIM** | H | 100 | | | | | | |
| | F | 93 | **100** | | | | | |
| | MeO | 20 | 25 | 33 | 41 | | | |
| | tBu | 45 | 60 | 70 | 75 | 80 | 85 | 95 |

**Table ESI 5**. Knoevenagel condensation reaction between tert-butylbenzaldehyde (A) and malononitrile (B). [a]

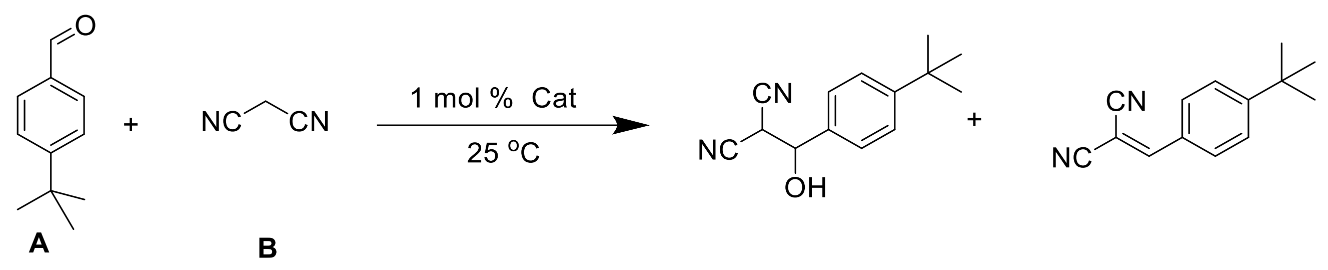


| Catalyst | A:B | Solvent | Conversion at *x* time [b] | | | | | | TON[c] | TOF [d] |
|---|---|---|---|---|---|---|---|---|---|---|
| | | | **20** | **60** | **80** | **100** | **120** | **180** | | |
| TAPB-PIM | 3:1 | - | 19 | 40 | 48 | 52 | 57 | 69 | 19 | 1.0 |
| | 3:1 | DCM | 36 | 52 | 57 | 65 | 73 | 80 | 36 | 1.8 |
| | 3:1 | EtOH | 40 | 77 | 84 | 92 | 96 | 98 | 40 | 2.0 |
| | 1:1 | EtOH | 31 | 55 | 65 | 70 | 75 | 81 | 31 | 1.6 |
| TAPB(A1)-PIM | 3:1 | - | 2 | 4 | 6 | 9 | 9 | 15 | 2 | 0.1 |
| | 3:1 | DCM | 50 | 72 | 78 | 84 | 90 | 96 | 50 | 2.5 |
| | 3:1 | EtOH | 26 | 61 | 68 | 80 | 83 | 98 | 26 | 1.3 |
| | 1:1 | EtOH | 43 | 65 | 72 | 75 | 78 | 95 | 43 | 2.2 |
| TAPBext(A1)-PIM | 3:1 | - | 4 | 7 | 9 | 12 | 12 | 17 | 4 | 0.2 |
| | 3:1 | DCM | 50 | 73 | 78 | 83 | 90 | 96 | 50 | 2.5 |
| | 3:1 | EtOH | 56 | 82 | 90 | 96 | 99 | **100** | 56 | **2.8** |
| | 1:1 | EtOH | 45 | 70 | 75 | 80 | 85 | 95 | 45 | 2.3 |

[a] Reactions conditions: A mixture of benzaldehyde (15 mmol or 5 mmol), malononitrile (5 mmol), solvent (2 mL) and 1 mol% catalyst was stirred at room temperature for 2 h at 25 $^0$C. [b] Conversion of malononitrile was determined by NMR. [c] Turnover number at 20 min calculated from no. of moles of malononitrile consumed per mole equivalents of TB catalyst. [d] Turnover frequency calculated from turnover number per minute.

**Table ESI 6.** Knoevenagel condensation reaction using larger benzaldehydes. [a]

| Catalyst | Aldehyde | Conversion at *x* time[b] | | | |
|---|---|---|---|---|---|
| | | **30** | **60** | **120** | **180** |
| TAPB-PIM | Benzaldehyde | 20 | 40 | 50 | 55 |
| | 4-Biphenylcarboxaldehyde | 17 | 29 | 44 | 50 |
| | 2-naphtaldehyde | 18 | 37 | 53 | 60 |
| | tBu-benzaldehyde | 18 | 29 | 38 | 40 |
| | 9-Anthracenecarboxaldehyde | 7 | 15 | 23 | 27 |
| TAPBext(A1)-PIM | Benzaldehyde | 30 | 45 | 73 | 80 |
| | 4-Biphenylcarboxaldehyde | 22 | 39 | 66 | 70 |
| | 2-naphtaldehyde | 30 | 46 | 74 | 77 |
| | tBu-benzaldehyde | 18 | 29 | 47 | 60 |
| | 9-Anthracenecarboxaldehyde | 15 | 26 | 47 | 57 |

[a] Reactions conditions: A mixture of aldehyde (3 mmol), malononitrile (3 mmol), DCM (3 mL) and 1 mol% catalyst was stirred at room temperature for 3 h at 25 $^0$C. [b] Conversion of malononitrile was determined by NMR.

**Table ESI 7.** Knoevenagel condensation reaction using larger benzaldehydes via homogeneous catalysis. [a]

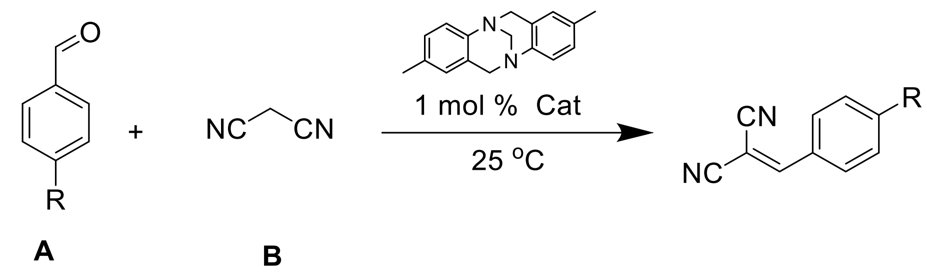


| A | A:B | Solvent | Conversion at *x* time [b] | | | |
|---|---|---|---|---|---|---|
| | | | **30** | **60** | **120** | **180** |
| tBu-benzaldehyde | 3:1 | Solvent free | 32 | 42 | 65 | 70 |
| | 1:1 | EtOH | 45 | 50 | 69 | 73 |
| | 1:1 [c] | DCM | 0 | 2 | 5 | 5 |
| tBu with TAPBext(A1)-PIM | 1:1 | EtOH | 56 | 70 | 85 | 95 |
| 9-Anthracenecarboxaldehyde [c] | 1:1 | DCM | 0 | 0 | 0 | 0 |

[a] Reactions conditions: A mixture of benzaldehyde (15 mmol or 5 mmol), malononitrile (5 mmol), solvent (2 mL) and 1 mol% catalyst was stirred at room temperature for 3 h at 25 $^0$C. [b] Conversion of malononitrile was determined by NMR. [c] Reaction conditions: benzaldehyde (3 mmol), malononitrile (3 mmol), DCM (3 mL) and 1 mol% catalyst.

## 6. Various figures

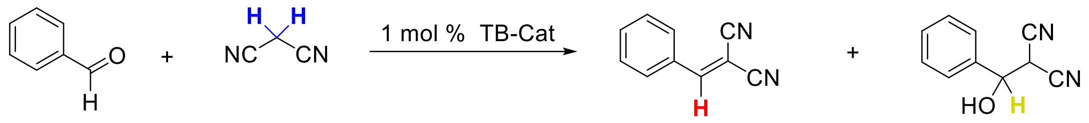


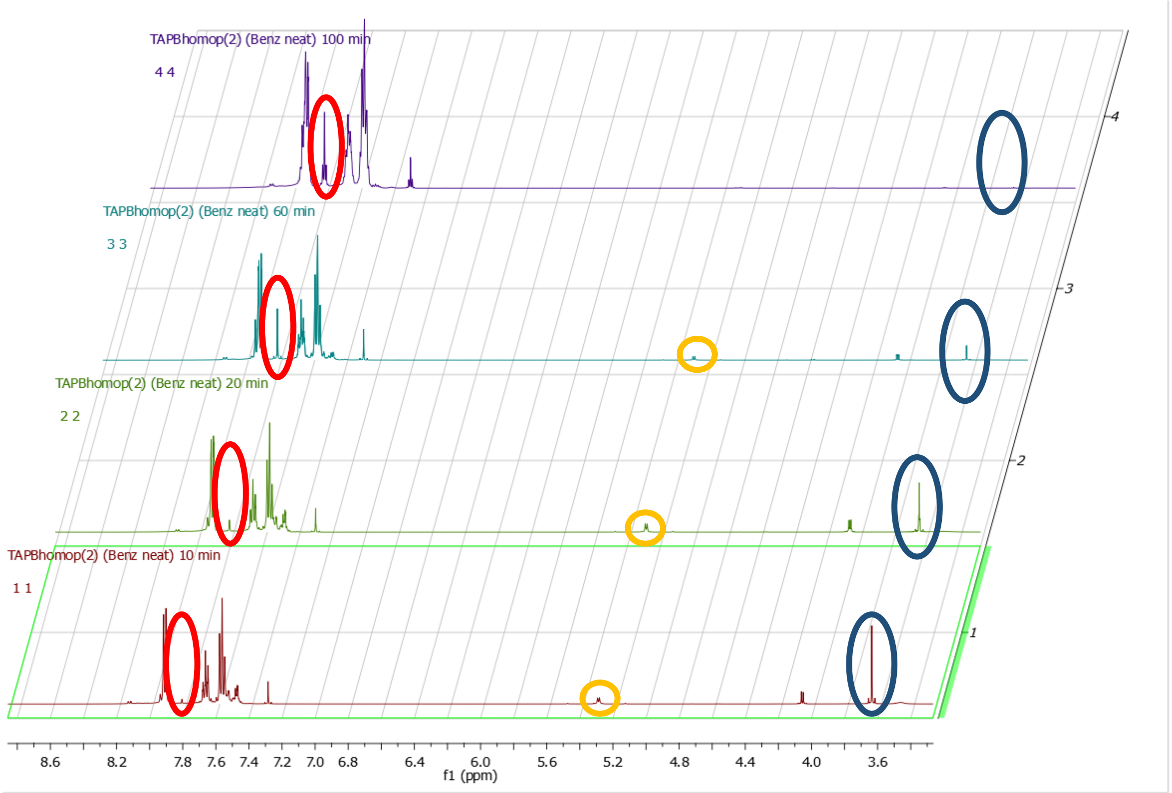


**Figure ESI 1.** $^{1}$HNMR of samples from the reaction between benzaldehyde vs. malononitrile (3:1) under solvent-free conditions using TAPB-PIM as catalyst.

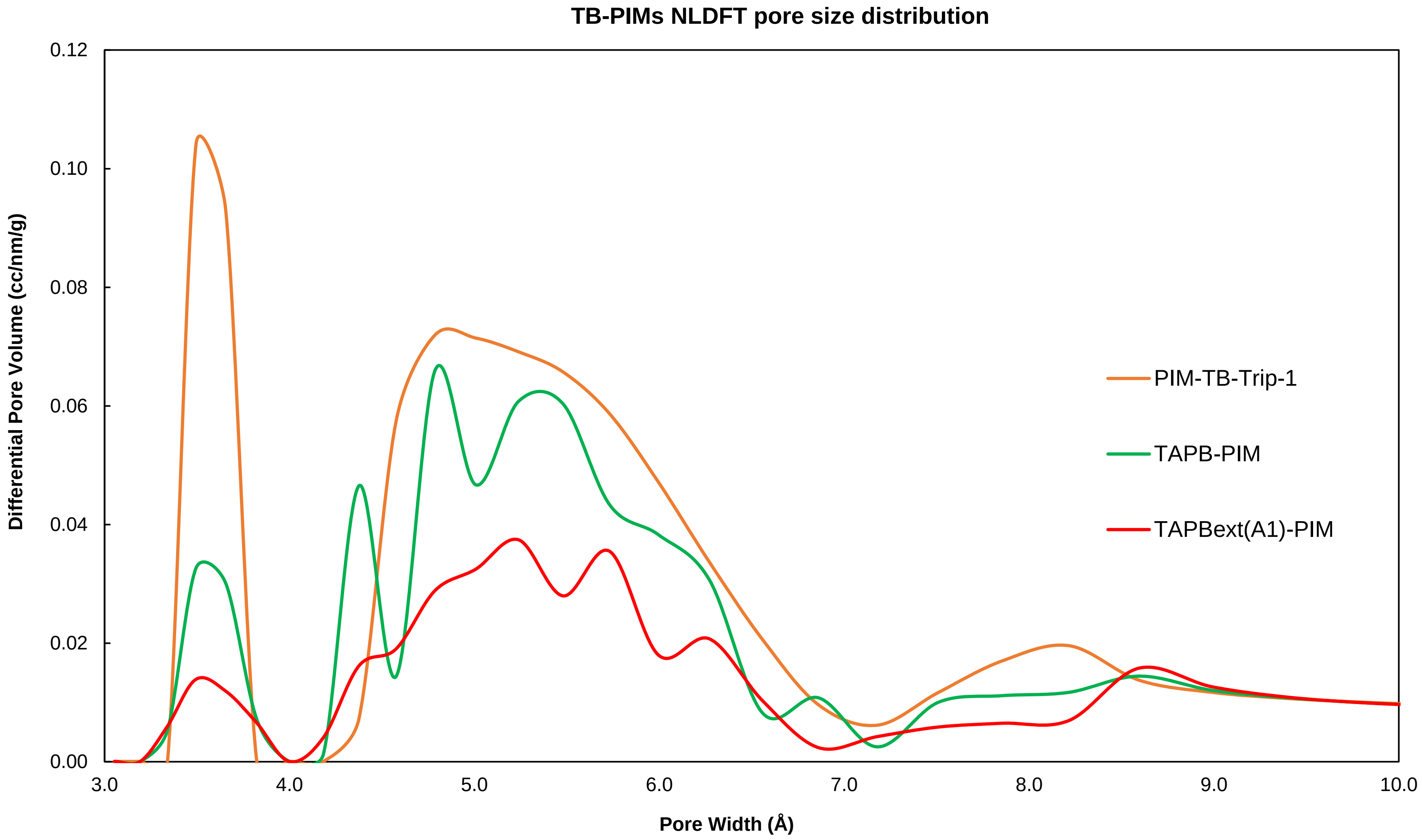


**Figure ESI 2.** Pore size distribution of some TB polymers, calculated from $CO_2$ adsorption at 273 K and by NLDFT.

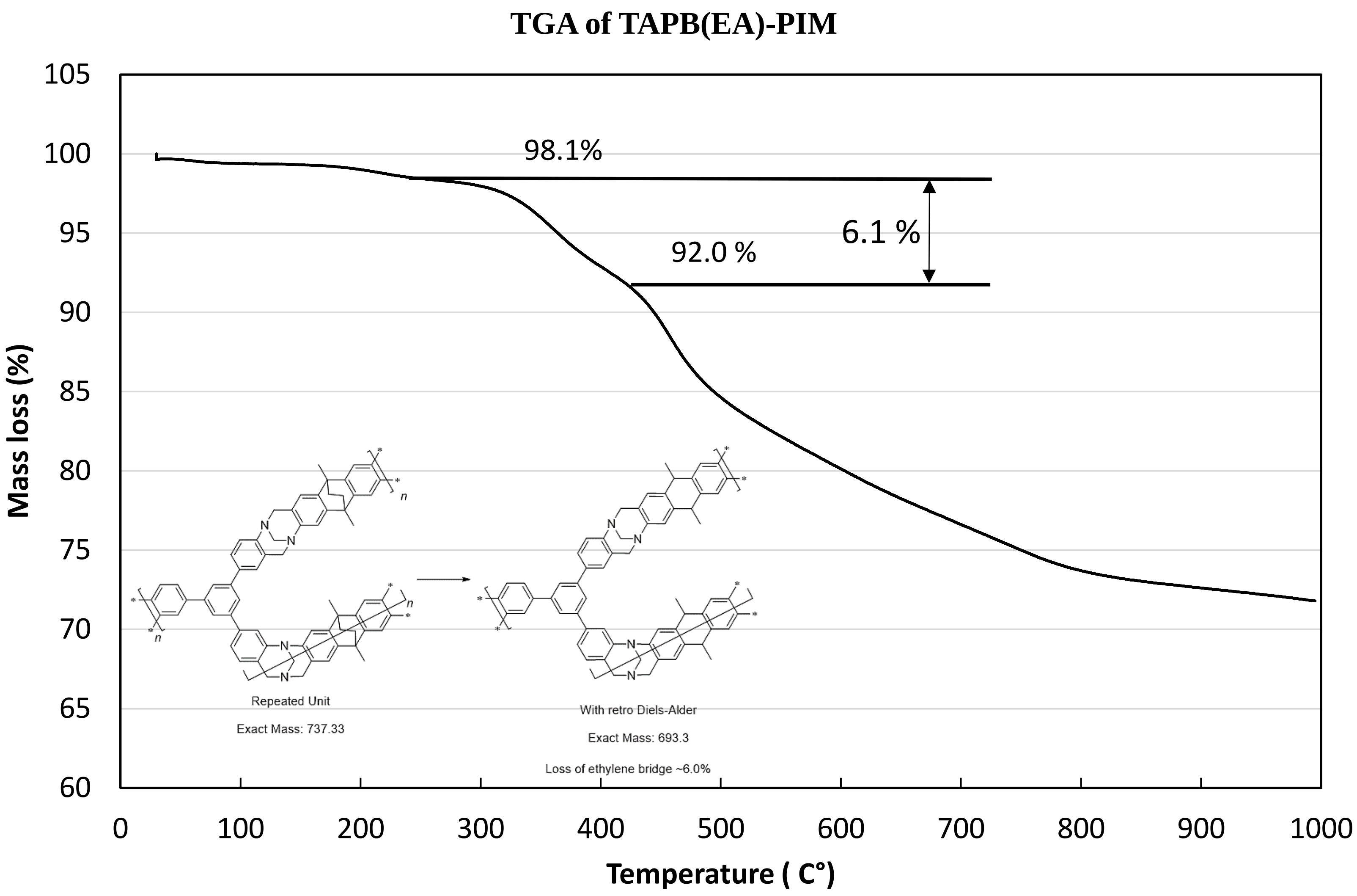


**Figure ESI 3.** TGA of TAPB(EA)-PIM. The loss of the ethylene bridge by retro Diels-Alder shows the 50:50 ratio between the two monomers.

## Recyclability test

A glass vial was charged with a mixture of 4-*tert*-butylbenzaldehyde (25 mmol), malononitrile (25 mmol) and 10 mL of ethanol. Then, **TAPB-ext(A1)-PIM** (1mol%) was added and the reaction mixture was stirred at room temperature for 3 h. After this period, the reaction was analysed by NMR and the catalyst was recovered from the reaction by simple filtration, refluxed in different solvents (acetone, DCM and methanol), dried in a vacuum oven (at 100 $^{0}$C for 20 h) and reused. This procedure was repeated for more six consecutive cycles.

The *t*Bu-derivative was separated and weighed, to be sure that the yield matched with the conversion seen by $^{1}$H NMR. To ensure that the structure of the polymer has not changed during the recycling tests, we occasionally repeated the physical characterisation (BET and FT-IR), finding that the physical properties were not affected.

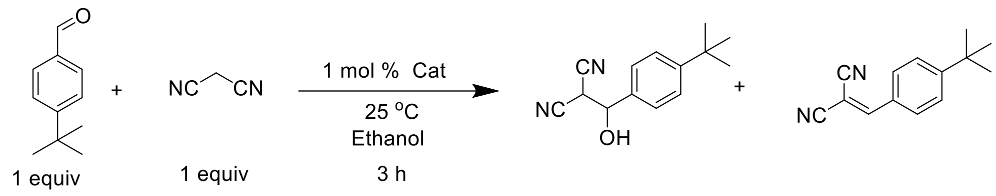


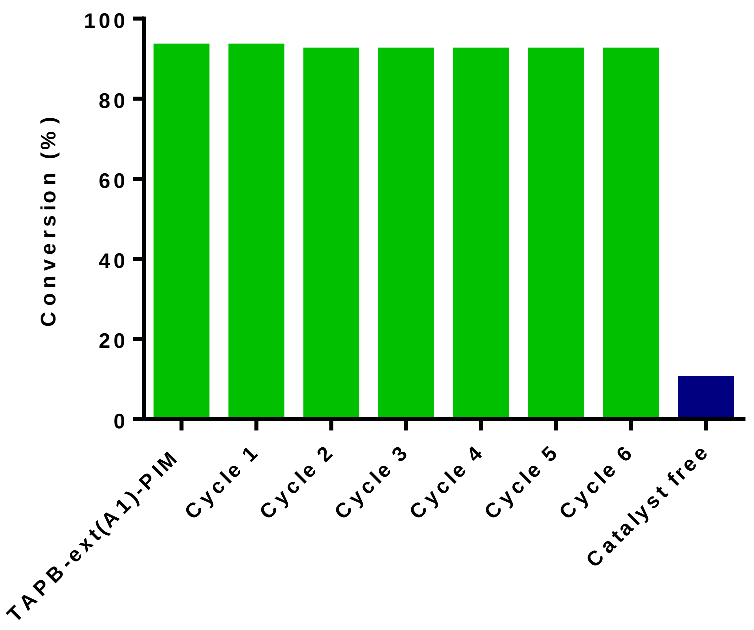


**Figure ESI 4.** Recyclability test of TAPBext(A1)-PIM after the Knoevenagel reaction using tert-butylbenzaldehyde : malononitrile 1:1 and ethanol as a solvent.

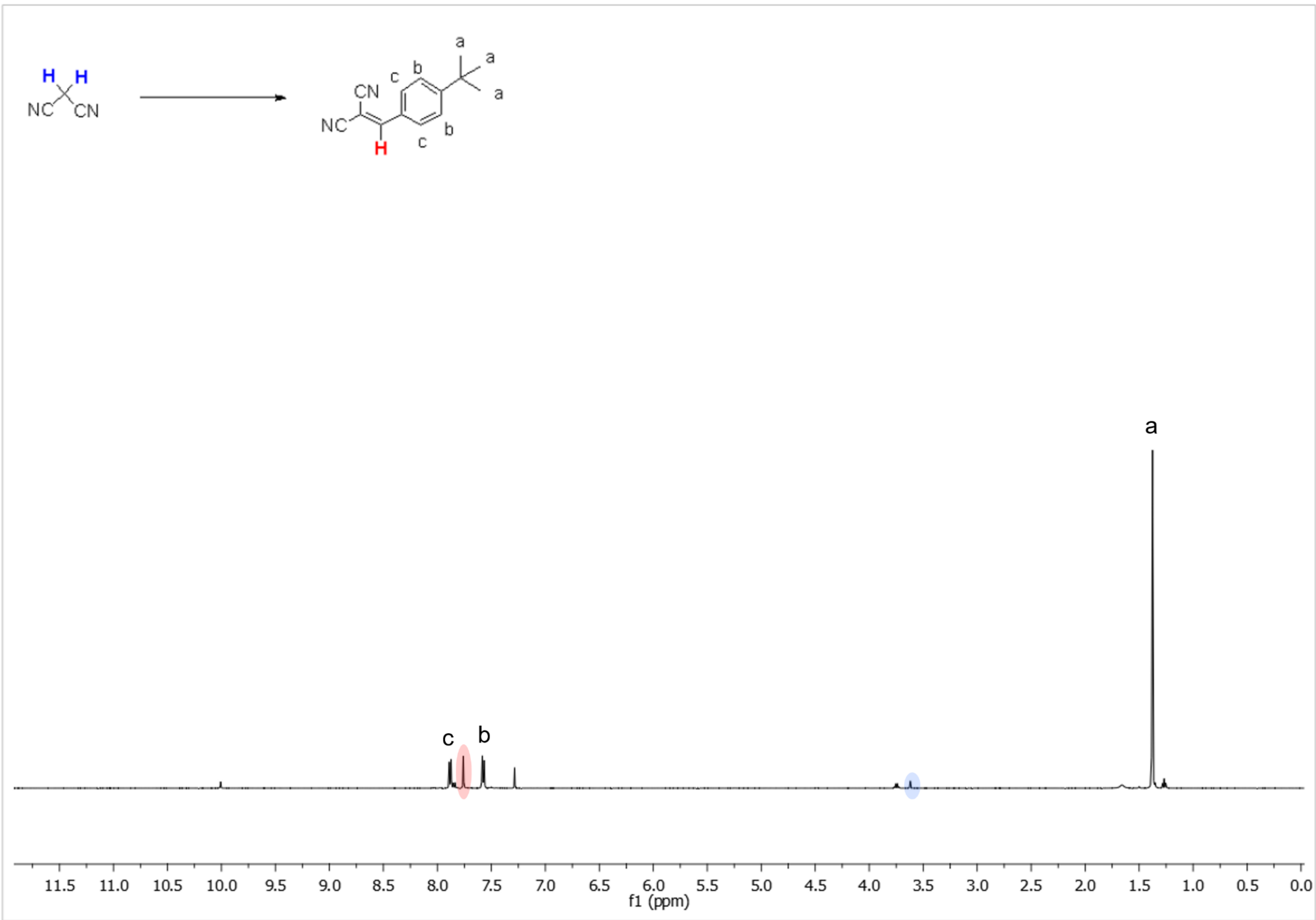


**Figure ESI 5.** $^{1}$HNMR of the reaction between tert-butylbenzaldehyde vs. malononitrile (1:1 in ethanol) using **TAPBext(A1)-PIM** as catalyst (after removing excess ethanol).

## 7. $^{13}$C Solid state NMR

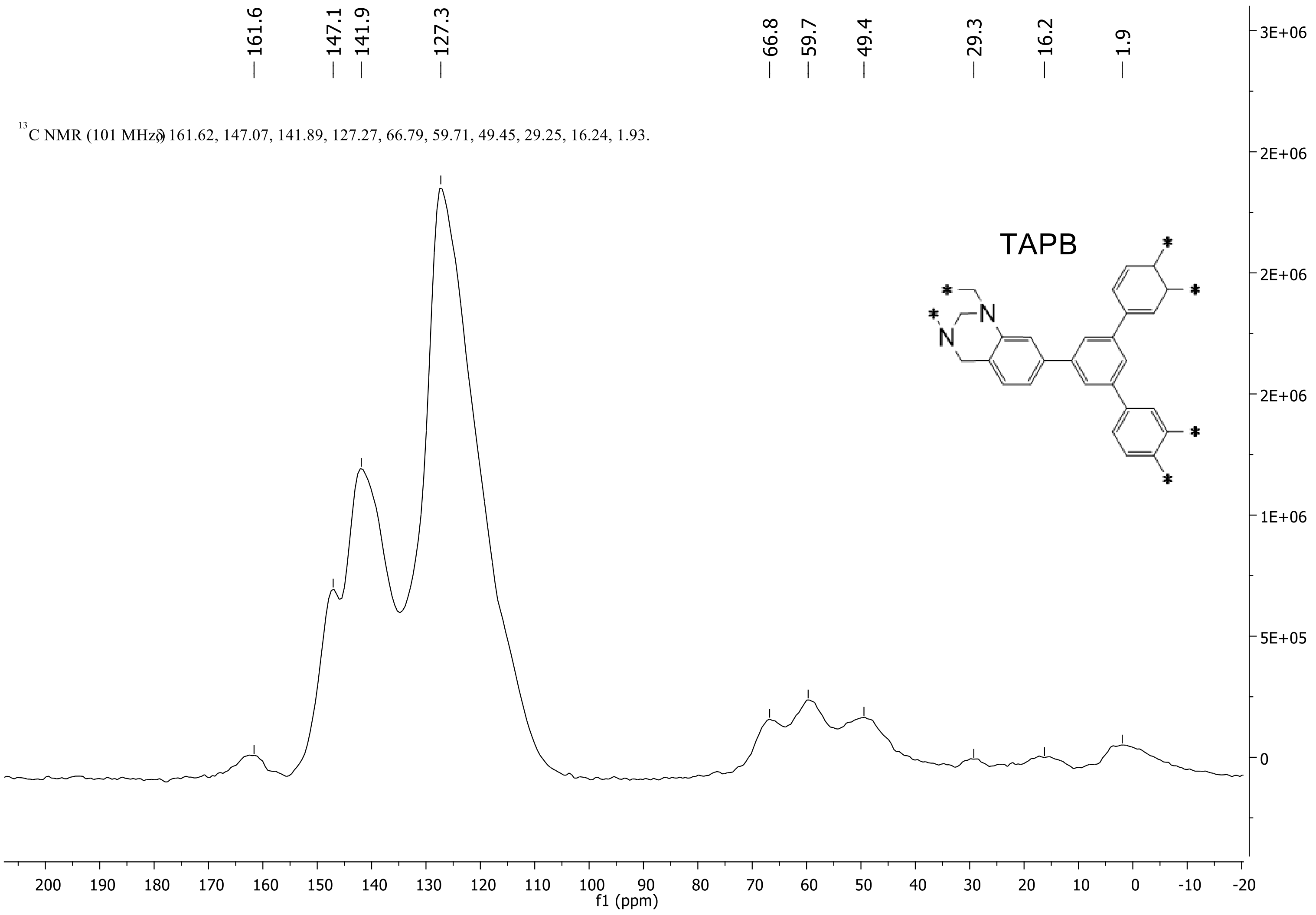

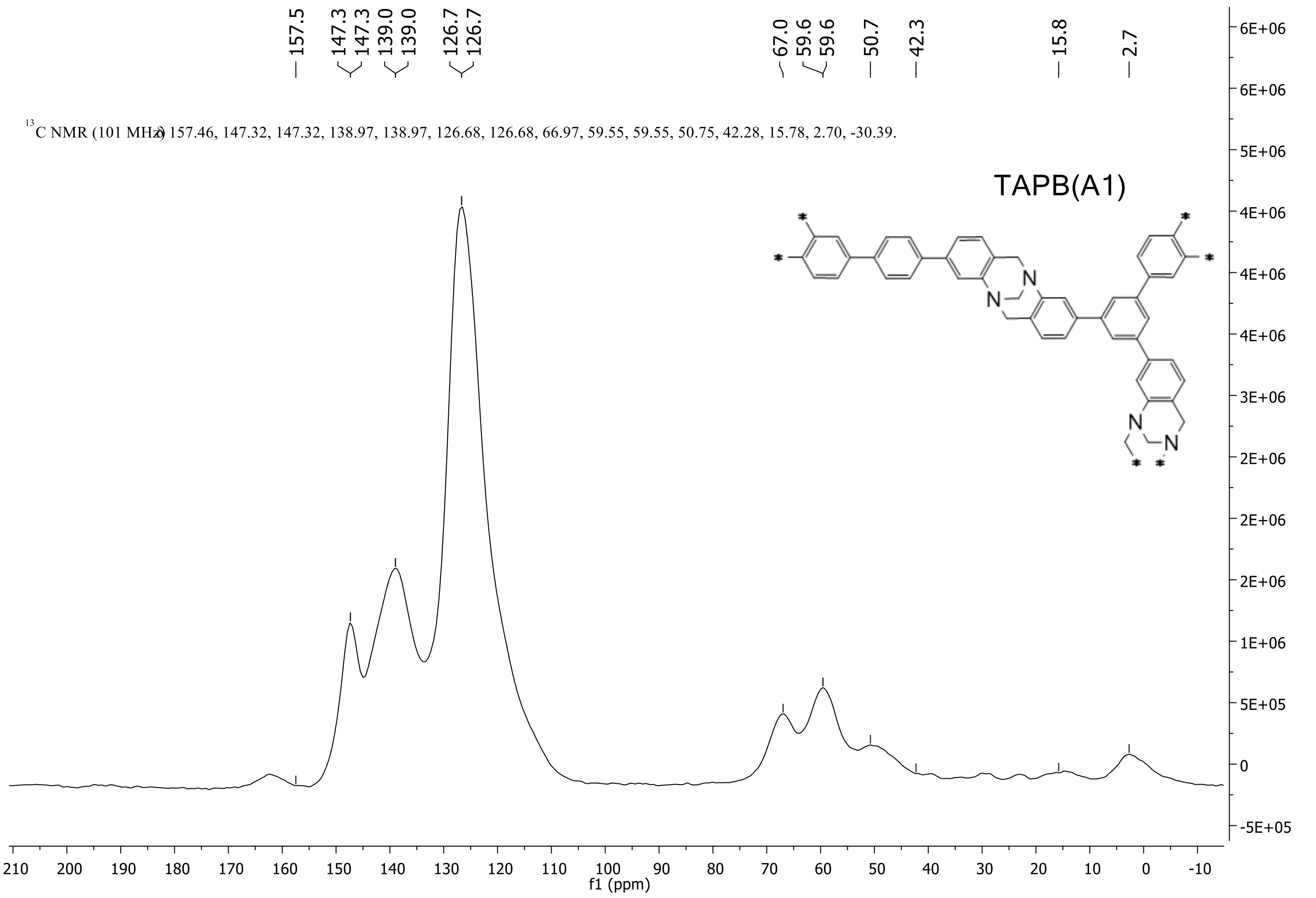

157.5
147.3
147.3
139.0
139.0
126.7
126.7
67.0
59.6
59.6
50.7
42.3
15.8
2.7
13C NMR (101 MHz) 157.46, 147.32, 147.32, 138.97, 138.97, 126.68, 126.68, 66.97, 59.55, 59.55, 50.75, 42.28, 15.78, 2.70, -30.39.
TAPB(A1)
f1 (ppm)


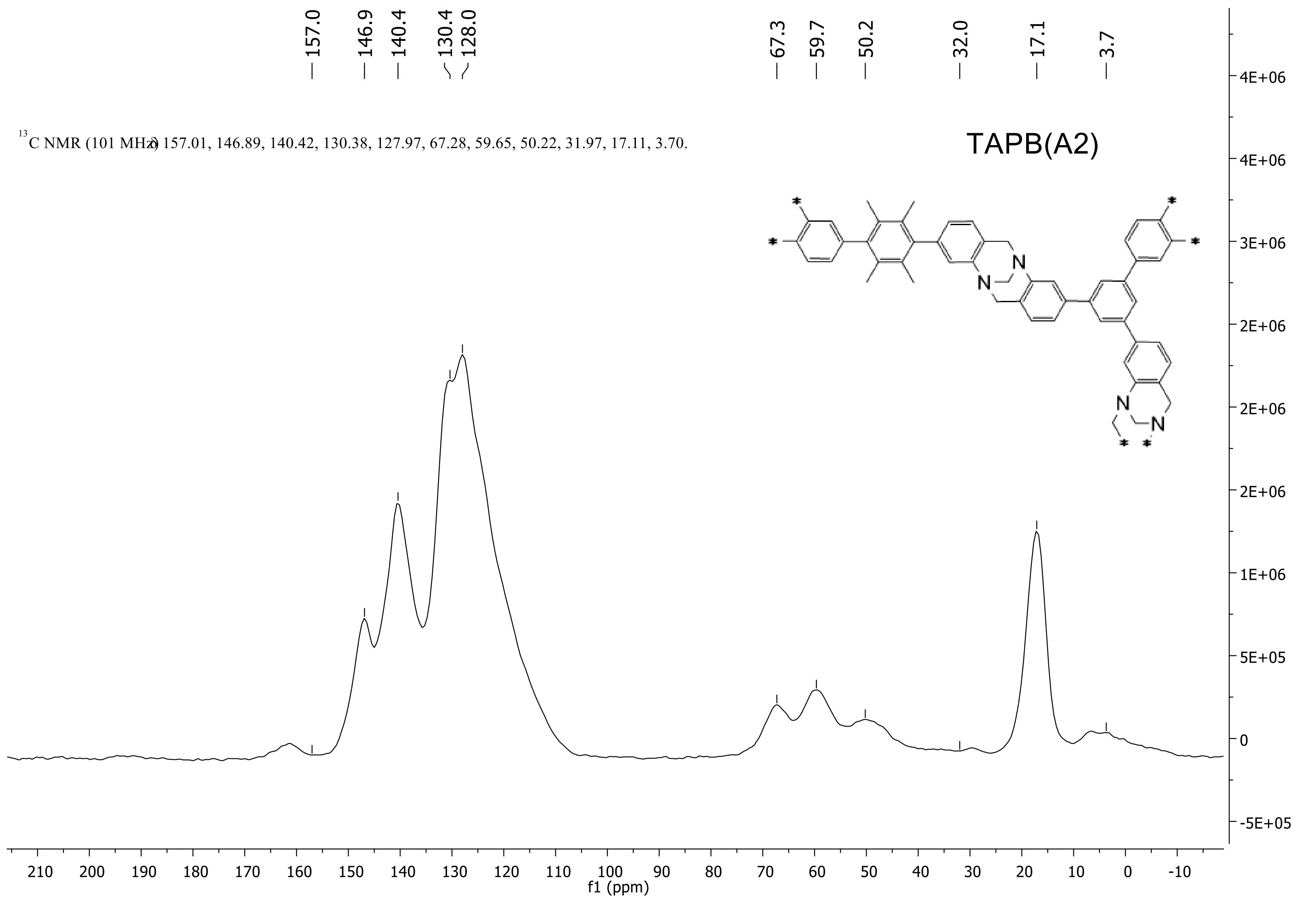

157.0
146.9
140.4
130.4
128.0
67.3
59.7
50.2
32.0
17.1
3.7
13C NMR (101 MHz) 157.01, 146.89, 140.42, 130.38, 127.97, 67.28, 59.65, 50.22, 31.97, 17.11, 3.70.
TAPB(A2)
f1 (ppm)

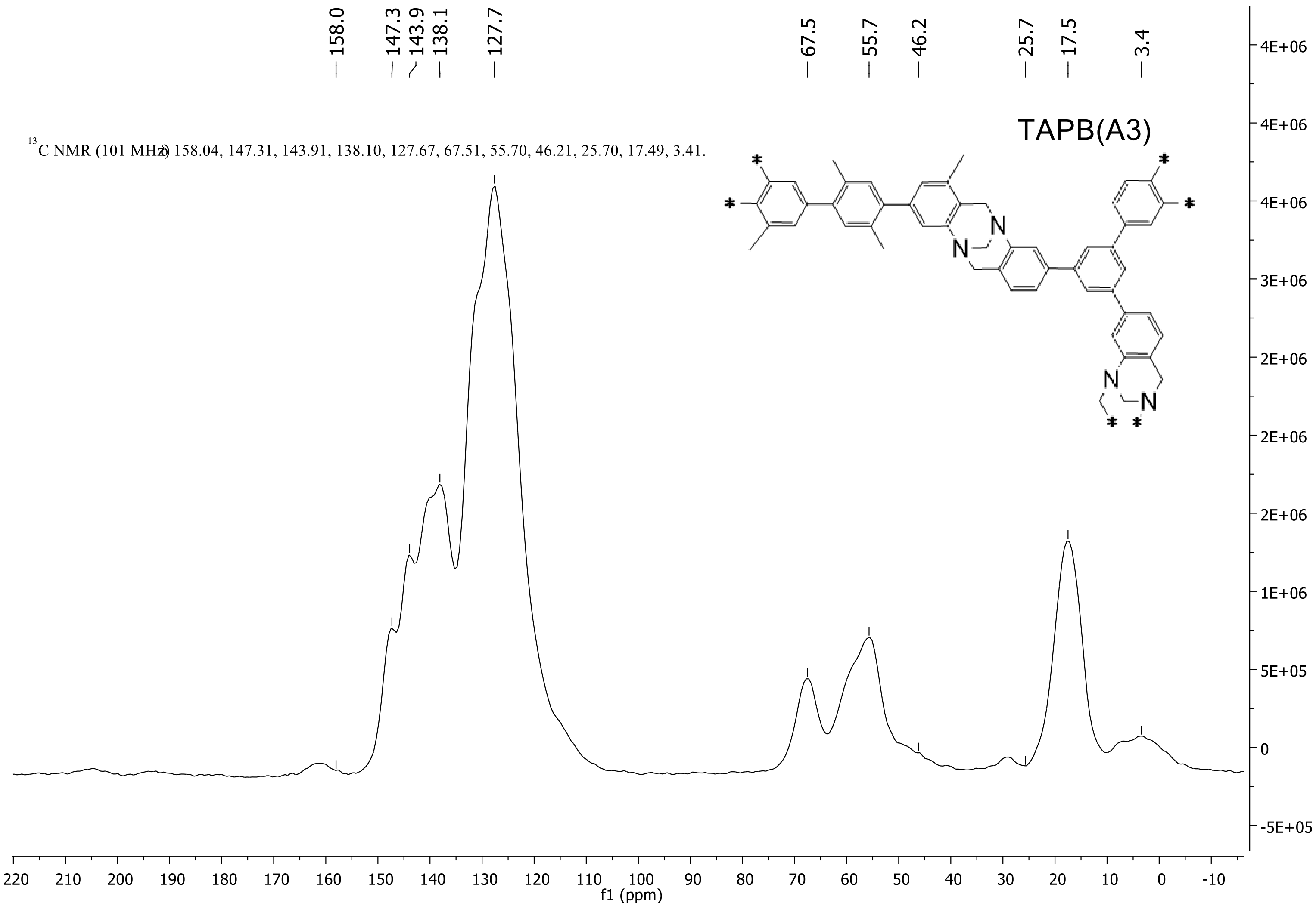
158.0
147.3
143.9
138.1
127.7
67.5
55.7
46.2
25.7
17.5
3.4
TAPB(A3)
$^{13}$C NMR (101 MHz) δ 158.04, 147.31, 143.91, 138.10, 127.67, 67.51, 55.70, 46.21, 25.70, 17.49, 3.41.
f1 (ppm)

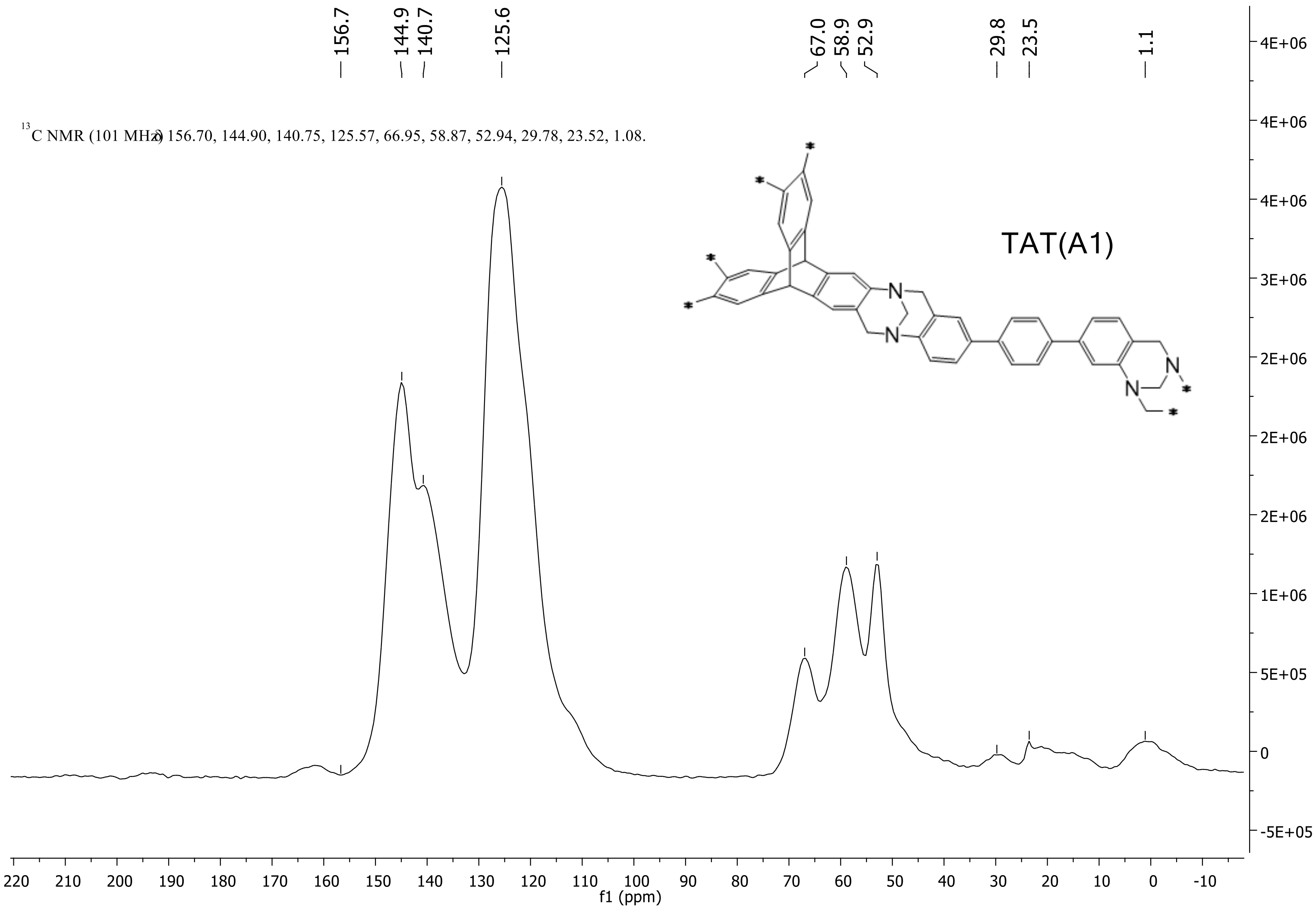
156.7
144.9
140.7
125.6
67.0
58.9
52.9
29.8
23.5
1.1
TAT(A1)
$^{13}$C NMR (101 MHz) δ 156.70, 144.90, 140.75, 125.57, 66.95, 58.87, 52.94, 29.78, 23.52, 1.08.
f1 (ppm)

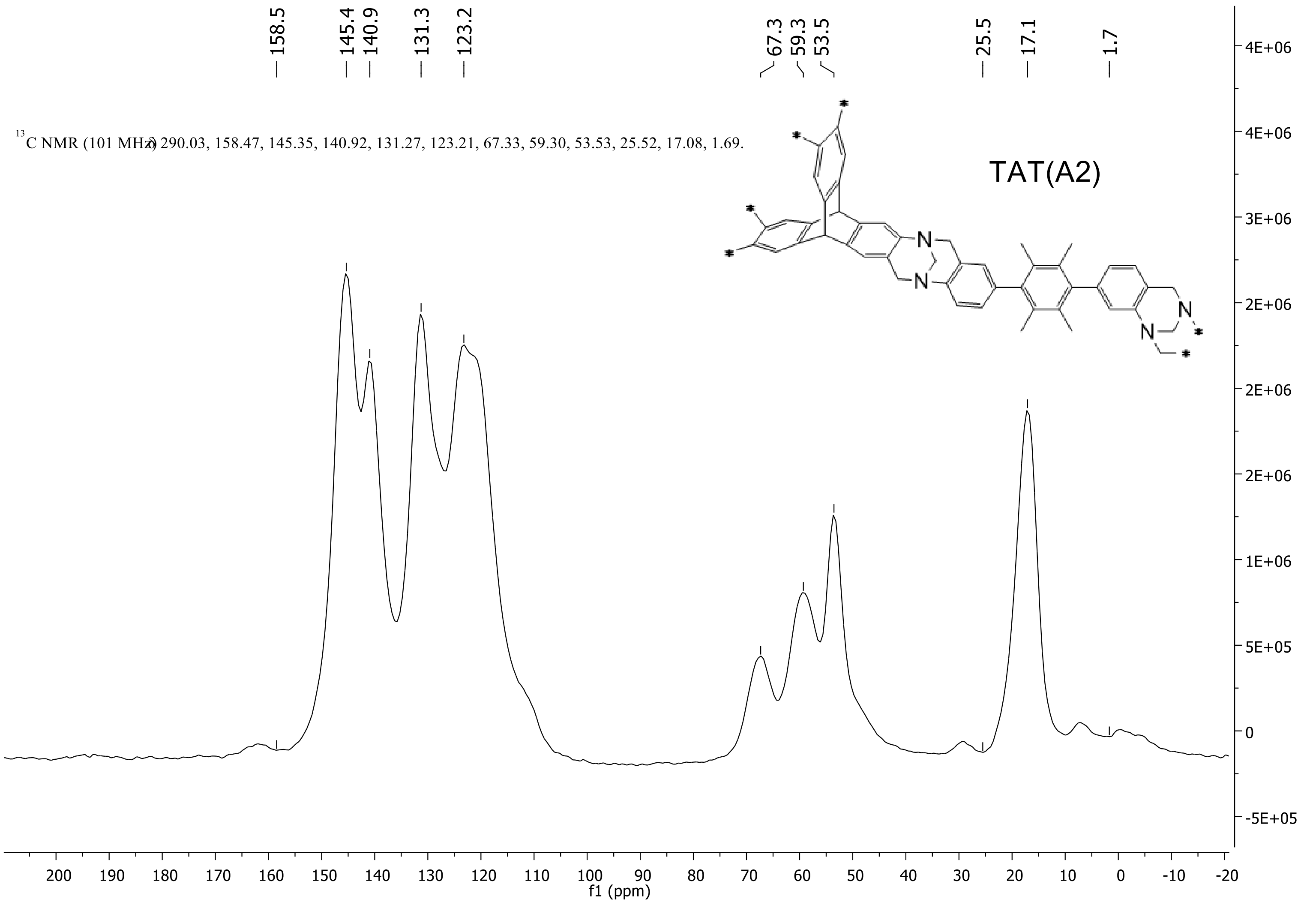
158.5
145.4
140.9
131.3
123.2
67.3
59.3
53.5
25.5
17.1
1.7
$^{13}$C NMR (101 MHz) 290.03, 158.47, 145.35, 140.92, 131.27, 123.21, 67.33, 59.30, 53.53, 25.52, 17.08, 1.69.
TAT(A2)
f1 (ppm)

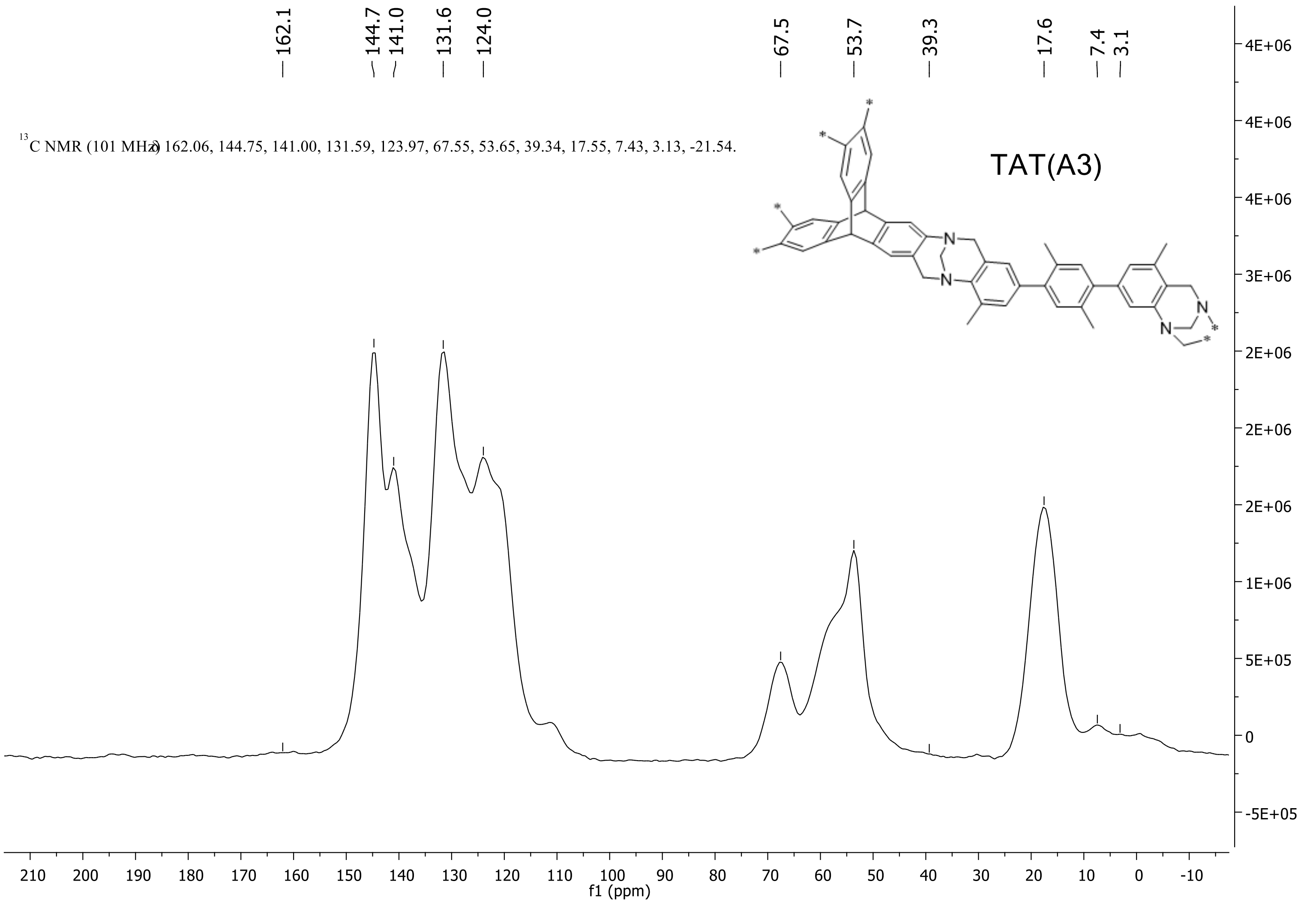
162.1
144.7
141.0
131.6
124.0
67.5
53.7
39.3
17.6
7.4
3.1
$^{13}$C NMR (101 MHz) 162.06, 144.75, 141.00, 131.59, 123.97, 67.55, 53.65, 39.34, 17.55, 7.43, 3.13, -21.54.
TAT(A3)
f1 (ppm)

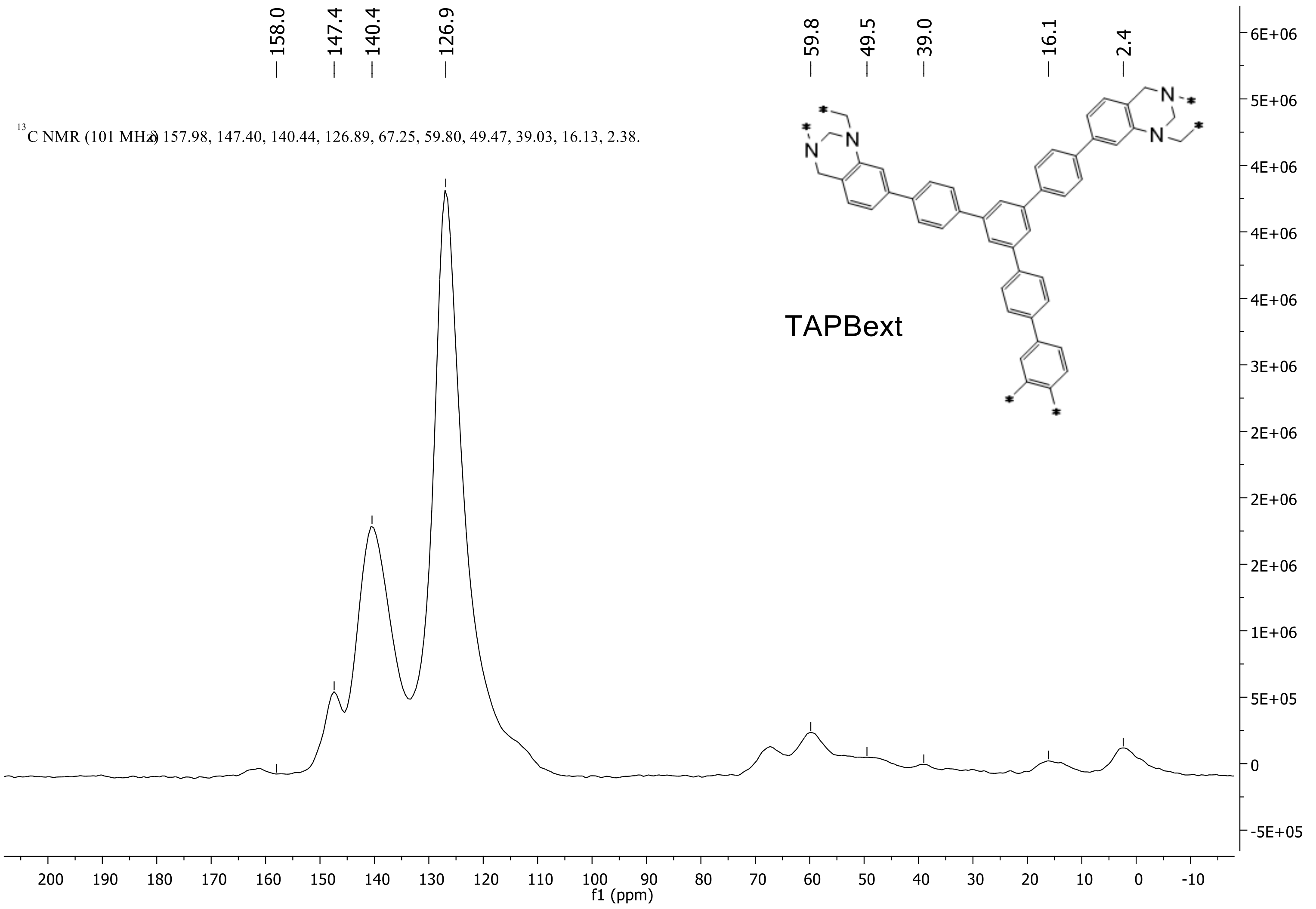
158.0
147.4
140.4
126.9
59.8
49.5
39.0
16.1
2.4
13C NMR (101 MHz) 157.98, 147.40, 140.44, 126.89, 67.25, 59.80, 49.47, 39.03, 16.13, 2.38.
TAPBext
f1 (ppm)

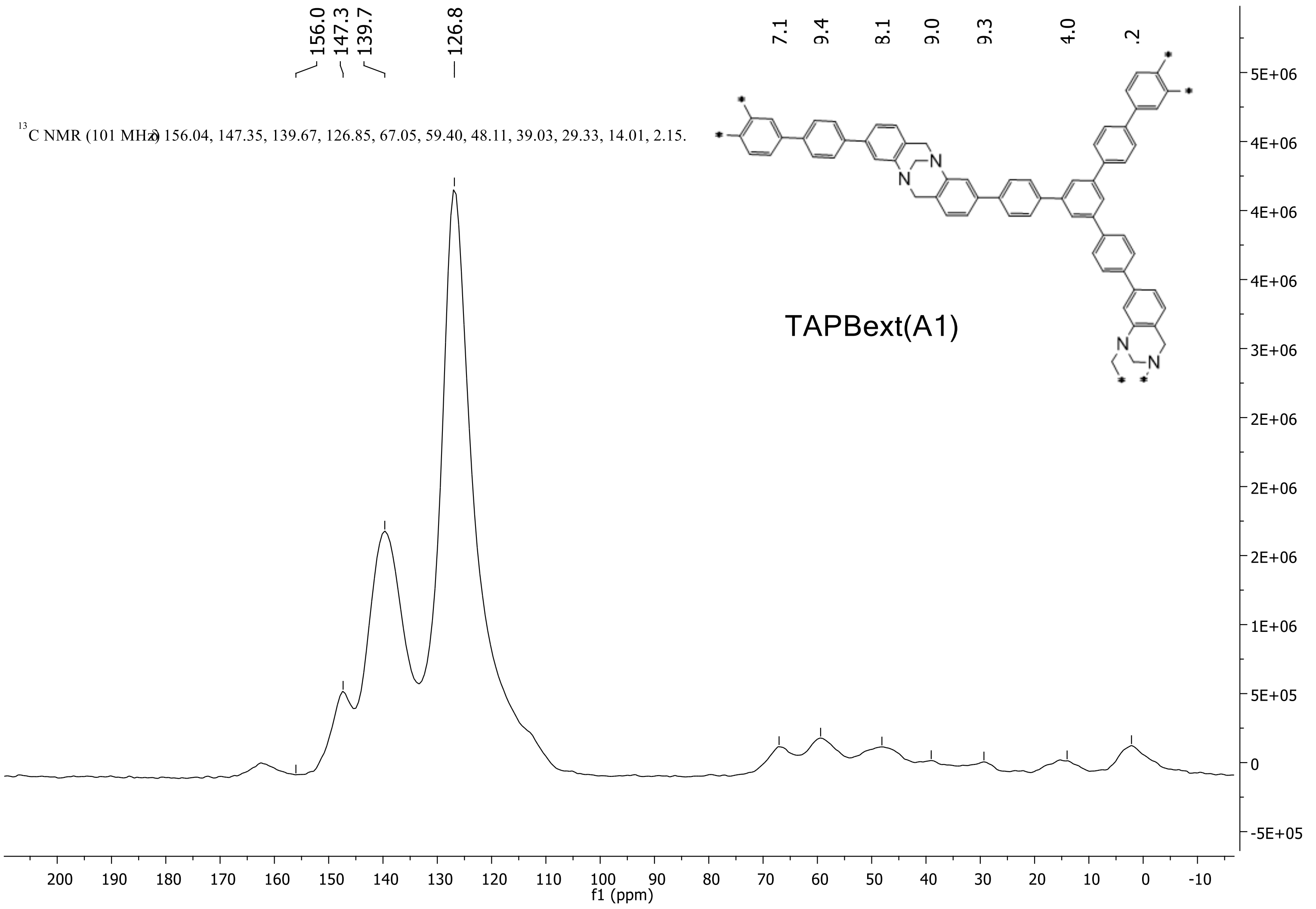
156.0
147.3
139.7
126.8
13C NMR (101 MHz) 156.04, 147.35, 139.67, 126.85, 67.05, 59.40, 48.11, 39.03, 29.33, 14.01, 2.15.
TAPBext(A1)
f1 (ppm)

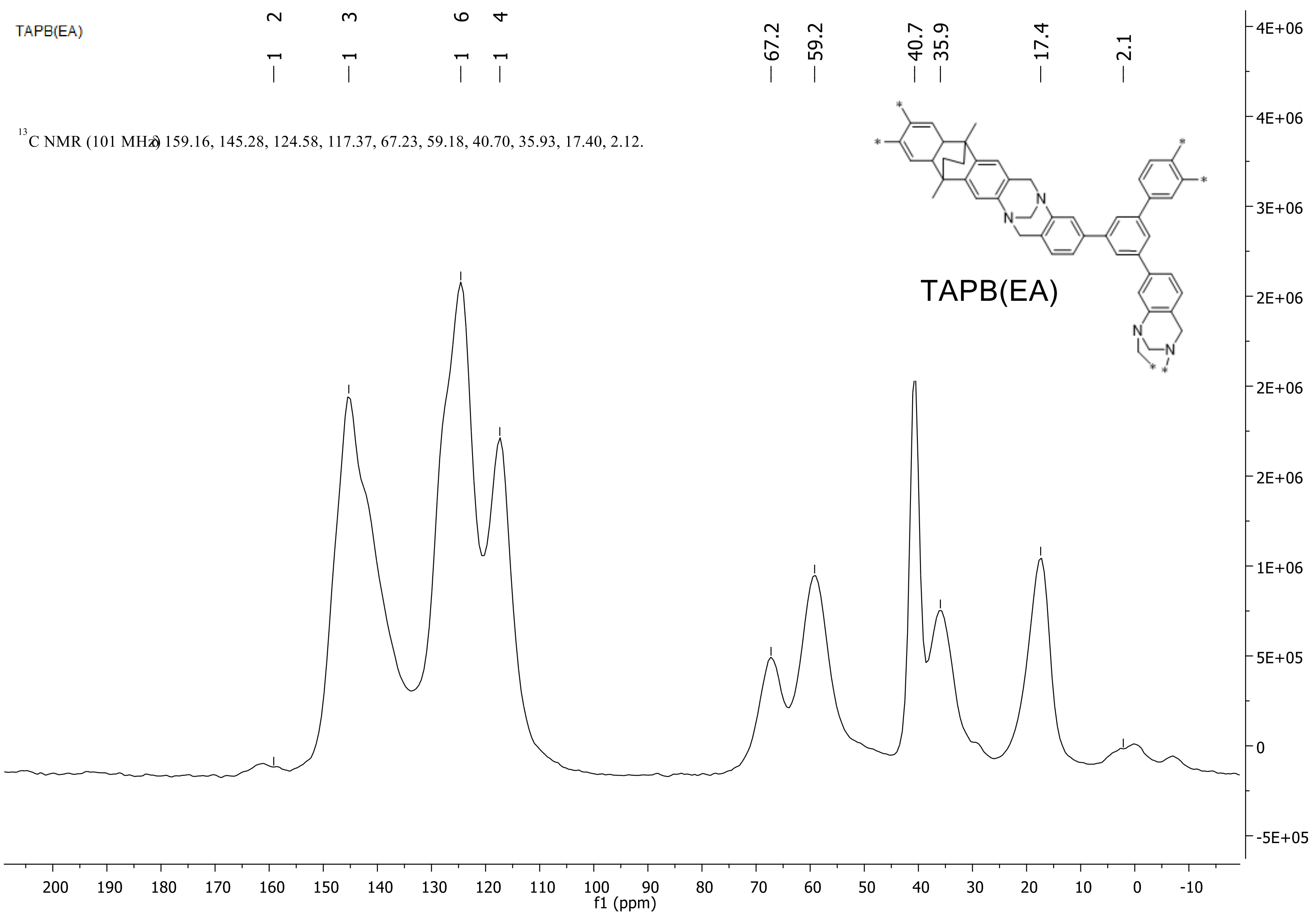
TAPB(EA)
13C NMR (101 MHz) 159.16, 145.28, 124.58, 117.37, 67.23, 59.18, 40.70, 35.93, 17.40, 2.12.
67.2
59.2
40.7
35.9
17.4
2.1
TAPB(EA)
f1 (ppm)

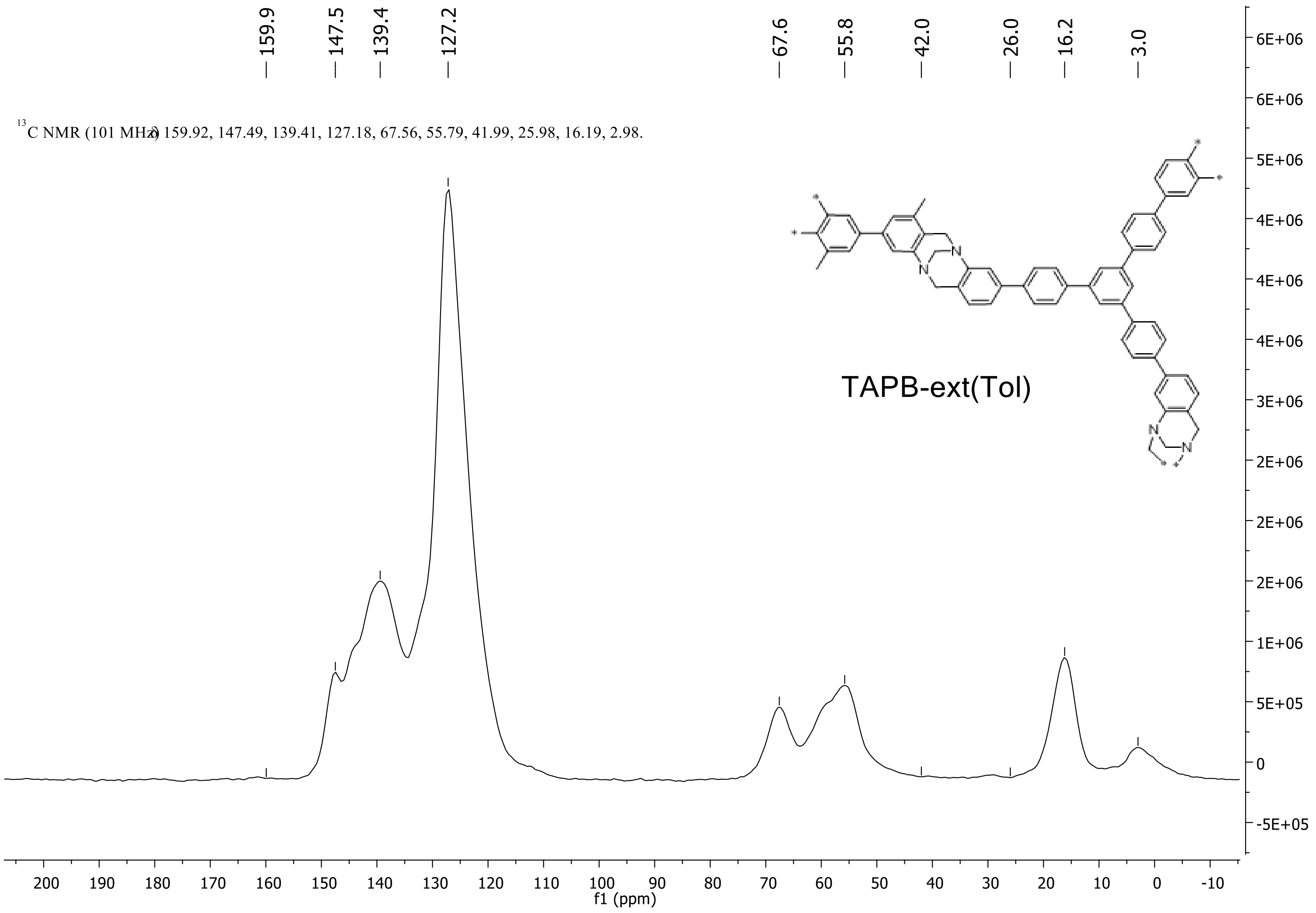
159.9
147.5
139.4
127.2
67.6
55.8
42.0
26.0
16.2
3.0
13C NMR (101 MHz) 159.92, 147.49, 139.41, 127.18, 67.56, 55.79, 41.99, 25.98, 16.19, 2.98.
TAPB-ext(Tol)
f1 (ppm)

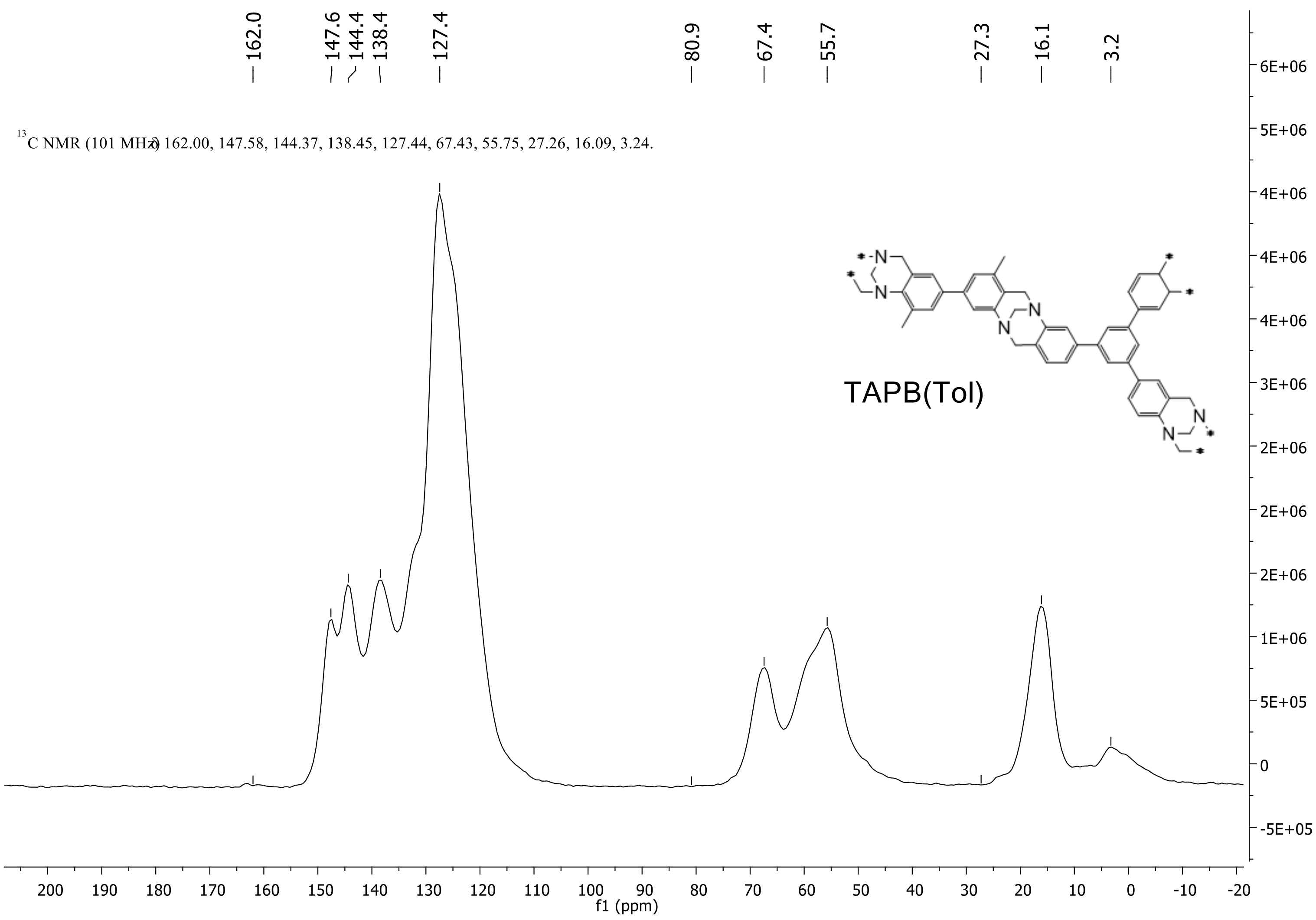
162.0
147.6
144.4
138.4
127.4
80.9
67.4
55.7
27.3
16.1
3.2
13C NMR (101 MHz) 162.00, 147.58, 144.37, 138.45, 127.44, 67.43, 55.75, 27.26, 16.09, 3.24.
TAPB(Tol)
f1 (ppm)

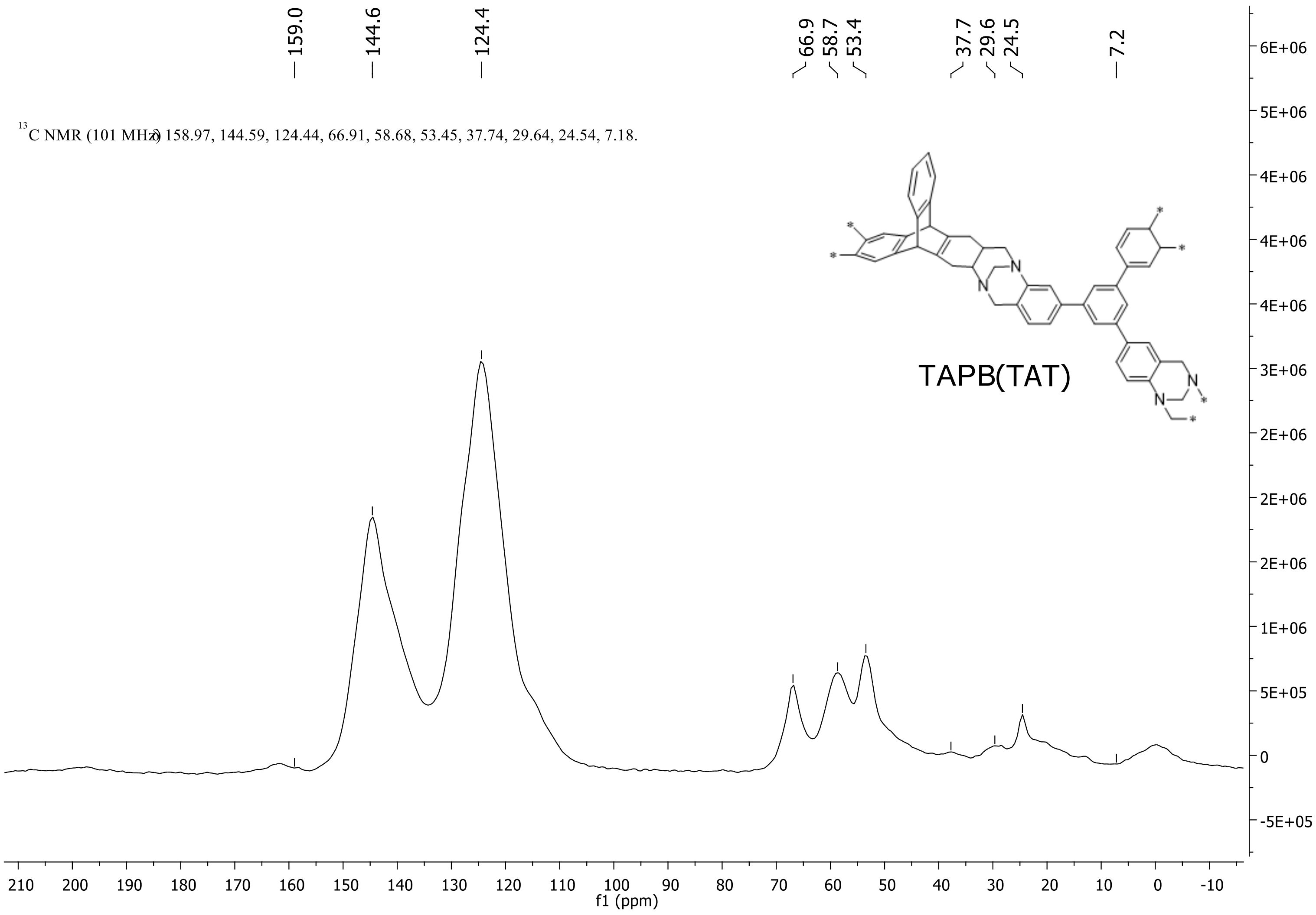
159.0
144.6
124.4
66.9
58.7
53.4
37.7
29.6
24.5
7.2
13C NMR (101 MHz) 158.97, 144.59, 124.44, 66.91, 58.68, 53.45, 37.74, 29.64, 24.54, 7.18.
TAPB(TAT)
f1 (ppm)

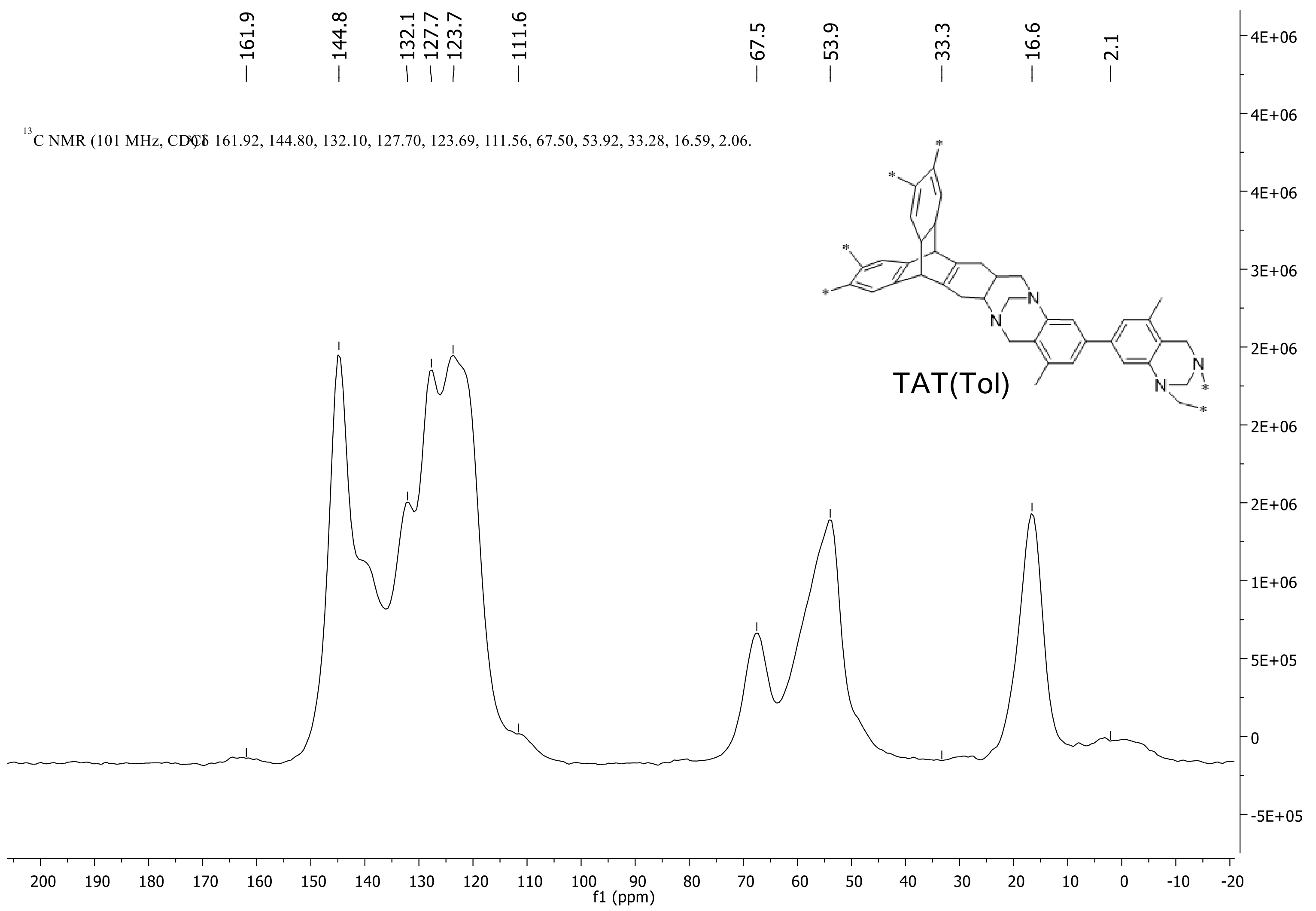


## 8. Computational models and methods

**TAPBext(A1)-PIM** and **TAPB(A1)-PIM** models and all the calculations reported was performed with BIOVIA,[7] using the Condensed-phase Optimized Molecular Potentials for Atomistic Simulation Studies[8] forcefield (COMPASS, a force field suitable to explore polymer properties, widely utilised to explore PIMs)[9-11] with forcefield assigned charges. Both Electrostatic terms and van der Waals terms were calculated with atom-based summation method, cubic spline truncation method, 12.5 Å cutoff distance, 1 Å spline width and 0.5 Å Buffer width. Dynamics parameters were 1 fs time step, Nose Thermostat[12] (Q ratio 0.01) and Berendsen barostat[13] (0.1 ps decay constant) for NPT ensembles.

**Polymeric box creation**

Since the focus of the modelling is not the polymerisation reaction itself, the process of polymer creation is hasten starting from a semi-reacted state of the monomers. In this semi-reacted state, the terminal benzene rings are substituted with a methyl group in *orto*-position to the amino group in order to straightforward link the C-N atoms already present in the structure, and then only add the -$CH_2$ group to complete the Tröger's base chemistry. The polymers model built in this work are all A-B copolymer, where A-A and B-B links are forbidden.

To enhance the formation of a highly networked polymer, some small seeds structures are created to grow the polymer from. The seed is formed by a central trifunctional monomer, surrounded by a first "shell" of bifunctional monomers and a second "shell" of trifunctional monomers. In the seed core, each functional group is reacted, part from the six outermost groups of the terminal monomers.

Using the seed as the starting point of chain growth, an iterative process is run. At each step the polymer chain (i.e. the seed for the first iteration or the previous step output for successive iterations) is arranged in an amorphous box together with a defined number of free monomers at a density of 1 g $cm^{-3}$, the details are illustrated in **Table ESI 6**. The Amorphous Cell algorithm locate all the polymeric chains in the box, growing them one segment at the time, by Monte Carlo moves (the Monte Carlo algorithm probability is calculated with respect to Flory's RIS theory).[14] A series of short NVT dynamics (with fixed amount of substance (N), volume (V) and temperature (T)) are performed and after each dynamic the mutual spatial arrangement of the free monomers and the chain's free functional groups are investigated. If a free monomer and a chain reactive group get sufficiently close to each other, and with a suitable orientation, a Tröger's base link between the two is created. If no bond is created after 4

iterations the system is heated up to 500 K for 10 ps and then cooled at 300 K for 0.1 ps. During the growth process, the free monomers are free to link to the existing chain with one or more of their reactive sites, therefore it can happen that two outermost reactive groups of the chain get linked by a bridging free monomer. At the end of each step, all monomers not linked to the main polymer chain are deleted. In the third step of the process, the amorphous box is filled with both bi- and tri-functional monomers to promote the formation of side chains, while in the last step 20 bivalent monomers are inserted.

**Table ESI 8.** Details of chain growth procedure (the images reported represents the formation of the second sample of TAPBext(A1)-PIM polymer)

**<u>Actions per step</u>**

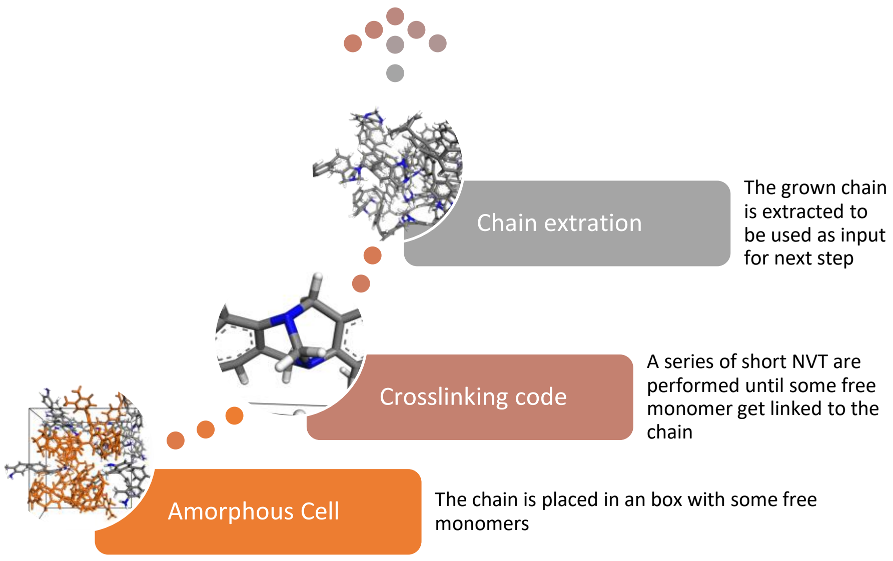


**<u>Steps outputs</u>**

| Step 0 | Step 1 | Step 2 | Step 3 | Step 4 |
|---|---|---|---|---|

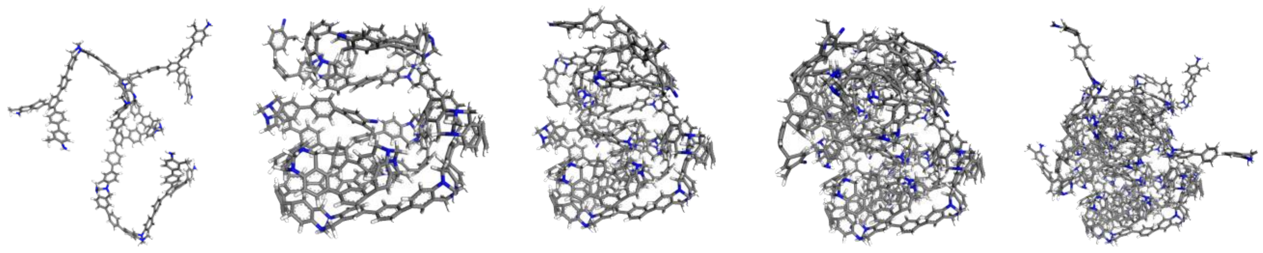

| | | | | | | | | |
|---|---|---|---|---|---|---|---|---|
| Core | → | + 10 bifunctional monomers | → | + 10 trifunctional monomers | → | + 10 bifunctional monomers +10 trifunctional monomers | → | + 20 bifunctional monomers |

**Polymeric boxes**

2 samples of each investigated polymer were modelled. The chains details are reported in **Table ESI 7**.

**Table ESI 9.** Polymer chain details

| **Polymer** | **TAPBext(A1)-PIM** | **TAPBext(A1)-PIM** | **TAPB(A1)-PIM** | **TAPB(A1)-PIM** |
|---|---|---|---|---|
| *Sample* | 1 | 2 | 1 | 2 |
| *N° of atoms* | 1677 | 1869 | 1641 | 1269 |
| *N° of bifunctional monomers* | 20 | 23 | 13 | 18 |
| *N° of trifunctional monomers* | 11 | 12 | 25 | 11 |
| *N° Terminal $NH_2$ groups* | 7 | 12 | 11 | 7 |

**The slight changes in the structures reflect the good randomness of the polymer creation process.**

**Hydrated boxes creation**

In order to study how the polymer structure changes with respect to its degree of swelling, considering both solvent mixtures, polymeric box at different density are packed with the same solution at density 1 g cm $^{-3}$.

The solvent mixture used is Ethanol, 4-tert-butylbenzaldehyde and Malononitrile in the ratio 6.8: 1:1 and 0:3:1, the same used for the experimental tests.

Two hydrated samples box are realised by firstly creating an amorphous box containing only the polymer at a given density, then the void space of the box is isolated tracing an isosurface on the van der Waal radii of the chain atoms. Finally, the void space inside the isosurface is packed with the mixture molecules at the desired ratio and density 1 g cm $^{-3}$. This value is chosen because preliminary tests performed on the mixture 6.8:1:1 alone show that its density is slightly below 1 g cm $^{-3}$.

Each concentration is equivalent to the various degree of swelling when the solvent is inserted. The higher value is chosen from preliminary test on the anhydrous polymer. For TAPBext(A1)-PIM polymer the amorphous cell densities were 1, 0.8, 0.6, 0.4 and 0.2, and the corresponding degrees of swelling were 0, 20, 40, 60 and 80 %, respectively. For TAPB(A1)-PIM the densities were 1.2, 1, 0.8, 0.6, 0.4 and 0.2 and the degrees of swelling were 0, 17, 33, 50, 67 and 83%.

A 300 ps NVT dynamics was performed to each structure at 323 K in order to promote molecules motion before the long equilibration step of 2 ns NPT dynamics at 298 K and 1 bar. At the end of the process, some structural properties of the boxes are evaluated.

**Structural characterization**

The molecular conformation behaviour of polymer chains is analysed by calculating the radius of gyration, ($R_g$), which gives a sense of the size of the polymer coil and is defined as:

$$R_g = \langle R_g^2 \rangle = \frac{1}{N} \langle \sum_{i=1}^{N} |\boldsymbol{r}_i - \boldsymbol{r}_{c.m.}|^2 \rangle$$

where $\boldsymbol{r}_i$ and $\boldsymbol{r}_{c.m.}$ represent the position vector of the $i$th atom and the center of mass of the polymer chain, respectively. The radius of gyration is also the quantity that is experimentally accessed.

**Table ESI 10** Radii of gyration for hydrated boxes in mixtures of Ethanol, 4-tert-butylbenzaldehyde and Malononitrile in the ratio 6.8: 1:1

| **polymer** | **Degree of swelling** | | | | | |
|---|---|---|---|---|---|---|
| **TAPB-EA1** | 0 | 17 | 33 | 50 | 67 | 83 |
| *Radius of gyration* | 12.81 | 13.26 | 14.65 | 14.91 | 16.96 | 19.50 |
| *Dev.st.* | 0.00 | 0.25 | 0.93 | 1.29 | 1.24 | 0.28 |
| **TAPB-ext(A1)-PIM** | 0 | 20 | 40 | 60 | 80 | - |
| *Radius of gyration* | 15.08 | 15.07 | 15.64 | 17.70 | 22.13 | - |
| *Dev.st.* | 0.68 | 0.57 | 0.81 | 0.01 | 0.40 | - |

**Table ESI 11** Radii of gyration for hydrated boxes in mixtures of 4-tert-butylbenzaldehyde and Malononitrile in the ratio 3:1

| **polymer** | **Degree of swelling** | | | | | |
|---|---|---|---|---|---|---|
| **TAPB-EA1** | 0 | 17 | 33 | 50 | 67 | 83 |
| *Radius of gyration* | 12.98 | 12.50 | 13.88 | 14.10 | 15.95 | 18.69 |
| *Dev.st.* | 0.03 | 0.32 | 0.21 | 1.55 | 0.04 | 0.46 |
| **TAPB-ext(A1)-PIM** | 0 | 20 | 40 | 60 | 80 | - |
| *Radius of gyration* | 15.80 | 17.00 | 16.94 | 19.21 | 15.80 | - |
| *Dev.st.* | 0.06 | 0.25 | 0.69 | 0.73 | 0.06 | - |

# 9. References


1. W. Q. Xu, Y. Z. Fan, H. P. Wang, J. Teng, Y. H. Li, C. X. Chen, D. Fenske, J. J. Jiang and C. Y. Su, *Chemistry –A European Journal*,Investigation of Binding Behavior between Drug Molecule 5 - Fluoracil and M4L4 - Type Tetrahedral Cages: Selectivity, Capture, and Release, 2017, **23**, 3542-3547.
2. R. Z. Lange, G. Hofer, T. Weber and A. D. Schlüter, *Journal of the American Chemical Society*,A two-dimensional polymer synthesized through topochemical [2+ 2]-cycloaddition on the multigram scale, 2017, **139**, 2053-2059.
3. D. A. Vazquez-Molina, G. M. Pope, A. A. Ezazi, J. L. Mendoza-Cortes, J. K. Harper and F. J. Uribe-Romo, *Chemical Communications*,Framework vs. side-chain amphidynamic behaviour in oligo-(ethylene oxide) functionalised covalent-organic frameworks, 2018, **54**, 6947-6950.
4. W. Meng, J. K. Clegg, J. D. Thoburn and J. R. Nitschke, *Journal of the American Chemical Society*,Controlling the transmission of stereochemical information through space in terphenyl-edged Fe4L6 cages, 2011, **133**, 13652-13660.
5. S. Grosjean, Z. Hassan, C. Wöll and S. Bräse, *European Journal of Organic Chemistry*,Diverse Multi - Functionalized Oligoarenes and Heteroarenes for Porous Crystalline Materials, 2019, **2019**, 1446-1460.
6. M. Carta, M. Croad, K. Bugler, K. J. Msayib and N. B. McKeown, *Polymer Chemistry*,Heterogeneous organocatalysts composed of microporous polymer networks assembled by Troger's base formation, 2014, **5**, 5262-5266.
7. P. U. G. P. S. BIOVIA,(ex Material Studio 7.0) package, Classical simulation theory section, Accelrys Software Inc., 2013.
8. S. Hai, *The Journal of Physical Chemistry B*,An ab initio force-field optimized for condensed-phase applications overview with details on alkane and benzene compounds, 1998, **102**, 7338-7364.
9. B. Satilmis, M. Lanč, A. Fuoco, C. Rizzuto, E. Tocci, P. Bernardo, G. Clarizia, E. Esposito, M. Monteleone and M. Dendisová, *Journal of Membrane Science*,Temperature and pressure dependence of gas permeation in amine-modified PIM-1, 2018, **555**, 483-496.
10. I. Hossain, S. Y. Nam, C. Rizzuto, G. Barbieri, E. Tocci and T.-H. Kim, *Journal of Membrane Science*,PIM-polyimide multiblock copolymer-based membranes with enhanced CO2 separation performances, 2019, **574**, 270-281.
11. A. Fuoco, B. Satilmis, T. Uyar, M. Monteleone, E. Esposito, C. Muzzi, E. Tocci, M. Longo, M. P. De Santo and M. Lanč, *Journal of Membrane Science*,Comparison of pure and mixed gas permeation of the highly fluorinated polymer of intrinsic microporosity PIM-2 under dry and humid conditions: Experiment and modelling, 2020, **594**, 117460.
12. G. J. Martyna, M. L. Klein and M. Tuckerman, *The Journal of chemical physics*,Nosé–Hoover chains: The canonical ensemble via continuous dynamics, 1992, **97**, 2635-2643.
13. H. Berendsen and J. Postma, *J Chem Phys*,M; DiNola, A.; Van Gunsteren, WF; Haak, 1984, **181**, 3684-3690.
14. P. J. Flory and M. Volkenstein, *Biopolymers*,Statistical Mechanics of Chain Molecules (Hanser & Gardner, Cincinnati, Ohio, 1989), 1969, **8**, 699.
